# Kinome-Wide RNA Interference Screening Identifies Mitogen-Activated Protein Kinases and Phosphatidylinositol Metabolism as Key Factors for Rabies Virus Infection

**DOI:** 10.1128/mSphere.00047-19

**Published:** 2019-05-22

**Authors:** Benoit Besson, Seonhee Kim, Taehee Kim, Yoonae Ko, Sangchul Lee, Florence Larrous, Jihwan Song, David Shum, Regis Grailhe, Hervé Bourhy

**Affiliations:** a Institut Pasteur, Unité Lyssavirus, épidémiologie et neuropathologie, Paris, France; b Sorbonne Paris Cité, Cellule Pasteur, Université Paris Diderot, Paris, France; c Technology Development Platform, Institut Pasteur Korea, Seongnam-si, Republic of Korea; d Screening Discovery Platform, Institut Pasteur Korea, Seongnam-si, Republic of Korea; e CHA Bio Complex, CHA Stem Cell Institute, Seongnam-si, Republic of Korea; Icahn School of Medicine at Mount Sinai

**Keywords:** RNA interference, drug screening, inositol phosphate phosphatases, mitogen-activated protein kinases, rabies

## Abstract

Throughout the rabies virus (RABV) infectious cycle, host-virus interactions define its capacity to replicate, escape the immune response, and spread. As phosphorylation is a key regulatory mechanism involved in most cellular processes, kinases represent a target of choice to identify host factors required for viral replication. A kinase and phosphatase small interfering RNA (siRNA) high-content screening was performed on a fluorescent protein-recombinant field isolate (Tha RABV). We identified 57 high-confidence key host factors important for RABV replication with a readout set at 18 h postinfection and 73 with a readout set at 36 h postinfection, including 24 common factors at all stages of the infection. Amongst them, gene clusters of the most prominent pathways were determined. Up to 15 mitogen-activated protein kinases (MAPKs) and effectors, including MKK7 (associated with Jun N-terminal protein kinase [JNK] signalization) and DUSP5, as well as 17 phosphatidylinositol (PI)-related proteins, including PIP5K1C and MTM1, were found to be involved in the later stage of RABV infection. The importance of these pathways was further validated, as small molecules Ro 31-8820 and PD 198306 inhibited RABV replication in human neurons.

**IMPORTANCE** Rabies virus relies on cellular machinery for its replication while simultaneously evading the host immune response. Despite their importance, little is known about the key host factors required for rabies virus infection. Here, we focused on the human kinome, at the core of many cellular pathways, to unveil a new understanding of the rabies virus infectious cycle and to discover new potential therapeutic targets in a small interfering RNA screening. The mitogen-activated protein kinase pathway and phosphatidylinositol metabolism were identified as prominent factors involved in rabies virus infection, and those findings were further confirmed in human neurons. While bringing a new insight into rabies virus biology, we also provide a new list of host factors involved in rabies virus infection.

## Abstract (precis)

Rabies virus relies on cellular machinery for its replication while simultaneously evading the host immune response. Despite their importance, little is known about the key host factors required for rabies virus infection. Here, we focused on the human kinome, at the core of many cellular pathways, to unveil a new understanding of the rabies virus infectious cycle and to discover new potential therapeutic targets in a small interfering RNA screening. The mitogen-activated protein kinase pathway and phosphatidylinositol metabolism were identified as prominent factors involved in rabies virus infection, and those findings were further confirmed in human neurons. While bringing a new insight into rabies virus biology, we also provide a new list of host factors involved in rabies virus infection.

## INTRODUCTION

Rabies virus (RABV), the type species of the *Lyssavirus* genus, is a zoonotic agent causing acute encephalitis and is responsible for about 60,000 deaths/year (1). The cost of and limited access to health care and the lack of a public health agenda make rabies a burden that mostly affects rural communities in Asia and Africa, where children are the principal victims of the virus (2). Therefore, more than a century after the discovery of a RABV vaccine that is effective as a postexposure treatment (3), reaching a better understanding of RABV biology and finding new therapeutic targets remain priorities.

RABV is a single-stranded negative RNA virus (order *Mononegavirales*, family *Rhabdoviridae*) with a 12-kb genome coding for 5 proteins. The nucleoprotein (N) encapsulates the genomic viral RNA, which constitutes a ribonucleoprotein (RNP). The phosphoprotein (P) and the RNA-dependent RNA polymerase (L) bind to the RNP, forming the replication and transcription complex (4, 5). The glycoprotein (G) is inserted into the envelope of the viral particle and acts as a binding and fusion protein for the entry of the virus into the host cell. The matrix protein (M) links the N protein involved in the RNP to the internal domain of the G protein, shaping the viral particle in a “bullet” shape as well as playing a crucial role in the entry and budding phases, notably hijacking the endosomal sorting complex required for transport or endosomal sorting complexes required for transport (ESCRT) (6). Together with the N protein, the M protein also regulates the balance between replication and transcription (7). Moreover, all viral proteins harbor secondary functions to hijack the cellular machinery. The N and L proteins are notably involved in hiding viral RNAs from cell sensors (8, 9). Further, both the G and M proteins participate in the regulation of cell death, interfering with MAST2, protein tyrosine-phosphatase nonreceptor 4 (PTPN4), TRAIL, and cytochrome *c* oxidase signaling (10–12). Finally, the P and M proteins hijack the IRF (IκB kinase ε [IKKε], TBK1, IRF3), NF-κB (RelAp43, p105/p50, ABIN2), JAK-STAT (JAK1, STAT1, STAT2, STAT3), and possibly the mitogen-activated protein kinase (MAPK) (TPL2) pathways in order to control the immune response (13–19).

Large-scale screening approaches allow a better understanding of the complex nature of virus-host interactions through the identification of key host factors. Transcriptomic (20–23) and proteomic (24–26) experiments identified differentially expressed genes and proteins in RABV-infected cells but generally lacked a clear functional relevance to pathogenesis (23). A yeast two-hybrid screening determined that the focal adhesion kinase (FAK) interacts with the P protein, and a cell-free protein synthesis screening identified ATP-binding cassette family E1 (ABCE1) as a possible factor for capsid formation such as is observed with HIV (27, 28). Recently, a first loss-of-function screening was performed on 3,200 mutant neurons differentiated from murine embryonic stem cells using the SPBN-Nfu-GFP laboratory strain (29). The spreading capacity of green fluorescent protein (GFP)-labeled RABV was investigated at 24, 48, and 72 h, and 63 hits were found, among which 2 targets were validated: Unc13d and Bbs4 (the 61 other hits were neither confirmed nor invalidated). A gene ontology (GO) analysis assigned the 63 hits to the following GO terms: induction of apoptosis, G-protein-coupled receptor binding, and perinuclear region of cytoplasm and ubiquitin-specific protease/thioesterase activity. Further, a genome-wide screening focusing on the late stage of several negative-strand RNA viruses (30) identified nearly 72 important genes for vesicular stomatitis virus (VSV; *Rhabdoviridae*), among which 25 were also identified as important for lymphocytic choriomeningitis virus (LCMV; *Arenaviridae*) and human parainfluenza virus type 3 (HPIV3; *Paramyxoviridae*). A second genome-wide screening was performed on the early stages of VSV infection (31) and identified 300 host genes important for VSV, among which 23 were validated. Taken together, the following 8 common hits were identified as key factors for the early and late VSV infection: ARCN1, COPB1, COPG, COPZ1, MAT2A, NHP2L1, SYVN1, and UTP6.

In the light of the many interactions between rabies virus proteins and cell signaling pathways, we designed a functional genomic approach based on high-content screening using a kinase and phosphatase small interfering RNA (siRNA) library on the fluorescent protein-recombinant field isolate Tha RABV to identify cellular targets involved in the RABV infectious cycle. A total of 106 key host factors were identified in HEK293T cells as “viral helpers” or “viral inhibitors” at early (18 h) or late (36 h) stages of the infection. Taking the results together, the screening provided a list of targets involved in the early and late-stage replication cycles of field isolate RABV.

## RESULTS

### RNA interference (RNAi) screening for kinase and phosphatase factors in field isolate RABV infection.

To identify key host factors for RABV at both the early and late stages of the infection, a kinase and phosphatase siRNA screen was performed with readout at 18 h and 36 h postinfection. The GFP-recombinant field isolate Tha virus (Tha-GFP), engineered in our laboratory, was screened against a focused library of 3,024 duplexes composed of three independent siRNAs for 710 kinase genes and 298 phosphatase genes. HEK293T cells were transfected with a single siRNA and counted 72 h later using Hoechst staining and automated microscopy (see Fig. S2A in the supplemental material). We chose to infect cells at a multiplicity of infection (MOI) of 5 in order to reach 20% of infected cells at 18 h and 70% at 36 h (Fig. S2). At the experimental endpoint, cells were fixed, nuclei were subjected to Hoechst staining, and images were acquired for both Hoechst fluorescence and GFP fluorescence by automated microscopy. Images (Fig. S2B) were analyzed to quantify the number of cell per well (Fig. S2C), the Tha-GFP intensity in each well (Fig. S2D), and the percentage of Tha-GFP positive cells (Fig. S2E).

As a functional control measuring RNAi transfection, we targeted polo-like kinase 1 (PLK1)—an essential gene for cell viability—which reduced the count of nuclei to less than 5% (Fig. S2B and C) of the count determined for the scramble siRNA (siNEG). The effective RNA interference of Tha replication was confirmed with small interfering GFP (siGFP) and with siRNA targeting the nucleolin (NCL), which was previously described as a key host factor for RABV (32). Targeting the GFP reduced the levels of Tha-GFP expression to 30% at 18 h and 7% at 36 h compared to siNEG, while the siNCL reduced cell viability to 70% only at 18 h (Fig. S2B and C) and Tha-GFP expression to 52% of the intensity at 18 h and 82% at 36 h (Fig. S2B, D, and E).

**FIG 1:**
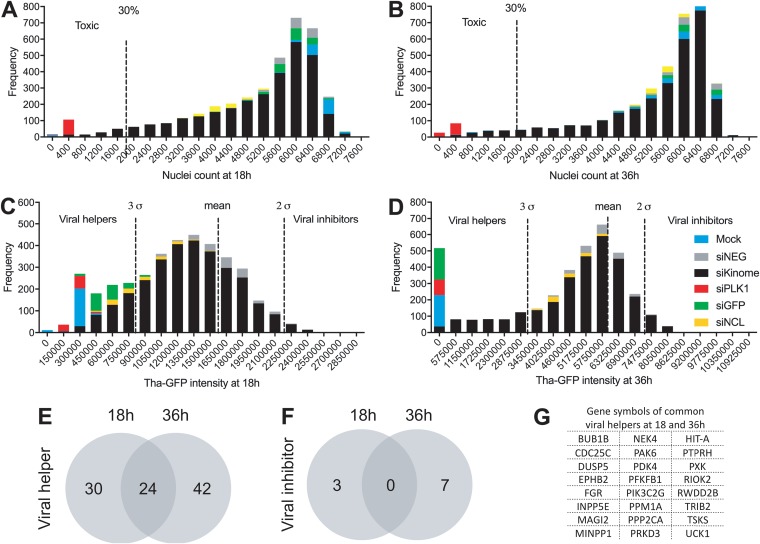
Quality control of the kinome-wide screening and identification of the major hits. (A to D) Population distribution of all hits according to the number of cells at 18 h (A) or 36 h (B) and GFP intensity at 18 h (C) or 36 h (D). The mock infection data correspond to noninfected and nontransfected cells. (E and F) Venn diagrams showing the distribution of the high-confidence hits identified as representing “viral helpers” (E) and “viral inhibitors” (F) at between 18 and 36 h. (G) List of the common hits found at both 18 and 36 h.

In order to define relevant and specific hits, we analyzed both nucleus counts and Tha-GFP intensity distributions of the population of siRNA duplexes. All siRNAs affecting cell viability at 18 h (Fig. 1A) and 36 h (Fig. 1B) were labeled toxic (threshold at 30% of siNEG count of nuclei). The mean of the siNEG Tha-GFP intensity was then used to define hits under a 3σ threshold as viral helpers and hits over a 2σ threshold as viral inhibitors at 18 h (Fig. 1C) and 36 h (Fig. 1D). The specificity of the 3σ threshold was confirmed by analysis of the controls. At 18 h postinfection, the Tha-GFP signals corresponding to 95% of the level seen with the siGFP-treated well and 45% of the level seen with the siNCL-treated well were under the 3σ threshold. At 36 h, 100% of the siGFP-treated wells but none of the siNCL-treated wells had a Tha-GFP signal under the 3σ threshold. Therefore, NCL is a key factor for Tha-GFP only in the early stages of the infection, which further validates our system. Finally, only genes confirmed with at least two of three single siRNAs were considered high-confidence key host factors (listed in Table 1 and 2) whereas genes with only one effective siRNA were labeled low-confidence factors in further analysis (listed in Table S1 and S2 in the supplemental material). Using this approach, we were able to identify 54 high-confidence viral helpers for the early stages of the infection and 66 for the late stages of the infection (Fig. 1E). In comparison, only a few high-confidence viral inhibitors were identified (3 at 18 h and 7 at 36 h) (Fig. 1F). Interestingly, 24 genes were commonly identified as high-confidence viral helpers at all stages of the infection (Fig. 1G). Furthermore, 7 high-confidence viral helpers were found to be systematically affecting RABV replication with all three siRNAs, including CDC25C, CHTF18, EPHA7, PPP2CA, and PTPRN at 18 h postinfection and DUSP5 and PRPF4B at 36 h postinfection (Table 1 and 2).

**TABLE 1:** List of high-confidence hits identified after 18 h of RABV infection^*a*^

Gene designation	Full gene name	GFP score		Effect on virus (no. showing effect/total no.)	Effect on cells	Role
		Avg	%			
CDC25C	Cell division cycle 25 homolog C	586,478	45	Inhibition (3/3)	Nontoxic	Viral helper¶
CHTF18	CTF18, chromosome transmission fidelity factor 18 homolog	547,046	42	Inhibition (3/3)	Nontoxic	Viral helper
EPHA7	EPH receptor A7	527,672	41	Inhibition (3/3)	Nontoxic	Viral helper
PPP2CA	Protein phosphatase 2, catalytic subunit, alpha isozyme	685,682	53	Inhibition (3/3)	Nontoxic	Viral helper¶
PTPRN	Protein tyrosine phosphatase, receptor type, N	685,771	53	Inhibition (3/3)	Nontoxic	Viral helper
AURKA	Aurora kinase A	675,901	52	Inhibition (2/3)	Nontoxic	Viral helper
BMP2K	BMP2 inducible kinase	647,851	50	Inhibition (2/3)	Nontoxic	Viral helper
BUB1B	Budding uninhibited by benzimidazoles 1 homolog beta	745,215	57	Inhibition (2/3)	Nontoxic	Viral helper¶
CLK1	CDC-like kinase 1	678,990	52	Inhibition (2/3)	Nontoxic	Viral helper
DUSP22	Dual-specificity phosphatase 22	689,114	53	Inhibition (2/3)	Nontoxic	Viral helper
DUSP5	Dual-specificity phosphatase 5	571,782	44	Inhibition (2/3)	Nontoxic	Viral helper*¶
DUSP7	Dual-specificity phosphatase 7	560,478	43	Inhibition (2/3)	Nontoxic	Viral helper
DUSP9	Dual-specificity phosphatase 9	578,336	44	Inhibition (2/3)	Nontoxic	Viral helper
EPHB2	EPH receptor B2	702,327	54	Inhibition (2/3)	Nontoxic	Viral helper¶
FGR	Gardner-Rasheed feline sarcoma viral (v-fgr) oncogene homolog	469,057	36	Inhibition (2/3)	Nontoxic	Viral helper¶
FRAP1	FK506 binding protein 12 rapamycin-associated protein 1	534,893	41	Inhibition (2/3)	Nontoxic	Viral helper
HDDC3	HD domain containing 3	535,343	41	Inhibition (2/3)	Nontoxic	Viral helper
ICK	Intestinal cell (MAK-like) kinase	504,853	39	Inhibition (2/3)	Nontoxic	Viral helper
INPP5E	Inositol polyphosphate-5-phosphatase	714,055	55	Inhibition (2/3)	Nontoxic	Viral helper¶
INPPL1	Inositol polyphosphate phosphatase-like 1	629,572	48	Inhibition (2/3)	Nontoxic	Viral helper
KSR1	Kinase suppressor of ras 1	500,179	38	Inhibition (2/3)	Nontoxic	Viral helper
MAGI2	Membrane associated guanylate kinase, WW and PDZ domain containing 2	715,093	55	Inhibition (2/3)	Nontoxic	Viral helper¶
MAP2K1	Mitogen-activated protein kinase kinase 1	621,239	48	Inhibition (2/3)	Nontoxic	Viral helper
MAP3K11	Mitogen-activated protein kinase kinase kinase 11	439,345	34	Inhibition (2/3)	Nontoxic	Viral helper
MINPP1	Multiple inositol polyphosphate histidine phosphatase 1	479,086	37	Inhibition (2/3)	Nontoxic	Viral helper*¶
MTMR4	Myotubularin-related protein 4	559,013	43	Inhibition (2/3)	Nontoxic	Viral helper
NEK4	NIMA (never in mitosis gene a)-related kinase 4	620,693	48	Inhibition (2/3)	Nontoxic	Viral helper*¶
NEK7	NIMA (never in mitosis gene a)-related kinase 7	488,763	38	Inhibition (2/3)	Nontoxic	Viral helper
NUDT8	Nudix (nucleoside diphosphate linked moiety X)-type motif 8	686,379	53	Inhibition (2/3)	Nontoxic	Viral helper
PAK4	p21 protein (Cdc42/Rac)-activated kinase 4	666,077	51	Inhibition (2/3)	Nontoxic	Viral helper
PAK6	p21 protein (Cdc42/Rac)-activated kinase 6	318,371	24	Inhibition (2/3)	Nontoxic	Viral helper¶
PDK4	Pyruvate dehydrogenase kinase, isozyme 4	404,650	31	Inhibition (2/3)	Nontoxic	Viral helper¶
PFKFB1	6-Phosphofructo-2-kinase/fructose-2,6-biphosphatase 1	569,142	44	Inhibition (2/3)	Nontoxic	Viral helper¶
PFKFB3	6-Phosphofructo-2-kinase/fructose-2,6-biphosphatase 3	655,851	50	Inhibition (2/3)	Nontoxic	Viral helper
PIK3C2G	Phosphoinositide-3-kinase, class 2, gamma polypeptide	778,879	60	Inhibition (2/3)	Nontoxic	Viral helper#¶
PPAP2B	Phosphatidic acid phosphatase type 2B	688,169	53	Inhibition (2/3)	Nontoxic	Viral helper
PPM1A	Protein phosphatase 1A (formerly 2C), magnesium-dependent, alpha isoform	716,657	55	Inhibition (2/3)	Nontoxic	Viral helper¶
PRKD3	Protein kinase D3	614,158	47	Inhibition (2/3)	Nontoxic	Viral helper¶
PRPF4B	PRP4 pre-mRNA processing factor 4 homolog B	710,584	55	Inhibition (2/3)	Nontoxic	Viral helper#¶
PTK7	PTK7 protein tyrosine kinase 7	605,684	47	Inhibition (2/3)	Nontoxic	Viral helper
PTP4A1	Protein tyrosine phosphatase type IVA, member 1	694,784	53	Inhibition (2/3)	Nontoxic	Viral helper
PTPRH	Protein tyrosine phosphatase, receptor type, H	438,851	34	Inhibition (2/3)	Nontoxic	Viral helper¶
PXK	PX domain containing serine/threonine kinase	431,209	33	Inhibition (2/3)	Nontoxic	Viral helper¶
RIOK2	RIO kinase 2 (yeast)	450,541	35	Inhibition (2/3)	Nontoxic	Viral helper¶
RWDD2B	RWD domain containing 2B	704,506	54	Inhibition (2/3)	Nontoxic	Viral helper¶
SRC	v-src sarcoma (Schmidt-Ruppin A-2) viral oncogene homolog	658,896	51	Inhibition (2/3)	Nontoxic	Viral helper
STK17B	Serine/threonine kinase 17b	506,617	39	Inhibition (2/3)	Nontoxic	Viral helper
STK38	Serine/threonine kinase 38	555,822	43	Inhibition (2/3)	Nontoxic	Viral helper
TNK1	Tyrosine kinase, nonreceptor, 1	617,576	48	Inhibition (2/3)	Nontoxic	Viral helper
TNK2	Tyrosine kinase, nonreceptor, 2	469,499	36	Inhibition (2/3)	Nontoxic	Viral helper
TRIB2	Tribbles homolog 2	500,927	39	Inhibition (2/3)	Nontoxic	Viral helper¶
TSKS	Testis-specific serine kinase substrate	384,024	30	Inhibition (2/3)	Nontoxic	Viral helper¶
TWF2	Twinfilin, actin-binding protein, homolog 2	726,794	56	Inhibition (2/3)	Nontoxic	Viral helper
UCK1	Uridine-cytidine kinase 1	697,419	54	Inhibition (2/3)	Nontoxic	Viral helper¶
PDGFRL	Platelet-derived growth factor receptor-like	2205,658	170	Activation (2/3)	Nontoxic	Viral inhibitor
PKN2	Protein kinase N2	2,288,330	176	Activation (2/3)	Nontoxic	Viral inhibitor
PPAP2A	Phosphatidic acid phosphatase type 2A	2,294,643	177	Activation (2/3)	Nontoxic	Viral inhibitor

a For the complete list of high-confidence hits identified, see Table S1. ¶, hits identified at both 18 and 36 h; *, hits retrospectively marked as validated; #, hits retrospectively marked as representing OTE.

**TABLE 2:** List of high-confidence hits identified after 36 h of RABV infection^*a*^

Gene designation	Full gene name	GFP score		Effect on virus (no. showing effect/total no.)	Effect on cells	Role
		Avg	%			
DUSP5	Dual-specificity phosphatase 5	1,443,234	32	Inhibition (3/3)	Nontoxic	Viral helper*¶
PRPF4B	PRP4 pre-mRNA processing factor 4 homolog B	1,927,450	43	Inhibition (3/3)	Nontoxic	Viral helper¶#
ANP32E	Acidic (leucine-rich) nuclear phosphoprotein 32 family, member E	2,135,627	47	Inhibition (2/3)	Nontoxic	Viral helper
ASB10	Ankyrin repeat and SOCS box-containing 10	1,571,835	35	Inhibition (2/3)	Nontoxic	Viral helper
ASNA1	*arsA* arsenite transporter, ATP-binding, homolog 1	778,338	17	Inhibition (2/3)	Nontoxic	Viral helper
ATP6V0E2	ATPase, H^+^ transporting V0 subunit e2	2,221,918	49	Inhibition (2/3)	Nontoxic	Viral helper
BUB1B	Budding uninhibited by benzimidazoles 1 homolog beta	1,927,314	43	Inhibition (2/3)	Nontoxic	Viral helper¶
CAMK2A	Calcium/calmodulin-dependent protein kinase II alpha	2,722,201	60	Inhibition (2/3)	Nontoxic	Viral helper
CCDC155	Coiled-coil domain containing 155	1,925,510	43	Inhibition (2/3)	Nontoxic	Viral helper
CDC25C	Cell division cycle 25 homolog C	2,855,149	63	Inhibition (2/3)	Nontoxic	Viral helper¶
CDC2L5	Cell division cycle 2-like 5	1,860,192	41	Inhibition (2/3)	Nontoxic	Viral helper
CDC42BPG	CDC42 binding protein kinase gamma (DMPK-like)	1,468,990	33	Inhibition (2/3)	Nontoxic	Viral helper
CDK5	Cyclin-dependent kinase 5	2,742,954	61	Inhibition (2/3)	Nontoxic	Viral helper
DAPK2	Death-associated protein kinase 2	1,757,053	39	Inhibition (2/3)	Nontoxic	Viral helper
EPHB2	EPH receptor B2	1,632,847	36	Inhibition (2/3)	Nontoxic	Viral helper¶
ERN1	Endoplasmic reticulum to nucleus signaling 1	2,656,404	59	Inhibition (2/3)	Nontoxic	Viral helper
FBP2	Fructose-1,6-bisphosphatase 2	1,264,888	28	Inhibition (2/3)	Nontoxic	Viral helper
FGR	Gardner-Rasheed feline sarcoma viral (v-fgr) oncogene homolog	424,483	9	Inhibition (2/3)	Nontoxic	Viral helper¶
FUK	Fucokinase	2,781,274	62	Inhibition (2/3)	Nontoxic	Viral helper
GLYCTK	Glycerate kinase	2,279,568	51	Inhibition (2/3)	Nontoxic	Viral helper
IMPAD1	Inositol monophosphatase domain containing 1	2,486,915	55	Inhibition (2/3)	Nontoxic	Viral helper
INPP1	Inositol polyphosphate-1-phosphatase	1,903,394	42	Inhibition (2/3)	Nontoxic	Viral helper
INPP5E	inositol polyphosphate-5-phosphatase, 72 kDa	1,745,212	39	Inhibition (2/3)	Nontoxic	Viral helper¶
ITPKB	Inositol 1,4,5-trisphosphate 3-kinase B	1,359,158	30	Inhibition (2/3)	Nontoxic	Viral helper
KHK	Ketohexokinase	1,536,443	34	Inhibition (2/3)	Nontoxic	Viral helper
LATS2	LATS, large tumor suppressor, homolog 2	1,903,478	42	Inhibition (2/3)	Nontoxic	Viral helper
MAGI2	Membrane-associated guanylate kinase, WW and PDZ domain containing 2	1,919,881	43	Inhibition (2/3)	Nontoxic	Viral helper¶
MAP2K7	Mitogen-activated protein kinase kinase 7	2,279,213	51	Inhibition (2/3)	Nontoxic	Viral helper*
MAP3K14	Mitogen-activated protein kinase kinase kinase 14	1,912,313	42	Inhibition (2/3)	Nontoxic	Viral helper
MATK	Megakaryocyte-associated tyrosine kinase	1,893,939	42	Inhibition (2/3)	Nontoxic	Viral helper
MINPP1	Multiple inositol polyphosphate histidine phosphatase 1	1,865,987	41	Inhibition (2/3)	Nontoxic	Viral helper*¶
MPP7	Membrane protein, palmitoylated 7	1,873,155	42	Inhibition (2/3)	Nontoxic	Viral helper
MST1R	Macrophage-stimulating 1 receptor	2,384,937	53	Inhibition (2/3)	Nontoxic	Viral helper
NEK4	NIMA (never in mitosis gene a)-related kinase 4	2,757,646	61	Inhibition (2/3)	Nontoxic	Viral helper*¶
NRBP2	Nuclear receptor binding protein 2	2,268,354	50	Inhibition (2/3)	Nontoxic	Viral helper
PAK6	p21 protein (Cdc42/Rac)-activated kinase 6	589,450	13	Inhibition (2/3)	Nontoxic	Viral helper¶
PDK4	Pyruvate dehydrogenase kinase, isozyme 4	251,114	6	Inhibition (2/3)	Nontoxic	Viral helper¶
PFKFB1	6-Phosphofructo-2-kinase/fructose-2,6-biphosphatase 1	1,005,379	22	Inhibition (2/3)	Nontoxic	Viral helper¶
PIK3C2G	Phosphoinositide-3-kinase, class 2, gamma polypeptide	2,555,584	57	Inhibition (2/3)	Nontoxic	Viral helper#¶
PIP5K1C	Phosphatidylinositol-4-phosphate 5-kinase, type I, gamma	1,933,840	43	Inhibition (2/3)	Nontoxic	Viral helper*
PKLR	Pyruvate kinase, liver and RBC	2,146,746	48	Inhibition (2/3)	Nontoxic	Viral helper
PLK4	Polo-like kinase 4	1,663,736	37	Inhibition (2/3)	Nontoxic	Viral helper
PLXNB2	Plexin B2	2,088,260	46	Inhibition (2/3)	Nontoxic	Viral helper
PPM1A	Protein phosphatase 1A (formerly 2C), magnesium dependent, alpha isoform	1,974,978	44	Inhibition (2/3)	Nontoxic	Viral helper¶
PPP1R12C	Protein phosphatase 1, regulatory subunit 12C	2,324,349	52	Inhibition (2/3)	Nontoxic	Viral helper
PPP1R14D	Protein phosphatase 1, regulatory (inhibitor) subunit 14D	2,225,583	49	Inhibition (2/3)	Nontoxic	Viral helper
PPP1R2	Protein phosphatase 1, regulatory (inhibitor) subunit 2	2,090,356	46	Inhibition (2/3)	Nontoxic	Viral helper
PPP1R7	Protein phosphatase 1, regulatory subunit 7	3,020,584	67	Inhibition (2/3)	Nontoxic	Viral helper
PPP2CA	Protein phosphatase 2, catalytic subunit, alpha isozyme	1,377,381	31	Inhibition (2/3)	Nontoxic	Viral helper¶
PRKD3	Protein kinase D3	2,188,261	49	Inhibition (2/3)	Nontoxic	Viral helper¶
PTPRH	Protein tyrosine phosphatase, receptor type, H	2,102,127	47	Inhibition (2/3)	Nontoxic	Viral helper¶
PXK	PX domain containing serine/threonine kinase	211,663	5	Inhibition (2/3)	Nontoxic	Viral helper¶
RIOK2	RIO kinase 2	1,472,591	33	Inhibition (2/3)	Nontoxic	Viral helper¶
RNGTT	RNA guanylyltransferase and 5'-phosphatase	526,461	12	Inhibition (2/3)	Nontoxic	Viral helper#
RPS6KA2	Ribosomal protein S6 kinase, 90 kDa, polypeptide 2	1,700,318	38	Inhibition (2/3)	Nontoxic	Viral helper
RPS6KA5	Ribosomal protein S6 kinase, 90 kDa, polypeptide 5	689,086	15	Inhibition (2/3)	Nontoxic	Viral helper#
RWDD2B	RWD domain-containing 2B	2,228,746	50	Inhibition (2/3)	Nontoxic	Viral helper¶
SCYL1	SCY1-like 1	1,593,150	35	Inhibition (2/3)	Nontoxic	Viral helper
STRADB	STE20-related kinase adaptor beta	2,340,319	52	Inhibition (2/3)	Nontoxic	Viral helper
TLK1	Tousled-like kinase 1	1,844,202	41	Inhibition (2/3)	Nontoxic	Viral helper
TRIB2	Tribbles homolog 2	952,306	21	Inhibition (2/3)	Nontoxic	Viral helper¶
TSKS	Testis-specific serine kinase substrate	986,981	22	Inhibition (2/3)	Nontoxic	Viral helper¶
TTBK2	tau tubulin kinase 2	2,866,957	64	Inhibition (2/3)	Nontoxic	Viral helper
TTK	TTK protein kinase	1,239,442	28	Inhibition (2/3)	Nontoxic	Viral helper
UCK1	Uridine-cytidine kinase 1	1,559,340	35	Inhibition (2/3)	Nontoxic	Viral helper¶
WNK2	WNK lysine-deficient protein kinase 2	1,797,141	40	Inhibition (2/3)	Nontoxic	Viral helper
FYN	FYN oncogene related to SRC, FGR, YES	7,441,366	165	Activation (2/3)	Nontoxic	Viral inhibitor#
MTM1	Myotubularin 1	7,718,965	172	Activation (2/3)	Nontoxic	Viral inhibitor*
PPP1CC	Protein phosphatase 1, catalytic subunit, gamma isoform	7,759,759	172	Activation (2/3)	Nontoxic	Viral inhibitor
PTPN1	Protein tyrosine phosphatase, nonreceptor type 1	7,532,909	167	Activation (2/3)	Nontoxic	Viral inhibitor
PTPN2	Protein tyrosine phosphatase, nonreceptor type 2	7,805,315	173	Activation (2/3)	Nontoxic	Viral inhibitor
PTPN7	Protein tyrosine phosphatase, nonreceptor type 7	7,681,547	171	Activation (2/3)	Nontoxic	Viral inhibitor
PTPN9	Protein tyrosine phosphatase, nonreceptor type 9	7,620,918	169	Activation (2/3)	Nontoxic	Viral inhibitor

a For the complete list of high-confidence hits identified, see Table S2. ¶, hits identified at both 18 and 36 h; *, hits retrospectively marked as validated; #, hits retrospectively marked as representing OTE. RBC, red blood cells.

10.1128/mSphere.00047-19.7 TABLE S1 Complete list of hits identified after 18 h of RABV infection. Download Table S1, DOCX file, 0.3 MB. Copyright © 2019 Besson et al. 2019 Besson et al. This content is distributed under the terms of the Creative Commons Attribution 4.0 International license.

10.1128/mSphere.00047-19.8 TABLE S2 Complete list of hits identified after 36 h of RABV infection. Download Table S2, DOCX file, 0.3 MB. Copyright © 2019 Besson et al. 2019 Besson et al. This content is distributed under the terms of the Creative Commons Attribution 4.0 International license.

### Identifying key functions and pathways involved in RABV infection.

**FIG 2:**
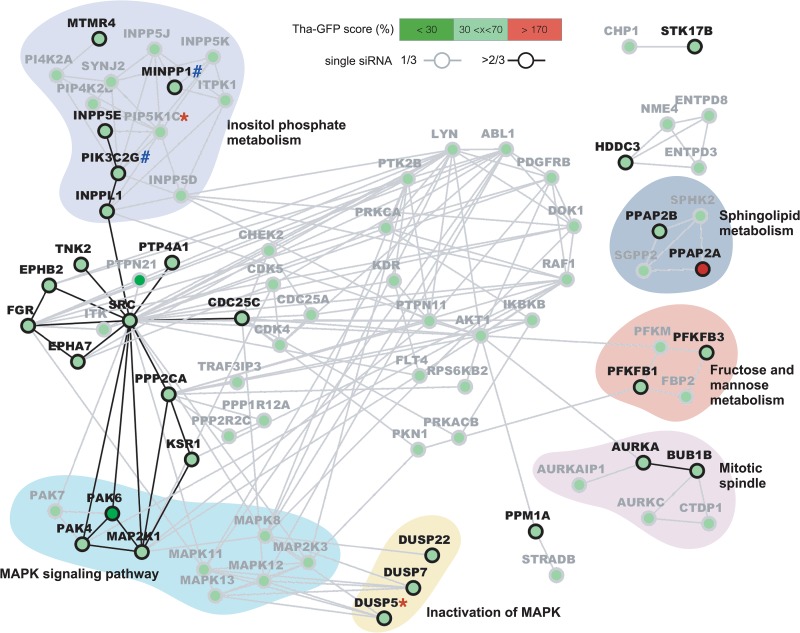
Protein network analysis of hits affecting RABV infection after 18 h. High-confidence (≥2 siRNA, black) and low-confidence (1 siRNA, gray) hit interactions were computed using STRING v10.5 and were visualized using Cytoscape v3.4. Only low-confidence hits directly interacting with at least one high-confidence hit are displayed. The Tha-GFP score corresponds to the average GFP signal measured in each active single siRNA normalized to the mean of the data from the siNEG control. Hits were retrospectively marked as validated (*) or OTE (#).

**FIG 3:**
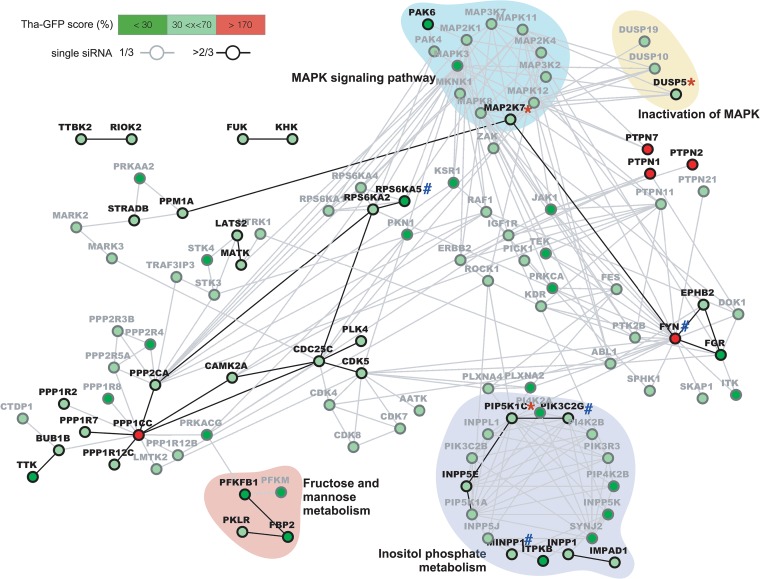
Protein network analysis of hits affecting RABV infection after 36 h. High-confidence (≥2 siRNA, black) and low-confidence (1 siRNA, gray) hit interactions were computed using STRING v10.5 and were visualized using Cytoscape v3.4. Only low-confidence hits directly interacting with at least one high-confidence hit are displayed. The Tha-GFP score corresponds to the average GFP signal measured in each active single siRNA normalized to the mean of the data from the siNEG control. Hits were retrospectively marked as validated (*) or OTE (#).

In order to identify the main functions and pathways that are taken advantage of during RABV infection, we next used STRING to form a network of genes identified at 18 h (Fig. 2) and 36 h (Fig. 3) postinfection. For this purpose, we computed high-confidence hits and low confidence hits, but none of the hits were labeled toxic. Further, only low-confidence hits showing a direct interaction with a high-confidence hit(s) were displayed; where possible, biological functions were attributed to gene clusters by the use of DAVID.

At the early stages of the infection, two metabolic pathways were identified as key host factors (Fig. 2). Among 4 hits involved in sphingolipid metabolism, 2 lipid phosphate phosphohydrolases (PPAP2A and PPAP2B) were defined as a high-confidence viral inhibitor and a viral helper, respectively. Fructose metabolism and mannose metabolism were also identified as important for RABV replication as, in a cluster of 4 genes, 2 phosphofructokinase-biphosphatases (PFKBP1 and PFKBP3) were high-confidence viral helpers. Two genes encoding mitotic checkpoint kinases (AURKA and BUB1B) in a cluster of 5 genes involved in the mitotic spindle were high-confidence viral helper genes (Fig. 2). Interestingly, numerous other hits defined as viral helpers are involved in mitotic regulation. In particular, CDC25C was shown to be important at both 18 h and 36 h postinfection and all three single siRNAs used against it were shown to affect RABV replication. Further, a significant number of MAPK (MEK1/MAP2K1)-associated phosphatases (DUSP5, DUSP7, and DUSP22) and downstream effectors (PAK4 and PAK6) were defined as high-confidence viral helpers among a total of 12 MAPK-associated proteins (Fig. 2). Interestingly, PAK6 interference was the most effective with respect to RABV replication at 18 h, reducing the Tha-GFP signal to 24% of levels seen with the controls (Table 1). The largest cluster of genes involved as viral helpers of RABV corresponded to inositol phosphate metabolism, with 5 high-confidence hits (INPPL1, INPP5E, MINPP1, MTMR4, and PIK3C2G) among 13 hits (Fig. 2). This result was linked to two downstream effectors of phosphatidylinositol (PI) signaling, AKT1 and AKT2, both of which had 1 siRNA that was effective at 18 h as well as 36 h for AKT2. Finally, it is striking that in Fig. 2, the gene coding for the proto-oncogene tyrosine protein kinase Src forms a hub among several high-confidence viral helpers (CDC25C, EPHBA7, EPHB2, FGR, PPP2CA, PTP4A1, and TNK2) as well as connecting with the previously described MAPK and inositol phosphate pathways. It is also noteworthy that SRC interference had an inhibitory effect on Tha-GFP at 36 h. However, it was not considered for further analysis as it was also shown to be cytotoxic (Table S2).

At the later stages of the infection, only four gene clusters were associated with a function or pathway (Fig. 3), and all were already identified at the early stages (Fig. 2). First, mannose metabolism and fructose metabolism were also shown to be important in the later stages, as a phosphofructokinase-biphosphatase (PFKBP2), a fructose-biphosphatase (FBP2), and pyruvate kinase (PKLR) were high-confidence viral helpers. If we consider the hits labeled “low confidence,” FBP2 had already been identified at 18 h and the phosphofructokinase PFKM was identified at both 18 h and 36 h (Fig. 2). Interestingly, mannose and fructose metabolism interference reduced Tha-GFP signal by 50% at 18 h and by more than 70% at 36 h (Table 2; see also Table S2). In addition, high-confidence MAPK (MKK7/MAP2K7) and a downstream effector (PAK6) as well as an associated phosphatase (DUSP5) were again identified as viral helpers at 36 h (Fig. 2). If we consider the hits labeled “low confidence,” PAK4 and MAP2K1 (MEK1) were identified with only one siRNA after 18 h (Fig. 2; see also Table S2). At 36 h, other high-confidence MAPK regulators such as RSK (RPS6KA2 and RPS6KA5) and protein tyrosine-phosphatase nonreceptor (PTPN7) as well as many MAPK regulators labeled low confidence (i.e., RPS6KA1, RPS6KA4, ZAK, JAK1, and KSR1) were identified as viral helpers. PAK6 interference was also strongly affecting RABV replication, reducing Tha-GFP signal by 87%, as well as RPS6KA5 (RSK) interference, reducing Tha-GFP signal by 85% (Table 2). It should be noted that JNK/MAPK8 was identified as a viral helper with only 1 siRNA at both 18 and 36 h while extracellular signal-regulated kinase (ERK)/MAPK1 silencing was associated with cell toxicity (Table S1 and S2). Finally, genes associated with inositol phosphate metabolism also constituted the largest cluster observed at 36 h with 7 high-confidence hits (IMPAD1, NPP1, INPP5E, ITPKB, MINPP1, PIK3C2G, and PIP5K1C) among 17 hits (Fig. 3). Among them, ITPKB interference reduced Tha-GFP signal by 70% whereas the other genes were associated with a 50% to 60% decrease of Tha-GFP signal (Table 2).

**FIG 4:**
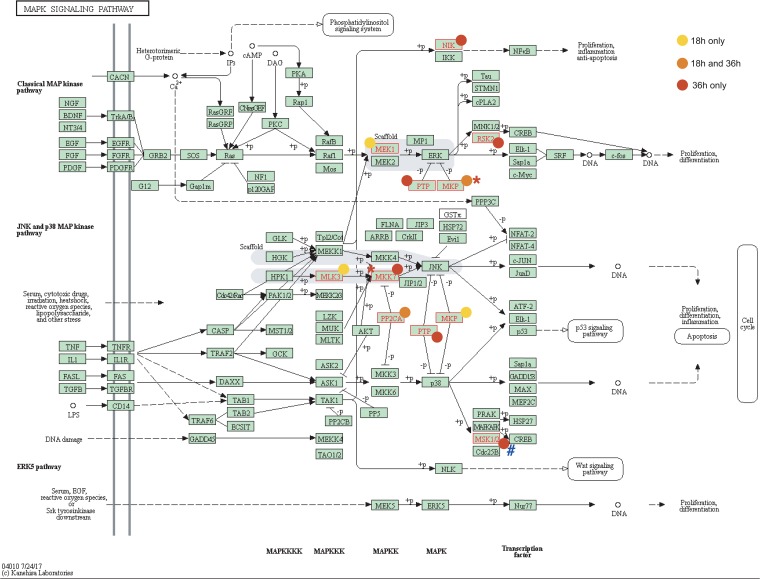
Mapping of hits in the MAPK signaling pathway. Hits that affected RABV at 18 h (yellow) or 36 h (red) or both (orange) are indicated (see link on KEGG database). Displayed proteins correspond to identified genes as follows: NIK = MAP3K14, MEK1 = MAP2K1, RSK2 = RPS6KA2, PTP = PTPN7/9, MKP = DUSP5/7/9, MLK3 = MAP3K11, MKK7 = MAP2K7, PP2CA = PPM1A, and MSK1/2 = RPS6KA5. Hits were retrospectively marked as validated (*) or OTE (#). (Reprinted from KEGG Pathway Maps with permission of the publisher.)

**FIG 5:**
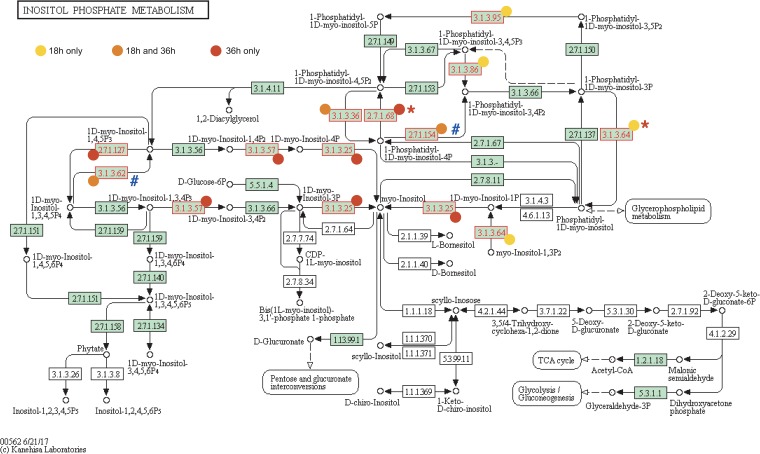
Mapping of hits in the inositol phosphate metabolism. Hits that affected RABV at 18 h (yellow) or 36 h (red) or both (orange) are indicated (see link on KEGG database). Displayed enzymatic functions correspond to identified genes as follows: 2.7.1.68 = PIP5K1C, 2.7.1.154 = PI3KC2G, 2.1.1.127 = ITPKB, 3.1.3.25 = IMPAD1, 3.1.3.36 = INPP5E and MTMR4, 3.1.3.57 = INPP1, 3.1.3.62 = MINPP1, 3.1.3.64 = MTM1 and MTMR4, 3.1.3.86 = INPPL1, and 3.1.3.95 = MTM1. Hits were retrospectively marked as validated (*) or OTE (#). (Reprinted from KEGG Pathway Maps with permission of the publisher.)

To provide a comprehensive overview of the most prominent pathways identified as key factors for RABV infection cycle, all the high-confidence hits for both the early and late infection stages were mapped on the MAPK signaling (Fig. 4), PI metabolism (Fig. 5), PI signaling system (Fig. S3), and mannose and fructose metabolism (Fig. S4) pathways using KEGG Pathways.

### Confirmation of key host factors.

**FIG 6:**
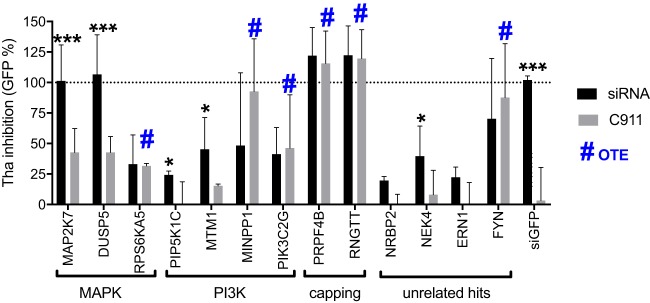
Confirmation of specific silencing of RABV. HEK293T cells were transfected with a single siRNA at 25 nM and infected 72 h later with Tha-GFP at an MOI of 5. After 36 h, Hoechst and GFP signals were acquired. Tha-GFP inhibition was determined by comparisons between the negative control (siNEG; 0% inhibition) and the positive control (siGFP; 100% inhibition). GFP% = 100 − [100 × (siHIT − siGFP)/(siCTRL − siGFP)]. Each value represents the mean of data from 3 independent experiments, and error bars represent standard deviations. The statistical significance of results of comparisons of the original siRNA to the corresponding C911 counterpart was determined using ANOVA in Prism 7 (GraphPad). ***, *P* < 0.001; *, *P* < 0.05.

First, we investigated a selection of high-confidence hits using the C911 method (33) to determine whether the inhibition of RABV could have been due to off-target effects (OTE) or if it was specific to the siRNA/target identified (Table S2). Briefly, the C911 method is based on the design of mismatch siRNAs that carry OTE but lose on-target effects. We selected a single siRNA for high-confidence hits that are present at different levels in the MAPK pathway (MKK7/MAP2K7, DUSP5, and RPS6KA5) or the PI3K pathway (PIP5K1C, MTM1, MINPP1, and PIK3C2G) or that are involved in mRNA capping (PRPF4B and RNGTT) or are associated with various other cellular functions (NRBP2, NEK4, ERN1, and FYN). As the C911-modified siRNA should not affect the targets, we were able to demonstrate that the following hits were clearly subject to OTE: RPS6KA5, MINPP1, PIK3C2G, PRPF4B, RNGTT, and FYN (Fig. 6). Both siMAP2K7 (MKK7) and siDUSP5 of the MAPK pathway reached 100% inhibition compared to siGFP (Fig. 6), a level significantly higher than that seen with their C911 target (*P* < 0.001). Both siPIP5K1C and siMTM1 of the phosphatidylinositol 3-phosphate (PI3P) pathway were seen to inhibit Tha-GFP compared to their C911 target (*P* < 0.05), providing 25% and 45% inhibition, respectively. Furthermore, NEK4 but not its C911 target (*P* < 0.05) inhibited 39% of Tha-GFP replication and both NRBP2 and ERN1 inhibited ∼20% of Tha-GFP but the levels were not statistically significantly different from those seen with their C911 target (*P* = 0.054 and *P* = 0.103, respectively). NEK4, involved in replicative senescence, has never been found to be associated with host-virus interactions (34). This confirmed that the hits identified in the RNAi screening—in particular, the hits from both the MAPK and phosphatydilinositol-3-phosphate (PI3P) pathways—are important for RABV replication.

The importance of the most prominent pathways was assessed with a small-scale compound screening experiment based on MAPK and PI3P pathway inhibitors cherry-picked in our compound libraries (Sigma and Selleckchem). In total, 10 MAPK inhibitors and 4 PI3P inhibitors (Table S4) were tested using 10 concentration points ranging from 10 μM to 5 nM.

The compound screening was first run on HEK293T cells infected with Tha-GFP to validate the system used for the RNAi screening. At 18 h (Fig. S5A), we were able to fit dose-response curves to 8 inhibitors (Ro 31-8220 mesylate, D-609, idelalisib, NSC663284, PD98059, SD169, SL327, and wortmannin), among which 1 (Ro 31-8220 mesylate) was cytotoxic and 2 (NSC663284 and SD169) showed a trend toward significance but gave results that were above the level seen with the control (DMSO [dimethyl sulfoxide]). At 36 h (Fig. S5B), only PD198603 and Ro 31-8220 mesylate data were fitted to a dose-response curve. As expected, Ro 31-8220 mesylate appeared to be cytotoxic in HEK293T cells. PD198306 was significantly inhibiting Tha-GFP replication by 25% at the highest concentration tested, 10 μM, after 36 h (GFP intensity of 3.10^7^ opposed to 4.10^7^ in DMSO).

**FIG 7:**
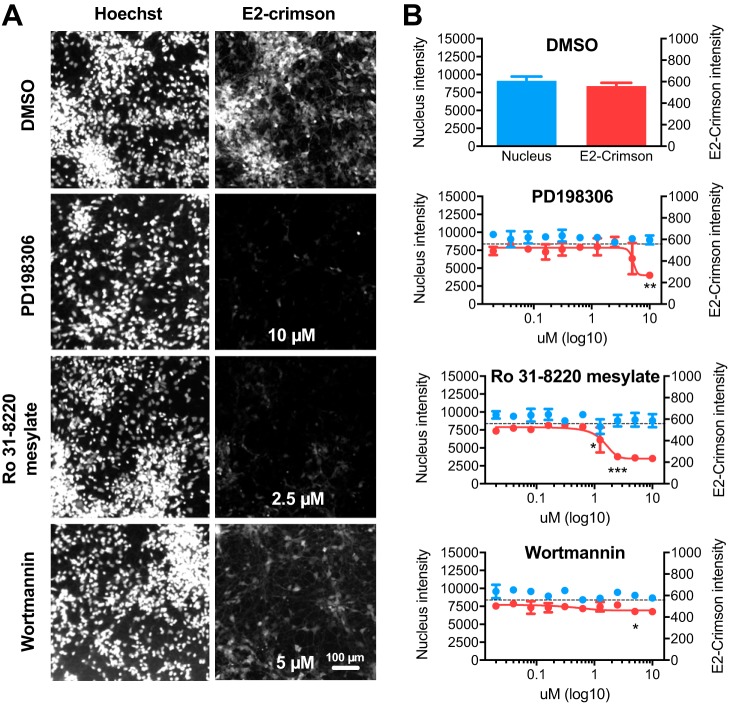
Inhibition of RABV infection by Ro-31-8220 mesylate and PD 198306 in human neurons. Human iPSCs were infected with Tha-Crimson at an MOI of 3 after 6 h of treatment with DMSO (0.5%), PD 198306, Ro-31-8220 mesylate, or wortmannin. Cells were fixed with 4% PFA at 36 h postinfection. Images were acquired (A) and analyzed (B) to determine the relative numbers of nuclei (Nucleus intensity) and the relative quantities of Tha-Crimson (E2-Crimson intensity). The data corresponding to the latter were fitted using nonlinear regression. Each value represents the mean of data from 2 separate wells, and error bars represent standard deviations. Data corresponding to differences between the results seen with the compounds and the results seen with DMSO were assessed using ANOVA in Prism 7 (GraphPad). The *P* values and images are annotated for the lowest dose with a significant effect. ***, *P* < 0.001; **, *P* < 0.01; *, *P* < 0.05. Dotted line = average Tha-Crimson value in DMSO.

To confirm our hits in a more relevant model for RABV infection, we treated human neurons differentiated from induced pluripotent stem cells (IPSCs) with the selected compounds. Using a newly generated Tha-Crimson recombinant virus that provides a better signal/noise ratio in neurons than Tha-GFP, we observed consistent results 18 h (Fig. S6A) and 36 h (Fig. S6B) postinfection. Among 14 compounds, one (NSC663284) was cytotoxic at concentrations above 5 μM (Fig. S6A and S6B) and 3 compounds had an inhibitory effect on Tha-Crimson at 18 h (Fig. S6A) and 36 h (Fig. 7; see also Fig. S6B). PD198306 inhibited Tha-Crimson replication at 10 μM (Fig. 7A), reducing it by 30% at 18 h (Fig. S6A) and 50% at 36 h (Fig. 7). Furthermore, PD198306 5 μM reduced Tha-Crimson replication significantly by 20% at 18 h (Fig. S6A) but such a result could not be confirmed at 36 h. In addition, Ro 31-8220 mesylate at 1.25 μM inhibited Tha-Crimson replication significantly by 12% at 18 h (Fig. S6A) and by 28% at 36 h (Fig. 7). At 2.5 μM, Ro 31-8220 mesylate inhibited Tha-Crimson replication by 23% at 18 h (Fig. S6A) and by 55% at 36 h (Fig. 7). Finally, wortmannin inhibited Tha-Crimson replication by 10% from 2.5 μM at 18 h (Fig. S6A) and by 20% from 5 μM at 36 h (Fig. 7).

Several inhibitors of MAPKs such as p38 (BIRB 796, SD169), MAPK kinases (MAPKKs) such as MEK1/2 (PD 98059, U0126 and 2d), phosphatases (NSC 663284 and Me-3,4-dephostatin), and inositol metabolism (D-609 potassium, lithium chloride, idelalisib) had no apparent effect on RABV replication. SL327, which inhibits MEK1/2, induced only weak inhibition of RABV at 18 h in HEK293T. Wortmannin, which targets phosphatidyl-inositol metabolism (including PI3K, PI4K, DNA protein kinase C [PKC], and ATR metabolism), had a small but consistent effect on RABV. PD 198306 had an important effect only at a high concentration, suggesting that it could be dependent on the presence of secondary targets, including ERK and PI3K. The lack of cytotoxicity of PD 198306 suggests that it is not related to SRC (Table S1 and S2). As several cyclin-dependent kinases (CDKs) were shown to be involved in RABV replication (Table S1 and S2), it is possible that CDKs mediate PD 198306 inhibition of RABV. Ro 31-8220 mesylate, which targets PKC and MAPK effectors, was the most effective inhibitor of RABV. It should be noted that in the siRNA screening (Table S1 and S2), only 1/3 siRNAs targeting PKC subunits (PRKCA, PRKCDBP, and PRKCG) had an effect of RABV. The strong effect of Ro 31-8220 mesylate in combination with the siRNA suggests that PKC could mediate the Ro 31-8220 mesylate-dependent inhibition of RABV infection.

Due to the broad range of targets of kinase inhibitors (Table S3) compared to the high specificity of siRNA and the differences between an immortalized cell line such as the HEK293T cell line and differentiated neurons, it is difficult to reach a conclusion on the individual role of each target. However, 3 compounds targeting MAPKs and effectors, phosphatidyl-inositol metabolism, and other targets had a consistent and significant inhibitory effect on Tha virus replication at both 18 h and 36 h postinfection in human neurons.

10.1128/mSphere.00047-19.9 TABLE S3 List of siRNA and C911 targets. Download Table S3, DOCX file, 0.06 MB. Copyright © 2019 Besson et al. 2019 Besson et al. This content is distributed under the terms of the Creative Commons Attribution 4.0 International license.

## DISCUSSION

Representing only 2% of the genome, protein kinases regulate the structures and functions of 30% of all cellular proteins and are involved in a wide spectrum of cellular processes (35). As viruses such as RABV exploit many assets of the cellular machinery to their own benefit throughout their infectious cycle, the host kinome represents a target of choice to identify various key host factors in a large-scale RNAi screening.

Several host factors for RABV had already been identified previously in individual studies, including Hsc70 (36), CCTα (37), and NCL (32). The latter was shown to be important for viral protein expression and infectious virus production after 24 h of infection. According to our results, NCL is indeed important, although not essential, for viral replication at the early stages of the infection; however, it is not required at the later stages (see Fig. S2 in the supplemental material). In a large-scale loss-of-function screening of murine differentiated neurons (29), the following 2 kinases among 63 hits were involved in later stages of RABV infection: Pick1 (PRKCA-binding protein) and FN3KRP (fructosamine 3 kinase-related protein). In our assay, only Pick1 was found to be a viral helper at both early and later stages of RABV infection with a single siRNA. As a protein adaptor for PDZ domain proteins playing a role in synapse trafficking, Pick1 could therefore be involved in the interaction of G protein with PDZ domain proteins MAST1/2 and PTPN4 in the control of cell survival (10). Further, several PTPN proteins were identified as viral inhibitors in the later stages and that effect could be linked to the capacity of the G protein to interact with PTPN4. Finally, the proteins identified as involved in mitotic regulation (AURKA and BUB1B) are notably important for microtubule regulation and could have an impact on rabies virus transport (38).

In the light of the different proteins and pathways known to be directly affected by RABV, the present screening provided new insights. It should be noted that although HEK293T cells lack Toll-like receptors (39), they have functioning RLRs and downstream pathways, and they are able to build up an IFN response (40–42). The silencing of TPL2 and TBK1, both of which are linked to RABV escape of the immune system (18, 19), had no effect on RABV replication. MAP2K6/MEK6, whose expression was shown to be repressed by Tha virus (15), had no significant impact on viral replication. MAP3K14, or NIK, which is crucial for the induction of the noncanonical NF-κB pathway (43), is essential for RABV replication at later stages, while the canonical pathway was shown to be targeted by the M protein to escape the immune response (14, 15, 19). This indicates that, unlike the canonical pathway, which is inhibited during RABV infection, the noncanonical NF-κB pathway is important for RABV replication and the effect could be linked to its disturbance by M-RelAp43 interaction. Indeed, in a previous mass spectrometry analysis of RelAp43 interacting proteins, we found that the M-protein strikingly increases RelB and p100/p52 interaction with RelAp43 (19). Finally, if we consider a less stringent 2σ threshold for the selection of viral helpers, all proteins of the Janus kinase family (JAK1/2/3 and TYK2) had at least 1 siRNA effective at 18 h postinfection. This suggests a role in RABV replication of this protein family triggering the JAK/STAT pathway, inhibited downstream of JAK proteins by the P protein of RABV (16, 17), as well as in regulating MAPK (44).

The MAPK pathway is involved in many physiological functions such as cellular growth, differentiation, and survival as well as neuronal functions and the immune response (45). Here, the presence of several MAPK actors such as MEK1 and RSK2 of the ERK pathway and MLK3 and MKK7 of the JNK pathway as well as MAPK inhibitors (PTPN and DUSP phosphatases) appears to be highly important for RABV replication, suggesting the need of a finely tuned MAPK pathway to ensure the survival of infected cells without triggering the immune response. This correlates with the activation of the ERK/MAPK pathway previously observed in macrophages and microglia infected with RABV, triggering the production of CXCL10 and reactive nitrogen species (46, 47).

PI metabolism consist of a wide range of phosphorylated lipids involved in events of membrane fusion between cellular compartments (48) and cell signaling, centered around the PI3 kinases (49). If we have demonstrated here a role of PI metabolism in the RABV infectious cycle for the first time, it has been previously found to be associated with both entry and egress for several viruses. Phosphatydilinositol-3,5-biphosphate [PI(4,5)P_2_] is required for foot-and-mouth disease virus (FMDV; *Picornaviridae*) and VSV (*Rhabdoviridae*) internalization (50) as well as Ebola virus (EBOV; *Filoviridae*) egress (51), and PI(3)P was shown to affect influenza virus (*Orthomyxoviridae*) propagation (52). PI kinases PI3K and PI4K3 are also required for EBOV and severe acute respiratory syndrome coronavirus (SARS-CoV; *Coronaviridae*) entry, respectively (53, 54). Further, PI(4)P and PI4K3 are also essential for the formation of the viral replication complex of Aichi virus (*Picornaviridae*) in organelles (55, 56). In regard to closely related viruses such as VSV or EBOV, we can hypothesize that (i) several PI-related proteins playing an essential role at the early stages (MTM1, MTMR4, INPPL1, MINPP1, INPP5E, and PI3KC2G) are involved in RABV entry and that (ii) other PI-related proteins only affecting the later stages (IMPAD1, PIP5K1C, INPP1, and ITPKB) are involved in RABV egress. In particular, the PI-related proteins appear to all converge in the production of myo-inositol through I(3)P or I(4)P or I(3,4)P_2_ or some combination thereof in the later stages (Fig. 5).

Ro 31-8220 is a protein kinase C (PKC) inhibitor and is also active with respect to effects on several MAPKs or MAPK effectors (MAPKAP-K1b, MSK1, GSK3b, and S6K1). Interestingly, PKC has been previously shown to be important for the phosphorylation of RABV P protein (57). Therefore, in accordance with the high sensitivity of the virus to PKC inhibitor Ro 31-8220, the importance of this mechanism for RABV replication should be considered and further investigated. PD 198306 is a MEK1/2 inhibitor and is also active with respect to effects on other cellular kinases (ERK, c-Src, CDKs, and PI3-kinase). Finally, wortmannin primarily targets PI3K but also cross-reacts with other effectors of the phosphatidyl-inositol metabolism (DNA-PKCs, ATR, and PI4K). As many of the secondary targets of Ro 31–8820, PD 198306, and wortmannin were also identified in the RNAi screening as part of the MAPK pathway (MAPKAP-K1b, MSK1, GSK3b, and ERK), the PIP pathway (S6K1, PI3K, and PI4K), and other pathways (c-Src, CDKs), it is difficult to conclude which affects RABV replication, and the answer is likely to involve a combination of several targets. However, the results from this assay were consistent with the idea of MAPK and PI3K being important for RABV infection in differentiated human neurons.

Loss-of-function screenings using technologies such as RNA interference represent powerful approaches allowing systematic analysis of a system at a focused or genomic scale (58). Here, targeting the kinome provided an insightful view of the RABV infectious cycle, identifying new key host factors and pathways involved at different stages of the infection and supported by the literature. Further, specific compounds confirmed the importance of MAPK and PI3K in differentiated human neurons, a highly relevant model for the study of RABV, opening the way toward understanding the underlying mechanisms.

## MATERIALS AND METHODS

### Small molecules used in this study.

A list of the small molecules used in this study and their known targets according to the manufacturer is provided in Table S4 in the supplemental material.

10.1128/mSphere.00047-19.10 TABLE S4 List of small molecules used and their known targets according to the manufacturer. Download Table S4, DOCX file, 0.09 MB. Copyright © 2019 Besson et al. 2019 Besson et al. This content is distributed under the terms of the Creative Commons Attribution 4.0 International license.

### Cell culture.

The human embryonic kidney HEK293T cell line (HEK-293T/17; ATCC CRL-11268) was grown at 37°C in 5% CO_2_. HEK293T cells were propagated in complete growth medium containing Dulbecco’s modified Eagle’s medium supplemented with 10% heat-inactivated fetal bovine serum. All cell culture supplies were from Wellgene (Gyeongsan, South Korea) and Invitrogen (CA, USA).

The neural progenitor cell line (NPC) used in this study carries a neuron differentiation Map2-Nluc marker and was provided by Jihwan Song (CHA Stem Cell Institute, CHA University). NPCs were cultured at a 1:1 ratio of N2 and B27 media with 20 ng/ml of basic fibroblast growth factor (bFGF). N2 medium consisted of DMEM/F12 plus GlutaMAX, 1× N2, 1× nonessential amino acids, 5 μg/ml insulin (Sigma), 100 μM β-mercaptoethanol (Sigma), penicillin, and streptomycin. B27 medium consisted of Neurobasal medium, 1× B27 with or without vitamin A, 1× GlutaMAX, penicillin, and streptomycin (59). NPCs were maintained on a poly-l-ornithine/laminin-coated dish or plate. All materials for media were supplied by Gibco unless mentioned otherwise.

### Recombinant virus construction and reverse genetics.

A Tha-GFP recombinant virus was built based on the wild isolate Thailand RABV (isolate 8743 THA, EVAg collection, Ref-SKU: 014 V-02106). The complete sequence of Tha was cloned in Tha-pSDI-HH-flash-SC vector (Tha-rec) as previously described (15). Using the same approach, the E2-Crimson gene was inserted into the virus to obtain a Tha-Crimson virus. Fragment *F3-2* of the genome delimited by SanD1 and Sma1 (see Fig. S1A in the supplemental material) was subcloned in Topo-TA vector (Invitrogen). A PacI restriction site was created by mutagenesis in the M-G intergenic region by replacing 2 bases (Fig. S1A). The sequence encoding E2-Crimson was amplified from the pEGF-C1 vector (Invitrogen), with simultaneous addition of a short intergenic region that included the initiation and termination sequences of the M gene as indicated in Fig. S1A. The *F3-2* fragment was then replaced in Tha-pSDI-HH-flash-SC vector.

10.1128/mSphere.00047-19.1 FIG S1 Tha-GFP and Tha-Crimson construction and comparative kinetics. (A) Tha-GFP and Tha-Crimson were constructed by inserting a reporter eGFP or the E2-Crimson gene between the M and G genes and under the control of the same initiation and termination signal sequences as the M gene. The Tha-pSDI-HH-flash-SC vector (Tha-rec) (15) was used as a template, resulting in insertion of the new gene on the F3-2 fragment with SanDI and SmaI restriction enzymes. A PacI restriction site was created by directed mutagenesis of 2 bases (red). This allowed the insertion of a 65-base “synthetic” intergenic region composed of the termination signal for the M gene and the initiation signal for the reporter gene. The “native” intergenic region was left to provide a termination signal for the reporter gene and the initiation signal for the G gene. Relevant signal sequences, including the start and stop codons for the flanking genes, are highlighted in bold. (B) Growth curves of Tha, Tha-GFP, and Tha-Crimson viruses over 48 h of infection. BSR cells were infected at an MOI of 0.1, and the supernatant was recovered at 24 h, 48 h, and 72 h postinfection. Viruses were then titrated on BSR cells. Download FIG S1, PDF file, 0.07 MB. Copyright © 2019 Besson et al. 2019 Besson et al. This content is distributed under the terms of the Creative Commons Attribution 4.0 International license.

10.1128/mSphere.00047-19.2 FIG S2 RNAi screening protocol and controls. (A) Schematic of the RNAi screening. HEK293 cells were plated in 384-well plates together with siRNA for a final concentration of 25 nM. One plate was used to quantify the amount of cell per well and determine the proper MOI. After 72 h of siRNA treatment, HEK293T cells were infected for 18 or 36 h with Tha-GFP at an MOI of 5. At the experimental endpoint, cells were fixed with 4% PFA and subjected to Hoechst staining. (B to D) Both RABV (GFP) and nuclei (Hoechst) were quantified on an Operetta system. Images (B) and nucleus quantification data (C) and virus quantification data corresponding to Tha-GFP intensity (D) or the proportion of infected cells (E) are displayed here. Each result represents the mean of data from 9 fields in 3 distinct wells with 3 independent siRNA. *P* values (two-way ANOVA) are indicated (#, *P* < 0.0001). Download FIG S2, PDF file, 0.4 MB. Copyright © 2019 Besson et al. 2019 Besson et al. This content is distributed under the terms of the Creative Commons Attribution 4.0 International license.

10.1128/mSphere.00047-19.3 FIG S3 Phosphatidylinositol signaling system. Hits that affected RABV at 18 h (yellow) or 36 h (red) or both (orange) are indicated (see link on the KEGG database). Displayed proteins and enzymatic functions correspond to identified genes as follows: PIP5K = PIP5K1C, PIK3C = PIK3C2G, SHIP = INPPL1, IP3 3K = ITPKB, 3.1.3.57 = INPP1, 3.1.3.25 = IMPAD1, 3.1.3.64 = MTM1, 3.1.3.36 = INPP5E, and 3.1.3.95 = MTM1. (Reprinted from KEGG Pathway Maps with permission of the publisher.) Download FIG S3, PDF file, 0.10 MB. Copyright © 2019 Besson et al. 2019 Besson et al. This content is distributed under the terms of the Creative Commons Attribution 4.0 International license.

10.1128/mSphere.00047-19.4 FIG S4 Mannose and fructose metabolism. Hits that affected RABV at 18 h (yellow) or 36 h (red) or both (orange) are indicated (see link on the KEGG database). Displayed proteins and enzymatic functions correspond to identified genes as follows: 2.7.1.52 = FUK, 3.1.3.11 = FBP2, and 3.1.3.46 = PFKBP1/2/3. (Reprinted from KEGG Pathway Maps with permission of the publisher.) Download FIG S4, PDF file, 0.1 MB. Copyright © 2019 Besson et al. 2019 Besson et al. This content is distributed under the terms of the Creative Commons Attribution 4.0 International license.

10.1128/mSphere.00047-19.5 FIG S5 Effect of MAPK and PI3P pathway inhibitors on RABV infection in HEK293T cells. HEK293T cells were infected with Tha-Crimson at an MOI of 3 after 6 h of treatment with DMSO (0.5%) or 14 small molecules. Cells were fixed with 4% PFA at 18 h (A) or 36 h (B) postinfection. Images were acquired and analyzed to determine the relative numbers of nuclei (Nucleus Int.; blue) and the relative quantities of Tha-Crimson (E2-Crimson Int.; red). Each value represents the mean of data from 2 separate wells, and error bars represent standard deviations. Differences between compounds and DMSO were assessed using one-way ANOVA in Prism 7 (GraphPad). The *P* values are indicated for the lowest dose with a significant effect (indicated by a single asterisk [*]) and curves with a significant trend (indicated by a pound sign [#]). ###, *P* < 0.001; # or *, *P* < 0.05. The dotted line indicates the average value determined for Tha-GFP in a mixture with DMSO. Download FIG S5, PDF file, 0.2 MB. Copyright © 2019 Besson et al. 2019 Besson et al. This content is distributed under the terms of the Creative Commons Attribution 4.0 International license.

10.1128/mSphere.00047-19.6 FIG S6 Effect of MAPK and PI3P pathway inhibitors on RABV infection in human neurons. Human neurons differentiated from induced pluripotent stem cells (iPSC) were infected with Tha-Crimson at an MOI of 3 after 6 h of treatment with DMSO (0.5%) or 14 small molecules. Cells were fixed with 4% PFA at 18 h (A) or 36 h (B) postinfection. Images were acquired and analyzed to determine the relative numbers of nuclei (Nuclei Int.; blue) and the relative quantities of Tha-Crimson (E2-Crimson Int.; red). Each value represents the mean of data from 2 separate wells, and error bars represent standard deviations. Differences between compounds and DMSO were assessed using one-way ANOVA in Prism 7 (GraphPad). The *P* values are indicated for the lowest dose with a significant effect as follows: ***, *P* < 0.001; **, *P* < 0.01; *, *P* < 0.05. The dotted line represents the average value determined for Tha-Crimson in a mixture with DMSO. Download FIG S6, PDF file, 0.2 MB. Copyright © 2019 Besson et al. 2019 Besson et al. This content is distributed under the terms of the Creative Commons Attribution 4.0 International license.

Tha-GFP and Tha-Crimson virus were rescued as previously described (15). Full-length viral cDNA (2.5 μg) and plasmids N-pTIT (2.5 μg), P-pTIT (1.25 μg), and L-pTIT (1.25 μg) were transfected in 10^6^ BSR T7/5 cells (60) in 6-well plates. Cells were then passaged every 3 days until 100% of the cells were infected. The supernatant was harvested and titrated on BSR cells to determine the MOI. The insertion of the enhanced green fluorescent protein (eGFP) gene was shown to have no effect of viral replication compared to the results seen with the original Tha virus (Fig. S1B).

### Liquid-dispensing and automation system.

Several liquid-dispensing devices were used throughout this study. The siRNA duplexes were plated and transferred using a grade 384 stainless steel head with disposable low-volume polypropylene tips on a PP-384-M personal pipettor (Apricot Designs, Monrovia, CA). The addition of cell suspensions and solutions containing RABV was performed using a WellMate (Thermo Fisher Scientific, MA, USA), and cell fixation and staining were performed using an ELx405 automated washer (Biotek, VT, USA). Assay plates (Greiner Bio-One, NC, USA) were incubated in the tissue culture incubator (Thermo Fisher Scientific) under conditions of controlled humidity at 37°C and 5% CO_2_. The assay was performed in a biosafety level 3 (BSL-3) facility with adherence to safety and handling guidelines regarding infectious pathogens.

### Kinome-focused siRNA screening for host factors involved in lyssavirus infection.

The arrayed kinase-and-phosphatase-focused siRNA library (Ambion Silencer Select v4.0; Thermo Fisher Scientific) contains 3,024 siRNA duplexes covering 1,008 genes. The siRNA library was diluted from 20 μM stock solutions to a concentration of 0.25 μM, and 5-μl volumes were transferred into assay plates for a final testing concentration of 25 nM. The screening was performed in a simple biological and technical replicate, considering that for each gene, 3 independent siRNA duplexes were tested at two different time points. Internal references were included in each assay plate with Silencer Select Negative Control no. 1 siRNA (4390843) as a negative control (siNEG), PLK1 siRNA (s449) as a phenotypic control, and GFP siRNA as a functional control. For the reverse transfection procedure, 5 μl of a transfection mix containing 0.5 μl Dharmafect1 (GE Life Sciences, CO, USA)–Opti-MEM (Invitrogen) was added to each well and the reaction mixture was incubated for 20 min to promote complex formation. Next, HEK293T cells were dispensed into the assay plates at 2,000 cells per well in 40 μl of growth media and incubated with siRNA complexes for 72 h. One plate was used to determine the cell number present before infection. RABV solution was prepared in media for infection at an MOI of 5, and 10 μl was dispensed into the assay plates. To measure different time points of infection, the first set of assay plates were processed after 18 h for events occurring in the early stage (first round of infection) and the second set of assay plates were processed after 36 h for events occurring in the late stage (second round of infection and late infection). Cells were fixed in 2.5% paraformaldehyde (PFA) for 20 min followed by staining of nuclei in a solution of 1 μM Hoechst stain for 30 min. Images were acquired on an Operetta system (Perkin Elmer, MA, USA) for analysis of GFP fluorescence intensity and Hoechst-stained nuclei. A total of 4 images per well were acquired using a 10× magnifying objective and analyzed using Columbus software (Perkin Elmer).

### RNAi screening data analysis and selection.

Raw data were analyzed based on the biased discriminant analysis (BDA) method (61). Briefly, the BDA method was employed as follows. To identify host factor modulators of RABV, the active siRNA duplexes were scored using a threshold based on standard deviations of the means of GFP fluorescence intensity values from the siNEG control. Host factors that are important for RABV infection (“viral helpers”) were scored based on the −3σ threshold and the mean, and suppressor genes (“viral inhibitors”) were scored based on the +2σ threshold and the mean. Thresholds were determined empirically. Cytotoxic siRNA duplexes were scored based on the siNEG, and those showing a reduction in the count of nuclei greater than 70% (below the number of cells initially seeded) were deemed toxic. Host factors obtained with two or more cytotoxic siRNAs were labeled essential and not considered in the analysis.

### MAPK-focused and PI3K-focused compound screening and analysis.

NPCs were dissociated by the use of 3 ml Accutase (StemPro) and resuspended in NPC medium (1:1 N2 and B27 media, with 20 ng/ml bFGF, without vitamin A), and 3,000 cells were plated per well in 384-well plates. The next day, bFGF was withdrawn to initiate neuronal differentiation, and the medium was refreshed (using 1:1 N2 and B27 media, with vitamin A) every 2 to 3 days for 2 weeks. Differentiation of the NPCs into neurons was confirmed by Nluc quantification (data not shown), and they were further processed after they reached a high density combined with a low level of cell clustering. HEK293T cells were plated in 384-well plates with 4,000 cells/well and incubated for 24 h before compound treatment was performed.

Compounds were prepared in 2-fold dilutions in the 384-well format at 10 points, starting at 10 mM. On the day of experiment, cells were pretreated with compounds 6 h before infection. The compounds were tested in technical duplicate at 10 different concentrations and at two separate time points. One plate was used to determine the cell number before infection with Tha-E2 Crimson virus was performed at an MOI of 3 for neurons and with Tha-eGFP virus at an MOI of 5 for the 293T cells. During the following days, cells were fixed at 18 h and 36 h postinfection with 2.5% PFA and analyzed as described above.

### Bioinformatic analysis.

For each gene with a positive hit, the GFP intensity measured with each active single siRNA was normalized to the mean of the data from the siNEG population to provide a “GFP score” (%).

Networks were built using STRING v10.5 as follows: for hits to be considered high-confidence hits (i.e., with at least two positive single siRNA results), a “high-confidence score” (0.7) was required over 4 sources (text mining, experiments, databases, and coexpression), and for hits to be considered low-confidence hits (i.e., with only one positive single siRNA result), a “very-high-confidence score” (0.9) was required over 3 sources (text mining, experiments, and databases). The networks were then merged in Cytoscape v3.4, and only the genes not labeled “essential,” the genes labeled “high confidence,” and the genes labeled “low confidence” involved in a direct interaction with a gene labeled “high confidence” were displayed.

Signaling pathways were obtained using KEGG Mapper on the KEGG database (62–64).

### Statistical analysis.

Group comparisons of data were performed by one-way or two-way analysis of variance (ANOVA) using GraphPad Prism 7 software.
