# Supplementary material for: Kinome-Wide RNA Interference Screening Identifies Mitogen-Activated Protein Kinases and Phosphatidylinositol Metabolism as Key Factors for Rabies Virus Infection
Source: mSphere. 2019 May 22;4(3):e00047-19. doi: 10.1128/mSphere.00047-19 (PMC6531879; doi:10.1128/mSphere.00047-19)
Supplement: TABLE S1 [file mSphere.00047-19-st001.docx]

| **Gene Symbol** | **Full Gene Name** | **RefSeq#** | **Gene ID#** | **GFP score (av.)** | **GFP score (%)** | **Effect on virus** | **Effect on cells** | **Role** |
| --- | --- | --- | --- | --- | --- | --- | --- | --- |
| CHEK1 | CHK1 checkpoint homolog | NM_001274 | 1111 | 239039 | 18 | Inhibition (3/3) | Toxic | Essential |
| PLK1 | polo-like kinase 1 | NM_005030 | 5347 | 269359 | 21 | Inhibition (3/3) | Toxic | Essential |
| CDK11B | cyclin-dependent kinase 11B | NM_033486 | 984 | 398083 | 31 | Inhibition (2/3) | Toxic | Essential |
| DUSP26 | dual specificity phosphatase 26 (putative) | NM_024025 | 78986 | 569764 | 44 | Inhibition (2/3) | Toxic | Essential |
| TRRAP | transformation/transcription domain-associated protein | NM_003496 | 8295 | 622359 | 48 | Inhibition (2/3) | Toxic | Essential |
| WEE1 | WEE1 homolog | NM_003390 | 7465 | 247571 | 19 | Inhibition (2/3) | Toxic | Essential |
| CDC25C | cell division cycle 25 homolog C | NM_001790 | 995 | 586478 | 45 | Inhibition (3/3) | Non-Toxic | Viral helper |
| CHTF18 | CTF18, chromosome transmission fidelity factor 18 homolog | NM_022092 | 63922 | 547046 | 42 | Inhibition (3/3) | Non-Toxic | Viral helper |
| EPHA7 | EPH receptor A7 | NM_004440 | 2045 | 527672 | 41 | Inhibition (3/3) | Non-Toxic | Viral helper |
| PPP2CA | protein phosphatase 2, catalytic subunit, alpha isozyme | NM_002715 | 5515 | 685682 | 53 | Inhibition (3/3) | Non-Toxic | Viral helper |
| PTPRN | protein tyrosine phosphatase, receptor type, N | NM_002846 | 5798 | 685771 | 53 | Inhibition (3/3) | Non-Toxic | Viral helper |
| AURKA | aurora kinase A | NM_003600 | 6790 | 675901 | 52 | Inhibition (2/3) | Non-Toxic | Viral helper |
| BMP2K | BMP2 inducible kinase | NM_017593 | 55589 | 647851 | 50 | Inhibition (2/3) | Non-Toxic | Viral helper |
| DUSP22 | dual specificity phosphatase 22 | NM_020185 | 56940 | 689114 | 53 | Inhibition (2/3) | Non-Toxic | Viral helper |
| BUB1B | budding uninhibited by benzimidazoles 1 homolog beta | NM_001211 | 701 | 745215 | 57 | Inhibition (2/3) | Non-Toxic | Viral helper |
| DUSP5 | dual specificity phosphatase 5 | NM_004419 | 1847 | 571782 | 44 | Inhibition (2/3) | Non-Toxic | Viral helper |
| FGR | Gardner-Rasheed feline sarcoma viral (v-fgr) oncogene homolog | NM_001042729 | 2268 | 469057 | 36 | Inhibition (2/3) | Non-Toxic | Viral helper |
| DUSP7 | dual specificity phosphatase 7 | NM_001947 | 1849 | 560478 | 43 | Inhibition (2/3) | Non-Toxic | Viral helper |
| FRAP1 | FK506 binding protein 12-rapamycin associated protein 1 | NM_004958 | 2475 | 534893 | 41 | Inhibition (2/3) | Non-Toxic | Viral helper |
| KSR1 | kinase suppressor of ras 1 | NM_014238 | 8844 | 500179 | 38 | Inhibition (2/3) | Non-Toxic | Viral helper |
| DUSP9 | dual specificity phosphatase 9 | NM_001395 | 1852 | 578336 | 44 | Inhibition (2/3) | Non-Toxic | Viral helper |
| HDDC3 | HD domain containing 3 | NM_198527 | 374659 | 535343 | 41 | Inhibition (2/3) | Non-Toxic | Viral helper |
| MAGI2 | membrane associated guanylate kinase, WW and PDZ domain containing 2 | NM_012301 | 9863 | 715093 | 55 | Inhibition (2/3) | Non-Toxic | Viral helper |
| NEK4 | NIMA (never in mitosis gene a)-related kinase 4 | NM_003157 | 6787 | 620693 | 48 | Inhibition (2/3) | Non-Toxic | Viral helper |
| CLK1 | CDC-like kinase 1 | NM_004071 | 1195 | 678990 | 52 | Inhibition (2/3) | Non-Toxic | Viral helper |
| ICK | intestinal cell (MAK-like) kinase | NM_014920 | 22858 | 504853 | 39 | Inhibition (2/3) | Non-Toxic | Viral helper |
| MAP2K1 | mitogen-activated protein kinase kinase 1 | NM_002755 | 5604 | 621239 | 48 | Inhibition (2/3) | Non-Toxic | Viral helper |
| NEK7 | NIMA (never in mitosis gene a)-related kinase 7 | NM_133494 | 140609 | 488763 | 38 | Inhibition (2/3) | Non-Toxic | Viral helper |
| PFKFB1 | 6-phosphofructo-2-kinase/fructose-2,6-biphosphatase 1 | NM_002625 | 5207 | 569142 | 44 | Inhibition (2/3) | Non-Toxic | Viral helper |
| EPHB2 | EPH receptor B2 | NM_004442 | 2048 | 702327 | 54 | Inhibition (2/3) | Non-Toxic | Viral helper |
| INPP5E | inositol polyphosphate-5-phosphatase, 72 kDa | NM_019892 | 56623 | 714055 | 55 | Inhibition (2/3) | Non-Toxic | Viral helper |
| MAP3K11 | mitogen-activated protein kinase kinase kinase 11 | NM_002419 | 4296 | 439345 | 34 | Inhibition (2/3) | Non-Toxic | Viral helper |
| NUDT8 | nudix (nucleoside diphosphate linked moiety X)-type motif 8 | NM_181843 | 254552 | 686379 | 53 | Inhibition (2/3) | Non-Toxic | Viral helper |
| PFKFB3 | 6-phosphofructo-2-kinase/fructose-2,6-biphosphatase 3 | NM_004566 | 5209 | 655851 | 50 | Inhibition (2/3) | Non-Toxic | Viral helper |
| INPPL1 | inositol polyphosphate phosphatase-like 1 | NM_001567 | 3636 | 629572 | 48 | Inhibition (2/3) | Non-Toxic | Viral helper |
| MINPP1 | multiple inositol polyphosphate histidine phosphatase, 1 | NM_004897 | 9562 | 479086 | 37 | Inhibition (2/3) | Non-Toxic | Viral helper |
| PAK4 | p21 protein (Cdc42/Rac)-activated kinase 4 | NM_001014831 | 10298 | 666077 | 51 | Inhibition (2/3) | Non-Toxic | Viral helper |
| PIK3C2G | phosphoinositide-3-kinase, class 2, gamma polypeptide | NM_004570 | 5288 | 778879 | 60 | Inhibition (2/3) | Non-Toxic | Viral helper |
| MTMR4 | myotubularin related protein 4 | NM_004687 | 9110 | 559013 | 43 | Inhibition (2/3) | Non-Toxic | Viral helper |
| PAK6 | p21 protein (Cdc42/Rac)-activated kinase 6 | NM_020168 | 56924 | 318371 | 24 | Inhibition (2/3) | Non-Toxic | Viral helper |
| PPAP2B | phosphatidic acid phosphatase type 2B | NM_003713 | 8613 | 688169 | 53 | Inhibition (2/3) | Non-Toxic | Viral helper |
| PDK4 | pyruvate dehydrogenase kinase, isozyme 4 | NM_002612 | 5166 | 404650 | 31 | Inhibition (2/3) | Non-Toxic | Viral helper |
| PPM1A | protein phosphatase 1A (formerly 2C), magnesium-dependent, alpha isoform | NM_177951 | 5494 | 716657 | 55 | Inhibition (2/3) | Non-Toxic | Viral helper |
| PRKD3 | protein kinase D3 | NM_005813 | 23683 | 614158 | 47 | Inhibition (2/3) | Non-Toxic | Viral helper |
| PRPF4B | PRP4 pre-mRNA processing factor 4 homolog B | NM_003913 | 8899 | 710584 | 55 | Inhibition (2/3) | Non-Toxic | Viral helper |
| PTK7 | PTK7 protein tyrosine kinase 7 | NM_002821 | 5754 | 605684 | 47 | Inhibition (2/3) | Non-Toxic | Viral helper |
| PTP4A1 | protein tyrosine phosphatase type IVA, member 1 | NM_003463 | 7803 | 694784 | 53 | Inhibition (2/3) | Non-Toxic | Viral helper |
| PTPRH | protein tyrosine phosphatase, receptor type, H | NM_002842 | 5794 | 438851 | 34 | Inhibition (2/3) | Non-Toxic | Viral helper |
| PXK | PX domain containing serine/threonine kinase | NM_017771 | 54899 | 431209 | 33 | Inhibition (2/3) | Non-Toxic | Viral helper |
| RIOK2 | RIO kinase 2 | NM_018343 | 55781 | 450541 | 35 | Inhibition (2/3) | Non-Toxic | Viral helper |
| RWDD2B | RWD domain containing 2B | NM_016940 | 10069 | 704506 | 54 | Inhibition (2/3) | Non-Toxic | Viral helper |
| SRC | v-src sarcoma (Schmidt-Ruppin A-2) viral oncogene homolog | NM_005417 | 6714 | 658896 | 51 | Inhibition (2/3) | Non-Toxic | Viral helper |
| STK17B | serine/threonine kinase 17b | NM_004226 | 9262 | 506617 | 39 | Inhibition (2/3) | Non-Toxic | Viral helper |
| STK38 | serine/threonine kinase 38 | NM_007271 | 11329 | 555822 | 43 | Inhibition (2/3) | Non-Toxic | Viral helper |
| TNK1 | tyrosine kinase, non-receptor, 1 | NM_003985 | 8711 | 617576 | 48 | Inhibition (2/3) | Non-Toxic | Viral helper |
| TNK2 | tyrosine kinase, non-receptor, 2 | NM_001010938 | 10188 | 469499 | 36 | Inhibition (2/3) | Non-Toxic | Viral helper |
| TRIB2 | tribbles homolog 2 | NM_021643 | 28951 | 500927 | 39 | Inhibition (2/3) | Non-Toxic | Viral helper |
| TSKS | testis-specific serine kinase substrate | NM_021733 | 60385 | 384024 | 30 | Inhibition (2/3) | Non-Toxic | Viral helper |
| TWF2 | twinfilin, actin-binding protein, homolog 2 | NM_007284 | 11344 | 726794 | 56 | Inhibition (2/3) | Non-Toxic | Viral helper |
| UCK1 | uridine-cytidine kinase 1 | NM_031432 | 83549 | 697419 | 54 | Inhibition (2/3) | Non-Toxic | Viral helper |
| PDGFRL | platelet-derived growth factor receptor-like | NM_006207 | 5157 | 2205658 | 170 | Activator (2/3) | Non-Toxic | Viral inhibitor |
| PKN2 | protein kinase N2 | NM_006256 | 5586 | 2288330 | 176 | Activator (2/3) | Non-Toxic | Viral inhibitor |
| PPAP2A | phosphatidic acid phosphatase type 2A | NM_003711 | 8611 | 2294643 | 177 | Activator (2/3) | Non-Toxic | Viral inhibitor |
| AATK | apoptosis-associated tyrosine kinase | NM_001080395 | 9625 | 786037 | 60 | Inhibition (1/3) | Non-Toxic | ? |
| ABL1 | c-abl oncogene 1, non-receptor tyrosine kinase | NM_007313 | 25 | 683319 | 53 | Inhibition (1/3) | Non-Toxic | ? |
| ACP5 | acid phosphatase 5, tartrate resistant | NM_001611 | 54 | 660255 | 51 | Inhibition (1/3) | Non-Toxic | ? |
| ACVRL1 | activin A receptor type II-like 1 | NM_000020 | 94 | 700145 | 54 | Inhibition (1/3) | Non-Toxic | ? |
| ADPGK | ADP-dependent glucokinase | NM_031284 | 83440 | 795833 | 61 | Inhibition (1/3) | Non-Toxic | ? |
| AK1 | adenylate kinase 1 | NM_000476 | 203 | 753670 | 58 | Inhibition (1/3) | Non-Toxic | ? |
| AK8 | adenylate kinase 8 | NM_152572 | 158067 | 638684 | 49 | Inhibition (1/3) | Non-Toxic | ? |
| AKT1 | v-akt murine thymoma viral oncogene homolog 1 | NM_001014431 | 207 | 778768 | 60 | Inhibition (1/3) | Non-Toxic | ? |
| AKT2 | v-akt murine thymoma viral oncogene homolog 2 | NM_001626 | 208 | 636523 | 49 | Inhibition (1/3) | Non-Toxic | ? |
| ALPI | alkaline phosphatase, intestinal | NM_001631 | 248 | 613983 | 47 | Inhibition (1/3) | Non-Toxic | ? |
| ANP32E | acidic (leucine-rich) nuclear phosphoprotein 32 family, member E | NM_030920 | 81611 | 391474 | 30 | Inhibition (1/3) | Non-Toxic | ? |
| ASB10 | ankyrin repeat and SOCS box-containing 10 | NM_080871 | 136371 | 664591 | 51 | Inhibition (1/3) | Non-Toxic | ? |
| AURKAIP1 | aurora kinase A interacting protein 1 | NM_017900 | 54998 | 653829 | 50 | Inhibition (1/3) | Non-Toxic | ? |
| AURKC | aurora kinase C | NM_001015878 | 6795 | 488702 | 38 | Inhibition (1/3) | Non-Toxic | ? |
| C3orf48 | chromosome 3 open reading frame 48 | NM_144714 | 151649 | 796262 | 61 | Inhibition (1/3) | Non-Toxic | ? |
| CAMK1G | calcium/calmodulin-dependent protein kinase IG | NM_020439 | 57172 | 697858 | 54 | Inhibition (1/3) | Non-Toxic | ? |
| CDC25A | cell division cycle 25 homolog A | NM_001789 | 993 | 781178 | 60 | Inhibition (1/3) | Non-Toxic | ? |
| CDC42BPG | CDC42 binding protein kinase gamma | NM_017525 | 55561 | 743471 | 57 | Inhibition (1/3) | Non-Toxic | ? |
| CDC42SE2 | CDC42 small effector 2 | NM_001038702 | 56990 | 568077 | 44 | Inhibition (1/3) | Non-Toxic | ? |
| CDK14 | cyclin-dependent kinase 14 | NM_012395 | 5218 | 423689 | 33 | Inhibition (1/3) | Non-Toxic | ? |
| CDK4 | cyclin-dependent kinase 4 | NM_000075 | 1019 | 689662 | 53 | Inhibition (1/3) | Non-Toxic | ? |
| CDK5 | cyclin-dependent kinase 5 | NM_004935 | 1020 | 600402 | 46 | Inhibition (1/3) | Non-Toxic | ? |
| CHEK2 | checkpoint kinase 2 | NM_001005735 | 11200 | 749148 | 58 | Inhibition (1/3) | Non-Toxic | ? |
| CHP1 | calcium binding protein P22 | NM_007236 | 11261 | 672374 | 52 | Inhibition (1/3) | Non-Toxic | ? |
| CHP2 | calcineurin B homologous protein 2 | NM_022097 | 63928 | 461843 | 36 | Inhibition (1/3) | Non-Toxic | ? |
| CIB1 | calcium and integrin binding 1 | NM_006384 | 10519 | 444313 | 34 | Inhibition (1/3) | Non-Toxic | ? |
| CIB3 | calcium and integrin binding family member 3 | NM_054113 | 117286 | 785254 | 60 | Inhibition (1/3) | Non-Toxic | ? |
| CRKRS | Cdc2-related kinase, arginine/serine-rich | NM_016507 | 51755 | 703413 | 54 | Inhibition (1/3) | Non-Toxic | ? |
| CTDP1 | CTD (carboxy-terminal domain, RNA polymerase II, polypeptide A) phosphatase, subunit 1 | NM_004715 | 9150 | 688875 | 53 | Inhibition (1/3) | Non-Toxic | ? |
| CTDSP1 | CTD (carboxy-terminal domain, RNA polymerase II, polypeptide A) small phosphatase 1 | NM_021198 | 58190 | 713785 | 55 | Inhibition (1/3) | Non-Toxic | ? |
| DDR1 | discoidin domain receptor tyrosine kinase 1 | NM_001954 | 780 | 600685 | 46 | Inhibition (1/3) | Non-Toxic | ? |
| DGKI | diacylglycerol kinase, iota | NM_004717 | 9162 | 731721 | 56 | Inhibition (1/3) | Non-Toxic | ? |
| DGKQ | diacylglycerol kinase, theta 110kDa | NM_001347 | 1609 | 790472 | 61 | Inhibition (1/3) | Non-Toxic | ? |
| DMPK | dystrophia myotonica-protein kinase | NM_001081560 | 1760 | 766273 | 59 | Inhibition (1/3) | Non-Toxic | ? |
| DNAJC6 | DnaJ (Hsp40) homolog, subfamily C, member 6 | NM_014787 | 9829 | 789636 | 61 | Inhibition (1/3) | Non-Toxic | ? |
| DOK1 | docking protein 1, 62kDa | NM_001381 | 1796 | 513632 | 40 | Inhibition (1/3) | Non-Toxic | ? |
| DOLPP1 | dolichyl pyrophosphate phosphatase 1 | NM_020438 | 57171 | 629370 | 48 | Inhibition (1/3) | Non-Toxic | ? |
| DSTYK | dual serine/threonine and tyrosine protein kinase | NM_015375 | 25778 | 512236 | 39 | Inhibition (1/3) | Non-Toxic | ? |
| DUSP10 | dual specificity phosphatase 10 | NM_007207 | 11221 | 772327 | 59 | Inhibition (1/3) | Non-Toxic | ? |
| DUSP13 | dual specificity phosphatase 13 | NM_001007271 | 51207 | 712607 | 55 | Inhibition (1/3) | Non-Toxic | ? |
| DUSP18 | dual specificity phosphatase 18 | NM_152511 | 150290 | 486135 | 37 | Inhibition (1/3) | Non-Toxic | ? |
| DUSP19 | dual specificity phosphatase 19 | NM_080876 | 142679 | 783201 | 60 | Inhibition (1/3) | Non-Toxic | ? |
| DUSP4 | dual specificity phosphatase 4 | NM_001394 | 1846 | 729864 | 56 | Inhibition (1/3) | Non-Toxic | ? |
| DUSP6 | dual specificity phosphatase 6 | NM_001946 | 1848 | 571676 | 44 | Inhibition (1/3) | Non-Toxic | ? |
| DUT | deoxyuridine triphosphatase | NM_001025248 | 1854 | 473240 | 36 | Inhibition (1/3) | Non-Toxic | ? |
| DYRK4 | dual-specificity tyrosine-(Y)-phosphorylation regulated kinase 4 | NM_003845 | 8798 | 570168 | 44 | Inhibition (1/3) | Non-Toxic | ? |
| EIF2AK1 | eukaryotic translation initiation factor 2-alpha kinase 1 | NM_014413 | 27102 | 560761 | 43 | Inhibition (1/3) | Non-Toxic | ? |
| ENTPD3 | ectonucleoside triphosphate diphosphohydrolase 3 | NM_001248 | 956 | 785900 | 60 | Inhibition (1/3) | Non-Toxic | ? |
| ENTPD8 | ectonucleoside triphosphate diphosphohydrolase 8 | NM_001033113 | 377841 | 729577 | 56 | Inhibition (1/3) | Non-Toxic | ? |
| EPHB4 | EPH receptor B4 | NM_004444 | 2050 | 420976 | 32 | Inhibition (1/3) | Non-Toxic | ? |
| FBP2 | fructose-1,6-bisphosphatase 2 | NM_003837 | 8789 | 540846 | 42 | Inhibition (1/3) | Non-Toxic | ? |
| FER | fer (fps/fes related) tyrosine kinase | NM_005246 | 2241 | 786341 | 60 | Inhibition (1/3) | Non-Toxic | ? |
| FGFR2 | fibroblast growth factor receptor 2 | NM_022970 | 2263 | 727351 | 56 | Inhibition (1/3) | Non-Toxic | ? |
| FGFR3 | fibroblast growth factor receptor 3 | NM_000142 | 2261 | 644868 | 50 | Inhibition (1/3) | Non-Toxic | ? |
| FGFR4 | fibroblast growth factor receptor 4 | NM_002011 | 2264 | 615473 | 47 | Inhibition (1/3) | Non-Toxic | ? |
| FGFRL1 | fibroblast growth factor receptor-like 1 | NM_001004356 | 53834 | 594110 | 46 | Inhibition (1/3) | Non-Toxic | ? |
| FLT4 | fms-related tyrosine kinase 4 | NM_002020 | 2324 | 634511 | 49 | Inhibition (1/3) | Non-Toxic | ? |
| FUK | fucokinase | NM_145059 | 197258 | 520840 | 40 | Inhibition (1/3) | Non-Toxic | ? |
| GCKR | glucokinase (hexokinase 4) regulator | NM_001486 | 2646 | 714485 | 55 | Inhibition (1/3) | Non-Toxic | ? |
| GLYCTK | glycerate kinase | NM_145262 | 132158 | 614016 | 47 | Inhibition (1/3) | Non-Toxic | ? |
| GRK5 | G protein-coupled receptor kinase 5 | NM_005308 | 2869 | 548691 | 42 | Inhibition (1/3) | Non-Toxic | ? |
| HK2 | hexokinase 2 | NM_000189 | 3099 | 615138 | 47 | Inhibition (1/3) | Non-Toxic | ? |
| IKBKB | inhibitor of kappa light polypeptide gene enhancer in B-cells, kinase beta | NM_001556 | 3551 | 530637 | 41 | Inhibition (1/3) | Non-Toxic | ? |
| IKBKE | inhibitor of kappa light polypeptide gene enhancer in B-cells, kinase epsilon | NM_014002 | 9641 | 679164 | 52 | Inhibition (1/3) | Non-Toxic | ? |
| INPP5D | inositol polyphosphate-5-phosphatase, 145kDa | NM_001017915 | 3635 | 753408 | 58 | Inhibition (1/3) | Non-Toxic | ? |
| INPP5J | inositol polyphosphate-5-phosphatase J | NM_001002837 | 27124 | 704674 | 54 | Inhibition (1/3) | Non-Toxic | ? |
| INPP5K | inositol polyphosphate-5-phosphatase K | NM_130766 | 51763 | 497557 | 38 | Inhibition (1/3) | Non-Toxic | ? |
| INSR | insulin receptor | NM_000208 | 3643 | 631524 | 49 | Inhibition (1/3) | Non-Toxic | ? |
| IQCH | IQ motif containing H | NM_001031715 | 64799 | 733328 | 56 | Inhibition (1/3) | Non-Toxic | ? |
| ITGB1BP3 | integrin beta 1 binding protein 3 | NM_014446 | 27231 | 444865 | 34 | Inhibition (1/3) | Non-Toxic | ? |
| ITK | IL2-inducible T-cell kinase | NM_005546 | 3702 | 712899 | 55 | Inhibition (1/3) | Non-Toxic | ? |
| ITPK1 | inositol 1,3,4-triphosphate 5/6 kinase | NM_014216 | 3705 | 537536 | 41 | Inhibition (1/3) | Non-Toxic | ? |
| ITPKA | inositol 1,4,5-trisphosphate 3-kinase A | NM_002220 | 3706 | 785316 | 60 | Inhibition (1/3) | Non-Toxic | ? |
| KDR | kinase insert domain receptor | NM_002253 | 3791 | 784774 | 60 | Inhibition (1/3) | Non-Toxic | ? |
| KHK | ketohexokinase | NM_006488 | 3795 | 621398 | 48 | Inhibition (1/3) | Non-Toxic | ? |
| LATS1 | LATS, large tumor suppressor, homolog 1 | NM_004690 | 9113 | 595372 | 46 | Inhibition (1/3) | Non-Toxic | ? |
| LHPP | phospholysine phosphohistidine inorganic pyrophosphate phosphatase | NM_022126 | 64077 | 755356 | 58 | Inhibition (1/3) | Non-Toxic | ? |
| LPPR1 | lipid phosphate phosphatase-related protein type 1 | NM_017753 | 54886 | 584116 | 45 | Inhibition (1/3) | Non-Toxic | ? |
| LPPR5 | lipid phosphate phosphatase-related protein type 5 | NM_001010861 | 163404 | 597506 | 46 | Inhibition (1/3) | Non-Toxic | ? |
| LYN | v-yes-1 Yamaguchi sarcoma viral related oncogene homolog | NM_002350 | 4067 | 727344 | 56 | Inhibition (1/3) | Non-Toxic | ? |
| MAP2K3 | mitogen-activated protein kinase kinase 3 | NM_145109 | 5606 | 727284 | 56 | Inhibition (1/3) | Non-Toxic | ? |
| MAP2K7 | mitogen-activated protein kinase kinase 7 | NM_145185 | 5609 | 586247 | 45 | Inhibition (1/3) | Non-Toxic | ? |
| MAP3K10 | mitogen-activated protein kinase kinase kinase 10 | NM_002446 | 4294 | 558239 | 43 | Inhibition (1/3) | Non-Toxic | ? |
| MAP3K14 | mitogen-activated protein kinase kinase kinase 14 | NM_003954 | 9020 | 572652 | 44 | Inhibition (1/3) | Non-Toxic | ? |
| MAP3K9 | mitogen-activated protein kinase kinase kinase 9 | NM_033141 | 4293 | 669595 | 52 | Inhibition (1/3) | Non-Toxic | ? |
| MAPK11 | mitogen-activated protein kinase 11 | NM_002751 | 5600 | 617188 | 47 | Inhibition (1/3) | Non-Toxic | ? |
| MAPK12 | mitogen-activated protein kinase 12 | NM_002969 | 6300 | 753062 | 58 | Inhibition (1/3) | Non-Toxic | ? |
| MAPK13 | mitogen-activated protein kinase 13 | NM_002754 | 5603 | 444563 | 34 | Inhibition (1/3) | Non-Toxic | ? |
| MAPK8 | mitogen-activated protein kinase 8 | NM_002750 | 5599 | 749795 | 58 | Inhibition (1/3) | Non-Toxic | ? |
| MASTL | microtubule associated serine/threonine kinase-like | NM_032844 | 84930 | 658696 | 51 | Inhibition (1/3) | Non-Toxic | ? |
| MATK | megakaryocyte-associated tyrosine kinase | NM_002378 | 4145 | 653487 | 50 | Inhibition (1/3) | Non-Toxic | ? |
| MFN1 | mitofusin 1 | NM_033540 | 55669 | 588448 | 45 | Inhibition (1/3) | Non-Toxic | ? |
| MFN1 | similar to mitofusin 1 | XM_937130 | 441511 | 710163 | 55 | Inhibition (1/3) | Non-Toxic | ? |
| MFN2 | mitofusin 2 | NM_014874 | 9927 | 633918 | 49 | Inhibition (1/3) | Non-Toxic | ? |
| MOS | v-mos Moloney murine sarcoma viral oncogene homolog | NM_005372 | 4342 | 646057 | 50 | Inhibition (1/3) | Non-Toxic | ? |
| MPP1 | membrane protein, palmitoylated 1, 55kDa | NM_002436 | 4354 | 484854 | 37 | Inhibition (1/3) | Non-Toxic | ? |
| MPP3 | membrane protein, palmitoylated 3 (MAGUK p55 subfamily member 3) | NM_001932 | 4356 | 775087 | 60 | Inhibition (1/3) | Non-Toxic | ? |
| MPP4 | membrane protein, palmitoylated 4 (MAGUK p55 subfamily member 4) | NM_033066 | 58538 | 696165 | 54 | Inhibition (1/3) | Non-Toxic | ? |
| MST1R | macrophage stimulating 1 receptor | NM_002447 | 4486 | 607824 | 47 | Inhibition (1/3) | Non-Toxic | ? |
| MTMR8 | myotubularin related protein 8 | NM_017677 | 55613 | 686798 | 53 | Inhibition (1/3) | Non-Toxic | ? |
| MVK | mevalonate kinase | NM_000431 | 4598 | 537807 | 41 | Inhibition (1/3) | Non-Toxic | ? |
| MYLK2 | myosin light chain kinase 2 | NM_033118 | 85366 | 760284 | 58 | Inhibition (1/3) | Non-Toxic | ? |
| N/A | similar to template acyivating factor-I alpha | XM_371701 | 389217 | 635747 | 49 | Inhibition (1/3) | Non-Toxic | ? |
| N/A | similar to template acyivating factor-I alpha | XM_371701 | 389217 | 658411 | 51 | Inhibition (1/3) | Non-Toxic | ? |
| NEK11 | NIMA (never in mitosis gene a)- related kinase 11 | NM_024800 | 79858 | 784709 | 60 | Inhibition (1/3) | Non-Toxic | ? |
| NME4 | non-metastatic cells 4, protein expressed in | NM_005009 | 4833 | 708175 | 54 | Inhibition (1/3) | Non-Toxic | ? |
| NRGN | neurogranin | NM_006176 | 4900 | 648967 | 50 | Inhibition (1/3) | Non-Toxic | ? |
| NT5C2 | 5'-nucleotidase, cytosolic II | NM_012229 | 22978 | 777299 | 60 | Inhibition (1/3) | Non-Toxic | ? |
| NUDT6 | nudix (nucleoside diphosphate linked moiety X)-type motif 6 | NM_007083 | 11162 | 631436 | 49 | Inhibition (1/3) | Non-Toxic | ? |
| PAK7 | p21 protein (Cdc42/Rac)-activated kinase 7 | NM_020341 | 57144 | 723479 | 56 | Inhibition (1/3) | Non-Toxic | ? |
| PAPL | purple acid phosphatase long form | NM_001004318 | 390928 | 626456 | 48 | Inhibition (1/3) | Non-Toxic | ? |
| PAPSS1 | 3'-phosphoadenosine 5'-phosphosulfate synthase 1 | NM_005443 | 9061 | 719904 | 55 | Inhibition (1/3) | Non-Toxic | ? |
| PCTK2 | PCTAIRE protein kinase 2 | NM_002595 | 5128 | 743994 | 57 | Inhibition (1/3) | Non-Toxic | ? |
| PDGFRB | platelet-derived growth factor receptor, beta polypeptide | NM_002609 | 5159 | 535532 | 41 | Inhibition (1/3) | Non-Toxic | ? |
| PDK1 | pyruvate dehydrogenase kinase, isozyme 1 | NM_002610 | 5163 | 794653 | 61 | Inhibition (1/3) | Non-Toxic | ? |
| PDK3 | pyruvate dehydrogenase kinase, isozyme 3 | NM_005391 | 5165 | 727612 | 56 | Inhibition (1/3) | Non-Toxic | ? |
| PFKFB2 | 6-phosphofructo-2-kinase/fructose-2,6-biphosphatase 2 | NM_001018053 | 5208 | 762766 | 59 | Inhibition (1/3) | Non-Toxic | ? |
| PFKFB4 | 6-phosphofructo-2-kinase/fructose-2,6-biphosphatase 4 | NM_004567 | 5210 | 725290 | 56 | Inhibition (1/3) | Non-Toxic | ? |
| PFKM | phosphofructokinase, muscle | NM_000289 | 5213 | 686038 | 53 | Inhibition (1/3) | Non-Toxic | ? |
| PGK1 | phosphoglycerate kinase 1 | NM_000291 | 5230 | 532196 | 41 | Inhibition (1/3) | Non-Toxic | ? |
| PHACTR1 | phosphatase and actin regulator 1 | NM_030948 | 221692 | 757853 | 58 | Inhibition (1/3) | Non-Toxic | ? |
| PHKG2 | phosphorylase kinase, gamma 2 | NM_000294 | 5261 | 762496 | 59 | Inhibition (1/3) | Non-Toxic | ? |
| PI4K2A | phosphatidylinositol 4-kinase type 2 alpha | NM_018425 | 55361 | 752558 | 58 | Inhibition (1/3) | Non-Toxic | ? |
| PIK3AP1 | phosphoinositide-3-kinase adaptor protein 1 | NM_152309 | 118788 | 625694 | 48 | Inhibition (1/3) | Non-Toxic | ? |
| PIP4K2B | phosphatidylinositol-5-phosphate 4-kinase, type II, beta | NM_003559 | 8396 | 503598 | 39 | Inhibition (1/3) | Non-Toxic | ? |
| PIP5K1C | phosphatidylinositol-4-phosphate 5-kinase, type I, gamma | NM_012398 | 23396 | 626595 | 48 | Inhibition (1/3) | Non-Toxic | ? |
| PKM2 | pyruvate kinase, muscle | NM_002654 | 5315 | 681364 | 52 | Inhibition (1/3) | Non-Toxic | ? |
| PKN1 | protein kinase N1 | NM_002741 | 5585 | 596235 | 46 | Inhibition (1/3) | Non-Toxic | ? |
| PLK2 | polo-like kinase 2 | NM_006622 | 10769 | 477558 | 37 | Inhibition (1/3) | Non-Toxic | ? |
| PLXND1 | plexin D1 | NM_015103 | 23129 | 718230 | 55 | Inhibition (1/3) | Non-Toxic | ? |
| PNCK | pregnancy up-regulated non-ubiquitously expressed CaM kinase | NM_001039582 | 139728 | 380710 | 29 | Inhibition (1/3) | Non-Toxic | ? |
| PPAPDC2 | phosphatidic acid phosphatase type 2 domain containing 2 | NM_203453 | 403313 | 746097 | 57 | Inhibition (1/3) | Non-Toxic | ? |
| PPEF1 | protein phosphatase, EF-hand calcium binding domain 1 | NM_006240 | 5475 | 669584 | 52 | Inhibition (1/3) | Non-Toxic | ? |
| PPM1F | protein phosphatase, Mg2+/Mn2+ dependent, 1F | NM_014634 | 9647 | 713601 | 55 | Inhibition (1/3) | Non-Toxic | ? |
| PPP1R11 | protein phosphatase 1, regulatory (inhibitor) subunit 11 | NM_021959 | 6992 | 569320 | 44 | Inhibition (1/3) | Non-Toxic | ? |
| PPP1R12A | protein phosphatase 1, regulatory (inhibitor) subunit 12A | NM_002480 | 4659 | 720749 | 55 | Inhibition (1/3) | Non-Toxic | ? |
| PPP1R12B | protein phosphatase 1, regulatory (inhibitor) subunit 12B | NM_032104 | 4660 | 763314 | 59 | Inhibition (1/3) | Non-Toxic | ? |
| PPP1R14D | protein phosphatase 1, regulatory (inhibitor) subunit 14D | NM_017726 | 54866 | 657565 | 51 | Inhibition (1/3) | Non-Toxic | ? |
| PPP1R1A | protein phosphatase 1, regulatory (inhibitor) subunit 1A | NM_006741 | 5502 | 583870 | 45 | Inhibition (1/3) | Non-Toxic | ? |
| PPP1R1B | protein phosphatase 1, regulatory (inhibitor) subunit 1B | NM_032192 | 84152 | 726746 | 56 | Inhibition (1/3) | Non-Toxic | ? |
| PPP1R1C | protein phosphatase 1, regulatory (inhibitor) subunit 1C | NM_001080545 | 151242 | 769152 | 59 | Inhibition (1/3) | Non-Toxic | ? |
| PPP1R2 | protein phosphatase 1, regulatory (inhibitor) subunit 2 | NM_006241 | 5504 | 735513 | 57 | Inhibition (1/3) | Non-Toxic | ? |
| PPP1R3B | protein phosphatase 1, regulatory (inhibitor) subunit 3B | NM_024607 | 79660 | 635943 | 49 | Inhibition (1/3) | Non-Toxic | ? |
| PPP1R3C | protein phosphatase 1, regulatory (inhibitor) subunit 3C | NM_005398 | 5507 | 760779 | 59 | Inhibition (1/3) | Non-Toxic | ? |
| PPP1R7 | protein phosphatase 1, regulatory subunit 7 | NM_002712 | 5510 | 615238 | 47 | Inhibition (1/3) | Non-Toxic | ? |
| PPP1R8 | protein phosphatase 1, regulatory (inhibitor) subunit 8 | NM_002713 | 5511 | 565614 | 44 | Inhibition (1/3) | Non-Toxic | ? |
| PPP2R2C | protein phosphatase 2, regulatory subunit B, gamma | NM_181876 | 5522 | 589256 | 45 | Inhibition (1/3) | Non-Toxic | ? |
| PRKACB | protein kinase, cAMP-dependent, catalytic, beta | NM_002731 | 5567 | 760512 | 59 | Inhibition (1/3) | Non-Toxic | ? |
| PRKCA | protein kinase C, alpha | NM_002737 | 5578 | 583178 | 45 | Inhibition (1/3) | Non-Toxic | ? |
| PRKCSH | protein kinase C substrate 80K-H | NM_001001329 | 5589 | 759410 | 58 | Inhibition (1/3) | Non-Toxic | ? |
| PRKD1 | protein kinase D1 | NM_002742 | 5587 | 656197 | 50 | Inhibition (1/3) | Non-Toxic | ? |
| PRPS1 | phosphoribosyl pyrophosphate synthetase 1 | NM_002764 | 5631 | 398454 | 31 | Inhibition (1/3) | Non-Toxic | ? |
| PTK2B | PTK2B protein tyrosine kinase 2 beta | NM_004103 | 2185 | 545390 | 42 | Inhibition (1/3) | Non-Toxic | ? |
| PTP4A3 | protein tyrosine phosphatase type IVA, member 3 | NM_007079 | 11156 | 576502 | 44 | Inhibition (1/3) | Non-Toxic | ? |
| PTPN11 | protein tyrosine phosphatase, non-receptor type 11 | NM_002834 | 5781 | 683306 | 53 | Inhibition (1/3) | Non-Toxic | ? |
| PTPN20A | protein tyrosine phosphatase, non-receptor type 20A | NM_001042387 | 653129 | 742327 | 57 | Inhibition (1/3) | Non-Toxic | ? |
| PTPN21 | protein tyrosine phosphatase, non-receptor type 21 | NM_007039 | 11099 | 352648 | 27 | Inhibition (1/3) | Non-Toxic | ? |
| PTPN5 | protein tyrosine phosphatase, non-receptor type 5 | NM_001039970 | 84867 | 760972 | 59 | Inhibition (1/3) | Non-Toxic | ? |
| PTPRM | protein tyrosine phosphatase, receptor type, M | NM_002845 | 5797 | 666350 | 51 | Inhibition (1/3) | Non-Toxic | ? |
| PTPRS | protein tyrosine phosphatase, receptor type, S | NM_002850 | 5802 | 592852 | 46 | Inhibition (1/3) | Non-Toxic | ? |
| RAF1 | v-raf-1 murine leukemia viral oncogene homolog 1 | NM_002880 | 5894 | 541166 | 42 | Inhibition (1/3) | Non-Toxic | ? |
| RIOK3 | RIO kinase 3 | NM_003831 | 8780 | 571741 | 44 | Inhibition (1/3) | Non-Toxic | ? |
| RNGTT | RNA guanylyltransferase and 5'-phosphatase | NM_003800 | 8732 | 659823 | 51 | Inhibition (1/3) | Non-Toxic | ? |
| ROCK1 | Rho-associated, coiled-coil containing protein kinase 1 | NM_005406 | 6093 | 780754 | 60 | Inhibition (1/3) | Non-Toxic | ? |
| ROR1 | receptor tyrosine kinase-like orphan receptor 1 | NM_001083592 | 4919 | 640730 | 49 | Inhibition (1/3) | Non-Toxic | ? |
| RPS6KA4 | ribosomal protein S6 kinase, 90kDa, polypeptide 4 | NM_001006944 | 8986 | 644975 | 50 | Inhibition (1/3) | Non-Toxic | ? |
| RPS6KA6 | ribosomal protein S6 kinase, 90kDa, polypeptide 6 | NM_014496 | 27330 | 782100 | 60 | Inhibition (1/3) | Non-Toxic | ? |
| RPS6KB2 | ribosomal protein S6 kinase, 70kDa, polypeptide 2 | NM_003952 | 6199 | 782191 | 60 | Inhibition (1/3) | Non-Toxic | ? |
| RPS6KL1 | ribosomal protein S6 kinase-like 1 | NM_031464 | 83694 | 646887 | 50 | Inhibition (1/3) | Non-Toxic | ? |
| RYK | receptor-like tyrosine kinase | NM_001005861 | 6259 | 703258 | 54 | Inhibition (1/3) | Non-Toxic | ? |
| SBF1 | SET binding factor 1 | NM_002972 | 6305 | 615767 | 47 | Inhibition (1/3) | Non-Toxic | ? |
| SBF2 | SET binding factor 2 | NM_030962 | 81846 | 621458 | 48 | Inhibition (1/3) | Non-Toxic | ? |
| SBK1 | SH3-binding domain kinase 1 | NM_001024401 | 388228 | 593703 | 46 | Inhibition (1/3) | Non-Toxic | ? |
| SCYL2 | SCY1-like 2 (S. cerevisiae) | NM_017988 | 55681 | 788232 | 61 | Inhibition (1/3) | Non-Toxic | ? |
| SGPP2 | sphingosine-1-phosphate phosphatase 2 | NM_152386 | 130367 | 774896 | 60 | Inhibition (1/3) | Non-Toxic | ? |
| SH3BP4 | SH3-domain binding protein 4 | NM_014521 | 23677 | 747286 | 57 | Inhibition (1/3) | Non-Toxic | ? |
| SH3BP5L | SH3-binding domain protein 5-like | NM_030645 | 80851 | 396450 | 30 | Inhibition (1/3) | Non-Toxic | ? |
| SPHK2 | sphingosine kinase 2 | NM_020126 | 56848 | 580209 | 45 | Inhibition (1/3) | Non-Toxic | ? |
| SRMS | src-related kinase lacking C-terminal regulatory tyrosine and N-terminal myristylation sites | NM_080823 | 6725 | 704750 | 54 | Inhibition (1/3) | Non-Toxic | ? |
| STRADB | STE20-related kinase adaptor beta | NM_018571 | 55437 | 662713 | 51 | Inhibition (1/3) | Non-Toxic | ? |
| SYNJ2 | synaptojanin 2 | NM_003898 | 8871 | 559467 | 43 | Inhibition (1/3) | Non-Toxic | ? |
| TESK2 | testis-specific kinase 2 | NM_007170 | 10420 | 788956 | 61 | Inhibition (1/3) | Non-Toxic | ? |
| TLK1 | tousled-like kinase 1 | NM_012290 | 9874 | 567849 | 44 | Inhibition (1/3) | Non-Toxic | ? |
| TNNI3K | TNNI3 interacting kinase | NM_015978 | 51086 | 641127 | 49 | Inhibition (1/3) | Non-Toxic | ? |
| TPTE | transmembrane phosphatase with tensin homology | NM_199259 | 7179 | 644109 | 50 | Inhibition (1/3) | Non-Toxic | ? |
| TRAF3IP3 | TRAF3 interacting protein 3 | NM_025228 | 80342 | 594276 | 46 | Inhibition (1/3) | Non-Toxic | ? |
| TTBK1 | tau tubulin kinase 1 | NM_032538 | 84630 | 443909 | 34 | Inhibition (1/3) | Non-Toxic | ? |
| TXK | TXK tyrosine kinase | NM_003328 | 7294 | 567142 | 44 | Inhibition (1/3) | Non-Toxic | ? |
| WNK1 | WNK lysine deficient protein kinase 1 | NM_018979 | 65125 | 507161 | 39 | Inhibition (1/3) | Non-Toxic | ? |
| ZAK | sterile alpha motif and leucine zipper containing kinase AZK | NM_016653 | 51776 | 666937 | 51 | Inhibition (1/3) | Non-Toxic | ? |
| ACVR2A | activin A receptor, type IIA | NM_001616 | 92 | 305548 | 24 | Inhibition (1/3) | Toxic | ? |
| ACYP1 | acylphosphatase 1, erythrocyte (common) type | NM_001107 | 97 | 483669 | 37 | Inhibition (1/3) | Toxic | ? |
| ATP6V0E2 | ATPase, H+ transporting V0 subunit e2 | NM_145230 | 155066 | 756128 | 58 | Inhibition (1/3) | Toxic | ? |
| AURKB | aurora kinase B | NM_004217 | 9212 | 383296 | 29 | Inhibition (1/3) | Toxic | ? |
| BMPR1A | bone morphogenetic protein receptor, type IA | NM_004329 | 657 | 612193 | 47 | Inhibition (1/3) | Toxic | ? |
| BRSK2 | BR serine/threonine kinase 2 | NM_003957 | 9024 | 407365 | 31 | Inhibition (1/3) | Toxic | ? |
| CALM1 | calmodulin 1 | NM_006888 | 801 | 420061 | 32 | Inhibition (1/3) | Toxic | ? |
| CAMK1D | calcium/calmodulin-dependent protein kinase ID | NM_020397 | 57118 | 491817 | 38 | Inhibition (1/3) | Toxic | ? |
| CCDC155 | coiled-coil domain containing 155 | NM_144688 | 147872 | 397317 | 31 | Inhibition (1/3) | Toxic | ? |
| CDC2 | cell division cycle 2, G1 to S and G2 to M | NM_001786 | 983 | 696586 | 54 | Inhibition (1/3) | Toxic | ? |
| CDC42BPB | CDC42 binding protein kinase beta | NM_006035 | 9578 | 795455 | 61 | Inhibition (1/3) | Toxic | ? |
| CDK15 | cyclin-dependent kinase 15 | NM_139158 | 65061 | 415857 | 32 | Inhibition (1/3) | Toxic | ? |
| CDK19 | cyclin-dependent kinase 19 | NM_015076 | 23097 | 538173 | 41 | Inhibition (1/3) | Toxic | ? |
| CSNK1A1 | casein kinase 1, alpha 1 | NM_001025105 | 1452 | 247995 | 19 | Inhibition (1/3) | Toxic | ? |
| CSNK1G2 | casein kinase 1, gamma 2 | NM_001319 | 1455 | 324552 | 25 | Inhibition (1/3) | Toxic | ? |
| DGKK | diacylglycerol kinase, kappa | NM_001013742 | 139189 | 406564 | 31 | Inhibition (1/3) | Toxic | ? |
| DGUOK | deoxyguanosine kinase | NM_080916 | 1716 | 504623 | 39 | Inhibition (1/3) | Toxic | ? |
| DUSP1 | dual specificity phosphatase 1 | NM_004417 | 1843 | 611184 | 47 | Inhibition (1/3) | Toxic | ? |
| ENTPD2 | ectonucleoside triphosphate diphosphohydrolase 2 | NM_001246 | 954 | 503020 | 39 | Inhibition (1/3) | Toxic | ? |
| ENTPD5 | ectonucleoside triphosphate diphosphohydrolase 5 | NM_001249 | 957 | 567301 | 44 | Inhibition (1/3) | Toxic | ? |
| FES | feline sarcoma oncogene | NM_002005 | 2242 | 579340 | 45 | Inhibition (1/3) | Toxic | ? |
| FIG4 | FIG4 homolog (S. cerevisiae) | NM_014845 | 9896 | 478269 | 37 | Inhibition (1/3) | Toxic | ? |
| FRK | fyn-related kinase | NM_002031 | 2444 | 378984 | 29 | Inhibition (1/3) | Toxic | ? |
| GALK2 | galactokinase 2 | NM_001001556 | 2585 | 497062 | 38 | Inhibition (1/3) | Toxic | ? |
| INSRR | insulin receptor-related receptor | NM_014215 | 3645 | 506661 | 39 | Inhibition (1/3) | Toxic | ? |
| JAK1 | Janus kinase 1 | NM_002227 | 3716 | 301259 | 23 | Inhibition (1/3) | Toxic | ? |
| LRGUK | leucine-rich repeats and guanylate kinase domain containing | NM_144648 | 136332 | 561127 | 43 | Inhibition (1/3) | Toxic | ? |
| LY6G5B | lymphocyte antigen 6 complex, locus G5B | NM_021221 | 58496 | 238930 | 18 | Inhibition (1/3) | Toxic | ? |
| MAP2K4 | mitogen-activated protein kinase kinase 4 | NM_003010 | 6416 | 731201 | 56 | Inhibition (1/3) | Toxic | ? |
| MAP3K5 | mitogen-activated protein kinase kinase kinase 5 | NM_005923 | 4217 | 412516 | 32 | Inhibition (1/3) | Toxic | ? |
| MAPK1 | mitogen-activated protein kinase 1 | NM_002745 | 5594 | 343091 | 26 | Inhibition (1/3) | Toxic | ? |
| MKNK1 | MAP kinase interacting serine/threonine kinase 1 | NM_003684 | 8569 | 734917 | 57 | Inhibition (1/3) | Toxic | ? |
| MOK | MOK protein kinase | NM_014226 | 5891 | 675920 | 52 | Inhibition (1/3) | Toxic | ? |
| MRC2 | mannose receptor, C type 2 | NM_006039 | 9902 | 328174 | 25 | Inhibition (1/3) | Toxic | ? |
| PDXP | pyridoxal (pyridoxine, vitamin B6) phosphatase | NM_020315 | 57026 | 496879 | 38 | Inhibition (1/3) | Toxic | ? |
| PICK1 | protein interacting with PRKCA 1 | NM_001039583 | 9463 | 510088 | 39 | Inhibition (1/3) | Toxic | ? |
| PINK1 | PTEN induced putative kinase 1 | NM_032409 | 65018 | 731401 | 56 | Inhibition (1/3) | Toxic | ? |
| PLXNB1 | plexin B1 | NM_002673 | 5364 | 541142 | 42 | Inhibition (1/3) | Toxic | ? |
| PPM1K | protein phosphatase 1K (PP2C domain containing) | NM_152542 | 152926 | 359284 | 28 | Inhibition (1/3) | Toxic | ? |
| PPM2C | protein phosphatase 2C, magnesium-dependent, catalytic subunit | NM_018444 | 54704 | 552357 | 42 | Inhibition (1/3) | Toxic | ? |
| PPP1CB | protein phosphatase 1, catalytic subunit, beta isozyme | NM_002709 | 5500 | 566825 | 44 | Inhibition (1/3) | Toxic | ? |
| PPP1R12C | protein phosphatase 1, regulatory subunit 12C | NM_017607 | 54776 | 483389 | 37 | Inhibition (1/3) | Toxic | ? |
| PPP1R14A | protein phosphatase 1, regulatory (inhibitor) subunit 14A | NM_033256 | 94274 | 511327 | 39 | Inhibition (1/3) | Toxic | ? |
| PPP2R1A | protein phosphatase 2 (formerly 2A), regulatory subunit A, alpha isoform | NM_014225 | 5518 | 367115 | 28 | Inhibition (1/3) | Toxic | ? |
| PPP2R3A | protein phosphatase 2 (formerly 2A), regulatory subunit B'', alpha | NM_002718 | 5523 | 425496 | 33 | Inhibition (1/3) | Toxic | ? |
| PPP2R3C | protein phosphatase 2, regulatory subunit B'', gamma | NM_017917 | 55012 | 534722 | 41 | Inhibition (1/3) | Toxic | ? |
| PPP2R5E | protein phosphatase 2, regulatory subunit B', epsilon isoform | NM_006246 | 5529 | 680951 | 52 | Inhibition (1/3) | Toxic | ? |
| PPP3CC | protein phosphatase 3, catalytic subunit, gamma isozyme | NM_005605 | 5533 | 312190 | 24 | Inhibition (1/3) | Toxic | ? |
| PRKAA1 | protein kinase, AMP-activated, alpha 1 catalytic subunit | NM_006251 | 5562 | 403104 | 31 | Inhibition (1/3) | Toxic | ? |
| PRKCD | protein kinase C, delta | NM_006254 | 5580 | 522838 | 40 | Inhibition (1/3) | Toxic | ? |
| PRKDC | protein kinase, DNA-activated, catalytic polypeptide | NM_001081640 | 5591 | 449987 | 35 | Inhibition (1/3) | Toxic | ? |
| PRPS1L1 | phosphoribosyl pyrophosphate synthetase 1-like 1 | NM_175886 | 221823 | 452719 | 35 | Inhibition (1/3) | Toxic | ? |
| PTK2 | PTK2 protein tyrosine kinase 2 | NM_005607 | 5747 | 386592 | 30 | Inhibition (1/3) | Toxic | ? |
| PTPN14 | protein tyrosine phosphatase, non-receptor type 14 | NM_005401 | 5784 | 467819 | 36 | Inhibition (1/3) | Toxic | ? |
| PTPN9 | protein tyrosine phosphatase, non-receptor type 9 | NM_002833 | 5780 | 249969 | 19 | Inhibition (1/3) | Toxic | ? |
| PTPRO | protein tyrosine phosphatase, receptor type, O | NM_002848 | 5800 | 734258 | 56 | Inhibition (1/3) | Toxic | ? |
| RIPK4 | receptor-interacting serine-threonine kinase 4 | NM_020639 | 54101 | 559411 | 43 | Inhibition (1/3) | Toxic | ? |
| RPS6KA5 | ribosomal protein S6 kinase, 90kDa, polypeptide 5 | NM_004755 | 9252 | 358752 | 28 | Inhibition (1/3) | Toxic | ? |
| SHPK | sedoheptulokinase | NM_013276 | 23729 | 725417 | 56 | Inhibition (1/3) | Toxic | ? |
| SSH1 | slingshot homolog 1 | NM_018984 | 54434 | 453055 | 35 | Inhibition (1/3) | Toxic | ? |
| STK39 | serine threonine kinase 39 | NM_013233 | 27347 | 453198 | 35 | Inhibition (1/3) | Toxic | ? |
| STK4 | serine/threonine kinase 4 | NM_006282 | 6789 | 361204 | 28 | Inhibition (1/3) | Toxic | ? |
| STYK1 | serine/threonine/tyrosine kinase 1 | NM_018423 | 55359 | 501098 | 39 | Inhibition (1/3) | Toxic | ? |
| TGFBR2 | transforming growth factor, beta receptor II | NM_001024847 | 7048 | 679025 | 52 | Inhibition (1/3) | Toxic | ? |
| TIE1 | tyrosine kinase with immunoglobulin-like and EGF-like domains 1 | NM_005424 | 7075 | 521934 | 40 | Inhibition (1/3) | Toxic | ? |
| TRIB3 | tribbles homolog 3 | NM_021158 | 57761 | 345784 | 27 | Inhibition (1/3) | Toxic | ? |
| TTK | TTK protein kinase | NM_003318 | 7272 | 729613 | 56 | Inhibition (1/3) | Toxic | ? |
