# Supplementary material for: Kinome-Wide RNA Interference Screening Identifies Mitogen-Activated Protein Kinases and Phosphatidylinositol Metabolism as Key Factors for Rabies Virus Infection
Source: mSphere. 2019 May 22;4(3):e00047-19. doi: 10.1128/mSphere.00047-19 (PMC6531879; doi:10.1128/mSphere.00047-19)
Supplement: TABLE S2 [file mSphere.00047-19-st002.docx]

| **Gene Symbol** | **Full Gene Name** | **RefSeq#** | **Gene ID#** | **GFP score (av.)** | **GFP score (%)** | **Effect on virus** | **Effect on cells** | **Role** |
| --- | --- | --- | --- | --- | --- | --- | --- | --- |
| AURKB | aurora kinase B | NM_004217 | 9212 | 1130785 | 25 | Inhibition (2/3) | Toxic | Essential |
| CDK11B | cyclin-dependent kinase 11B | NM_033486 | 984 | 401538 | 9 | Inhibition (3/3) | Toxic | Essential |
| CHEK1 | CHK1 checkpoint homolog | NM_001274 | 1111 | 89741 | 2 | Inhibition (3/3) | Toxic | Essential |
| CHTF18 | CTF18, chromosome transmission fidelity factor 18 homolog | NM_022092 | 63922 | 358911 | 8 | Inhibition (2/3) | Toxic | Essential |
| DUSP26 | dual specificity phosphatase 26 (putative) | NM_024025 | 78986 | 1429346 | 32 | Inhibition (2/3) | Toxic | Essential |
| PLK1 | polo-like kinase 1 | NM_005030 | 5347 | 165876 | 4 | Inhibition (3/3) | Toxic | Essential |
| PTK2 | PTK2 protein tyrosine kinase 2 | NM_005607 | 5747 | 1443550 | 32 | Inhibition (2/3) | Toxic | Essential |
| PTPN14 | protein tyrosine phosphatase, non-receptor type 14 | NM_005401 | 5784 | 444482 | 10 | Inhibition (2/3) | Toxic | Essential |
| SRC | v-src sarcoma (Schmidt-Ruppin A-2) viral oncogene homolog | NM_005417 | 6714 | 587771 | 13 | Inhibition (2/3) | Toxic | Essential |
| TRRAP | transformation/transcription domain-associated protein | NM_003496 | 8295 | 1976664 | 44 | Inhibition (3/3) | Toxic | Essential |
| WEE1 | WEE1 homolog (S. pombe) | NM_003390 | 7465 | 37980 | 1 | Inhibition (2/3) | Toxic | Essential |
| ANP32E | acidic (leucine-rich) nuclear phosphoprotein 32 family, member E | NM_030920 | 81611 | 2135627 | 47 | Inhibition (2/3) | Non-toxic | Viral helper |
| ASB10 | ankyrin repeat and SOCS box-containing 10 | NM_080871 | 136371 | 1571835 | 35 | Inhibition (2/3) | Non-toxic | Viral helper |
| ASNA1 | arsA arsenite transporter, ATP-binding, homolog 1 (bacterial) | NM_004317 | 439 | 778338 | 17 | Inhibition (2/3) | Non-toxic | Viral helper |
| ATP6V0E2 | ATPase, H+ transporting V0 subunit e2 | NM_145230 | 155066 | 2221918 | 49 | Inhibition (2/3) | Non-toxic | Viral helper |
| BUB1B | budding uninhibited by benzimidazoles 1 homolog beta (yeast) | NM_001211 | 701 | 1927314 | 43 | Inhibition (2/3) | Non-toxic | Viral helper |
| CAMK2A | calcium/calmodulin-dependent protein kinase II alpha | NM_015981 | 815 | 2722201 | 60 | Inhibition (2/3) | Non-toxic | Viral helper |
| CCDC155 | coiled-coil domain containing 155 | NM_144688 | 147872 | 1925510 | 43 | Inhibition (2/3) | Non-toxic | Viral helper |
| CDC25C | cell division cycle 25 homolog C (S. pombe) | NM_001790 | 995 | 2855149 | 63 | Inhibition (2/3) | Non-toxic | Viral helper |
| CDC2L5 | cell division cycle 2-like 5 (cholinesterase-related cell division controller) | NM_031267 | 8621 | 1860192 | 41 | Inhibition (2/3) | Non-toxic | Viral helper |
| CDC42BPG | CDC42 binding protein kinase gamma (DMPK-like) | NM_017525 | 55561 | 1468990 | 33 | Inhibition (2/3) | Non-toxic | Viral helper |
| CDK5 | cyclin-dependent kinase 5 | NM_004935 | 1020 | 2742954 | 61 | Inhibition (2/3) | Non-toxic | Viral helper |
| DAPK2 | death-associated protein kinase 2 | NM_014326 | 23604 | 1757053 | 39 | Inhibition (2/3) | Non-toxic | Viral helper |
| DUSP5 | dual specificity phosphatase 5 | NM_004419 | 1847 | 1443234 | 32 | Inhibition (3/3) | Non-toxic | Viral helper |
| EPHB2 | EPH receptor B2 | NM_004442 | 2048 | 1632847 | 36 | Inhibition (2/3) | Non-toxic | Viral helper |
| ERN1 | endoplasmic reticulum to nucleus signaling 1 | NM_001433 | 2081 | 2656404 | 59 | Inhibition (2/3) | Non-toxic | Viral helper |
| FBP2 | fructose-1,6-bisphosphatase 2 | NM_003837 | 8789 | 1264888 | 28 | Inhibition (2/3) | Non-toxic | Viral helper |
| FGR | Gardner-Rasheed feline sarcoma viral (v-fgr) oncogene homolog | NM_001042729 | 2268 | 424483 | 9 | Inhibition (2/3) | Non-toxic | Viral helper |
| FUK | fucokinase | NM_145059 | 197258 | 2781274 | 62 | Inhibition (2/3) | Non-toxic | Viral helper |
| GLYCTK | glycerate kinase | NM_145262 | 132158 | 2279568 | 51 | Inhibition (2/3) | Non-toxic | Viral helper |
| IMPAD1 | inositol monophosphatase domain containing 1 | NM_017813 | 54928 | 2486915 | 55 | Inhibition (2/3) | Non-toxic | Viral helper |
| INPP1 | inositol polyphosphate-1-phosphatase | NM_002194 | 3628 | 1903394 | 42 | Inhibition (2/3) | Non-toxic | Viral helper |
| INPP5E | inositol polyphosphate-5-phosphatase, 72 kDa | NM_019892 | 56623 | 1745212 | 39 | Inhibition (2/3) | Non-toxic | Viral helper |
| ITPKB | inositol 1,4,5-trisphosphate 3-kinase B | NM_002221 | 3707 | 1359158 | 30 | Inhibition (2/3) | Non-toxic | Viral helper |
| KHK | ketohexokinase (fructokinase) | NM_006488 | 3795 | 1536443 | 34 | Inhibition (2/3) | Non-toxic | Viral helper |
| LATS2 | LATS, large tumor suppressor, homolog 2 (Drosophila) | NM_014572 | 26524 | 1903478 | 42 | Inhibition (2/3) | Non-toxic | Viral helper |
| MAGI2 | membrane associated guanylate kinase, WW and PDZ domain containing 2 | NM_012301 | 9863 | 1919881 | 43 | Inhibition (2/3) | Non-toxic | Viral helper |
| MAP2K7 | mitogen-activated protein kinase kinase 7 | NM_145185 | 5609 | 2279213 | 51 | Inhibition (2/3) | Non-toxic | Viral helper |
| MAP3K14 | mitogen-activated protein kinase kinase kinase 14 | NM_003954 | 9020 | 1912313 | 42 | Inhibition (2/3) | Non-toxic | Viral helper |
| MATK | megakaryocyte-associated tyrosine kinase | NM_002378 | 4145 | 1893939 | 42 | Inhibition (2/3) | Non-toxic | Viral helper |
| MINPP1 | multiple inositol polyphosphate histidine phosphatase, 1 | NM_004897 | 9562 | 1865987 | 41 | Inhibition (2/3) | Non-toxic | Viral helper |
| MPP7 | membrane protein, palmitoylated 7 (MAGUK p55 subfamily member 7) | NM_173496 | 143098 | 1873155 | 42 | Inhibition (2/3) | Non-toxic | Viral helper |
| MST1R | macrophage stimulating 1 receptor (c-met-related tyrosine kinase) | NM_002447 | 4486 | 2384937 | 53 | Inhibition (2/3) | Non-toxic | Viral helper |
| NEK4 | NIMA (never in mitosis gene a)-related kinase 4 | NM_003157 | 6787 | 2757646 | 61 | Inhibition (2/3) | Non-toxic | Viral helper |
| NRBP2 | nuclear receptor binding protein 2 | NM_178564 | 340371 | 2268354 | 50 | Inhibition (2/3) | Non-toxic | Viral helper |
| PAK6 | p21 protein (Cdc42/Rac)-activated kinase 6 | NM_020168 | 56924 | 589450 | 13 | Inhibition (2/3) | Non-toxic | Viral helper |
| PDK4 | pyruvate dehydrogenase kinase, isozyme 4 | NM_002612 | 5166 | 251114 | 6 | Inhibition (2/3) | Non-toxic | Viral helper |
| PFKFB1 | 6-phosphofructo-2-kinase/fructose-2,6-biphosphatase 1 | NM_002625 | 5207 | 1005379 | 22 | Inhibition (2/3) | Non-toxic | Viral helper |
| PIK3C2G | phosphoinositide-3-kinase, class 2, gamma polypeptide | NM_004570 | 5288 | 2555584 | 57 | Inhibition (2/3) | Non-toxic | Viral helper |
| PIP5K1C | phosphatidylinositol-4-phosphate 5-kinase, type I, gamma | NM_012398 | 23396 | 1933840 | 43 | Inhibition (2/3) | Non-toxic | Viral helper |
| PKLR | pyruvate kinase, liver and RBC | NM_181871 | 5313 | 2146746 | 48 | Inhibition (2/3) | Non-toxic | Viral helper |
| PLK4 | polo-like kinase 4 (Drosophila) | NM_014264 | 10733 | 1663736 | 37 | Inhibition (2/3) | Non-toxic | Viral helper |
| PLXNB2 | plexin B2 | XM_371474 | 23654 | 2088260 | 46 | Inhibition (2/3) | Non-toxic | Viral helper |
| PPM1A | protein phosphatase 1A (formerly 2C), magnesium-dependent, alpha isoform | NM_177951 | 5494 | 1974978 | 44 | Inhibition (2/3) | Non-toxic | Viral helper |
| PPP1R12C | protein phosphatase 1, regulatory subunit 12C | NM_017607 | 54776 | 2324349 | 52 | Inhibition (2/3) | Non-toxic | Viral helper |
| PPP1R14D | protein phosphatase 1, regulatory (inhibitor) subunit 14D | NM_017726 | 54866 | 2225583 | 49 | Inhibition (2/3) | Non-toxic | Viral helper |
| PPP1R2 | protein phosphatase 1, regulatory (inhibitor) subunit 2 | NM_006241 | 5504 | 2090356 | 46 | Inhibition (2/3) | Non-toxic | Viral helper |
| PPP1R7 | protein phosphatase 1, regulatory subunit 7 | NM_002712 | 5510 | 3020584 | 67 | Inhibition (2/3) | Non-toxic | Viral helper |
| PPP2CA | protein phosphatase 2, catalytic subunit, alpha isozyme | NM_002715 | 5515 | 1377381 | 31 | Inhibition (2/3) | Non-toxic | Viral helper |
| PRKD3 | protein kinase D3 | NM_005813 | 23683 | 2188261 | 49 | Inhibition (2/3) | Non-toxic | Viral helper |
| PRPF4B | PRP4 pre-mRNA processing factor 4 homolog B | NM_003913 | 8899 | 1927450 | 43 | Inhibition (3/3) | Non-toxic | Viral helper |
| PTPRH | protein tyrosine phosphatase, receptor type, H | NM_002842 | 5794 | 2102127 | 47 | Inhibition (2/3) | Non-toxic | Viral helper |
| PXK | PX domain containing serine/threonine kinase | NM_017771 | 54899 | 211663 | 5 | Inhibition (2/3) | Non-toxic | Viral helper |
| RIOK2 | RIO kinase 2 (yeast) | NM_018343 | 55781 | 1472591 | 33 | Inhibition (2/3) | Non-toxic | Viral helper |
| RNGTT | RNA guanylyltransferase and 5'-phosphatase | NM_003800 | 8732 | 526461 | 12 | Inhibition (2/3) | Non-toxic | Viral helper |
| RPS6KA2 | ribosomal protein S6 kinase, 90kDa, polypeptide 2 | NM_001006932 | 6196 | 1700318 | 38 | Inhibition (2/3) | Non-toxic | Viral helper |
| RPS6KA5 | ribosomal protein S6 kinase, 90kDa, polypeptide 5 | NM_004755 | 9252 | 689086 | 15 | Inhibition (2/3) | Non-toxic | Viral helper |
| RWDD2B | RWD domain containing 2B | NM_016940 | 10069 | 2228746 | 50 | Inhibition (2/3) | Non-toxic | Viral helper |
| SCYL1 | SCY1-like 1 (S. cerevisiae) | NM_001048218 | 57410 | 1593150 | 35 | Inhibition (2/3) | Non-toxic | Viral helper |
| STRADB | STE20-related kinase adaptor beta | NM_018571 | 55437 | 2340319 | 52 | Inhibition (2/3) | Non-toxic | Viral helper |
| TLK1 | tousled-like kinase 1 | NM_012290 | 9874 | 1844202 | 41 | Inhibition (2/3) | Non-toxic | Viral helper |
| TRIB2 | tribbles homolog 2 (Drosophila) | NM_021643 | 28951 | 952306 | 21 | Inhibition (2/3) | Non-toxic | Viral helper |
| TSKS | testis-specific serine kinase substrate | NM_021733 | 60385 | 986981 | 22 | Inhibition (2/3) | Non-toxic | Viral helper |
| TTBK2 | tau tubulin kinase 2 | NM_173500 | 146057 | 2866957 | 64 | Inhibition (2/3) | Non-toxic | Viral helper |
| TTK | TTK protein kinase | NM_003318 | 7272 | 1239442 | 28 | Inhibition (2/3) | Non-toxic | Viral helper |
| UCK1 | uridine-cytidine kinase 1 | NM_031432 | 83549 | 1559340 | 35 | Inhibition (2/3) | Non-toxic | Viral helper |
| WNK2 | WNK lysine deficient protein kinase 2 | NM_006648 | 65268 | 1797141 | 40 | Inhibition (2/3) | Non-toxic | Viral helper |
| FYN | FYN oncogene related to SRC, FGR, YES | NM_002037 | 2534 | 7441366 | 165 | Activator (2/3) | Non-toxic | Viral inhibitor |
| MTM1 | myotubularin 1 | NM_000252 | 4534 | 7718965 | 172 | Activator (2/3) | Non-toxic | Viral inhibitor |
| PPP1CC | protein phosphatase 1, catalytic subunit, gamma isoform | NM_002710 | 5501 | 7759759 | 172 | Activator (2/3) | Non-toxic | Viral inhibitor |
| PTPN1 | protein tyrosine phosphatase, non-receptor type 1 | NM_002827 | 5770 | 7532909 | 167 | Activator (2/3) | Non-toxic | Viral inhibitor |
| PTPN2 | protein tyrosine phosphatase, non-receptor type 2 | NM_002828 | 5771 | 7805315 | 173 | Activator (2/3) | Non-toxic | Viral inhibitor |
| PTPN7 | protein tyrosine phosphatase, non-receptor type 7 | NM_002832 | 5778 | 7681547 | 171 | Activator (2/3) | Non-toxic | Viral inhibitor |
| PTPN9 | protein tyrosine phosphatase, non-receptor type 9 | NM_002833 | 5780 | 7620918 | 169 | Activator (2/3) | Non-toxic | Viral inhibitor |
| AAK1 | AP2 associated kinase 1 | NM_014911 | 22848 | 2762612 | 61 | Inhibition (1/3) | Non-Toxic | ? |
| AATK | apoptosis-associated tyrosine kinase | NM_001080395 | 9625 | 1793764 | 40 | Inhibition (1/3 | Non-Toxic | ? |
| ABL1 | c-abl oncogene 1, non-receptor tyrosine kinase | NM_007313 | 25 | 1807483 | 40 | Inhibition (1/3 | Non-Toxic | ? |
| ACPL2 | acid phosphatase-like 2 | NM_001037172 | 92370 | 3144416 | 70 | Inhibition (1/3 | Non-Toxic | ? |
| ADCK2 | aarF domain containing kinase 2 | NM_052853 | 90956 | 3041132 | 68 | Inhibition (1/3 | Non-Toxic | ? |
| ADCK4 | aarF domain containing kinase 4 | NM_024876 | 79934 | 915785 | 20 | Inhibition (1/3 | Non-Toxic | ? |
| ADCK5 | aarF domain containing kinase 5 | NM_174922 | 203054 | 3141416 | 70 | Inhibition (1/3 | Non-Toxic | ? |
| ADPGK | ADP-dependent glucokinase | NM_031284 | 83440 | 2428376 | 54 | Inhibition (1/3 | Non-Toxic | ? |
| AGK | acylglycerol kinase | NM_018238 | 55750 | 2645919 | 59 | Inhibition (1/3 | Non-Toxic | ? |
| AK8 | adenylate kinase 8 | NM_152572 | 158067 | 2424449 | 54 | Inhibition (1/3 | Non-Toxic | ? |
| AKAP12 | A kinase (PRKA) anchor protein 12 | NM_005100 | 9590 | 2695778 | 60 | Inhibition (1/3 | Non-Toxic | ? |
| AKAP7 | A kinase (PRKA) anchor protein 7 | NM_004842 | 9465 | 1346446 | 30 | Inhibition (1/3 | Non-Toxic | ? |
| AKAP8 | A kinase (PRKA) anchor protein 8 | NM_005858 | 10270 | 3114646 | 69 | Inhibition (1/3 | Non-Toxic | ? |
| AKT2 | v-akt murine thymoma viral oncogene homolog 2 | NM_001626 | 208 | 1378563 | 31 | Inhibition (1/3 | Non-Toxic | ? |
| ALK | anaplastic lymphoma receptor tyrosine kinase | NM_004304 | 238 | 2747825 | 61 | Inhibition (1/3 | Non-Toxic | ? |
| ALPK3 | alpha-kinase 3 | NM_020778 | 57538 | 1998396 | 44 | Inhibition (1/3 | Non-Toxic | ? |
| ALPL | alkaline phosphatase, liver/bone/kidney | NM_000478 | 249 | 429476 | 10 | Inhibition (1/3 | Non-Toxic | ? |
| ALPP | alkaline phosphatase, placental | NM_001632 | 250 | 2862310 | 64 | Inhibition (1/3 | Non-Toxic | ? |
| ALPPL2 | alkaline phosphatase, placental-like 2 | NM_031313 | 251 | 3043494 | 68 | Inhibition (1/3 | Non-Toxic | ? |
| AURKAIP1 | aurora kinase A interacting protein 1 | NM_017900 | 54998 | 2318911 | 52 | Inhibition (1/3 | Non-Toxic | ? |
| BCKDK | branched chain ketoacid dehydrogenase kinase | NM_005881 | 10295 | 1730146 | 38 | Inhibition (1/3 | Non-Toxic | ? |
| BMP2K | BMP2 inducible kinase | NM_017593 | 55589 | 3049636 | 68 | Inhibition (1/3 | Non-Toxic | ? |
| CAMKK2 | calcium/calmodulin-dependent protein kinase kinase 2, beta | NM_006549 | 10645 | 2832811 | 63 | Inhibition (1/3 | Non-Toxic | ? |
| CCDC104 | coiled-coil domain containing 104 | NM_080667 | 112942 | 2758392 | 61 | Inhibition (1/3 | Non-Toxic | ? |
| CDC14B | CDC14 cell division cycle 14 homolog B (S. cerevisiae) | NM_001077181 | 8555 | 2430226 | 54 | Inhibition (1/3 | Non-Toxic | ? |
| CDC42SE2 | CDC42 small effector 2 | NM_001038702 | 56990 | 2080341 | 46 | Inhibition (1/3 | Non-Toxic | ? |
| CDK14 | cyclin-dependent kinase 14 | NM_012395 | 5218 | 1290324 | 29 | Inhibition (1/3 | Non-Toxic | ? |
| CDK15 | cyclin-dependent kinase 15 | NM_139158 | 65061 | 2362355 | 52 | Inhibition (1/3 | Non-Toxic | ? |
| CDK4 | cyclin-dependent kinase 4 | NM_000075 | 1019 | 1692889 | 38 | Inhibition (1/3 | Non-Toxic | ? |
| CDK7 | cyclin-dependent kinase 7 | NM_001799 | 1022 | 2197740 | 49 | Inhibition (1/3 | Non-Toxic | ? |
| CDK8 | cyclin-dependent kinase 8 | NM_001260 | 1024 | 1694544 | 38 | Inhibition (1/3 | Non-Toxic | ? |
| CDKN3 | cyclin-dependent kinase inhibitor 3 | NM_005192 | 1033 | 2385047 | 53 | Inhibition (1/3 | Non-Toxic | ? |
| CHP2 | calcineurin B homologous protein 2 | NM_022097 | 63928 | 306017 | 7 | Inhibition (1/3 | Non-Toxic | ? |
| CIB1 | calcium and integrin binding 1 (calmyrin) | NM_006384 | 10519 | 1027072 | 23 | Inhibition (1/3 | Non-Toxic | ? |
| CSNK1D | casein kinase 1, delta | NM_001893 | 1453 | 2071542 | 46 | Inhibition (1/3 | Non-Toxic | ? |
| CTDP1 | CTD (carboxy-terminal domain, RNA polymerase II, polypeptide A) phosphatase, subunit 1 | NM_004715 | 9150 | 2940037 | 65 | Inhibition (1/3 | Non-Toxic | ? |
| DCLK3 | doublecortin-like kinase 3 | XM_940612 | 85443 | 1604751 | 36 | Inhibition (1/3 | Non-Toxic | ? |
| DGKA | diacylglycerol kinase, alpha 80kDa | NM_001345 | 1606 | 2701878 | 60 | Inhibition (1/3 | Non-Toxic | ? |
| DGKH | diacylglycerol kinase, eta | NM_152910 | 160851 | 1711176 | 38 | Inhibition (1/3 | Non-Toxic | ? |
| DGKI | diacylglycerol kinase, iota | NM_004717 | 9162 | 1404940 | 31 | Inhibition (1/3 | Non-Toxic | ? |
| DGKQ | diacylglycerol kinase, theta 110kDa | NM_001347 | 1609 | 1800811 | 40 | Inhibition (1/3 | Non-Toxic | ? |
| DGKZ | diacylglycerol kinase, zeta 104kDa | NM_201532 | 8525 | 2895321 | 64 | Inhibition (1/3 | Non-Toxic | ? |
| DGUOK | deoxyguanosine kinase | NM_080916 | 1716 | 1456504 | 32 | Inhibition (1/3 | Non-Toxic | ? |
| DMPK | dystrophia myotonica-protein kinase | NM_001081560 | 1760 | 194776 | 4 | Inhibition (1/3 | Non-Toxic | ? |
| DOK1 | docking protein 1, 62kDa (downstream of tyrosine kinase 1) | NM_001381 | 1796 | 2610028 | 58 | Inhibition (1/3 | Non-Toxic | ? |
| DOLPP1 | dolichyl pyrophosphate phosphatase 1 | NM_020438 | 57171 | 2940443 | 65 | Inhibition (1/3 | Non-Toxic | ? |
| DSTYK | dual serine/threonine and tyrosine protein kinase | NM_015375 | 25778 | 1452557 | 32 | Inhibition (1/3 | Non-Toxic | ? |
| DUSP10 | dual specificity phosphatase 10 | NM_007207 | 11221 | 2767629 | 62 | Inhibition (1/3 | Non-Toxic | ? |
| DUSP16 | dual specificity phosphatase 16 | NM_030640 | 80824 | 2914818 | 65 | Inhibition (1/3 | Non-Toxic | ? |
| DUSP18 | dual specificity phosphatase 18 | NM_152511 | 150290 | 1579518 | 35 | Inhibition (1/3 | Non-Toxic | ? |
| DUSP19 | dual specificity phosphatase 19 | NM_080876 | 142679 | 2106369 | 47 | Inhibition (1/3 | Non-Toxic | ? |
| DUSP4 | dual specificity phosphatase 4 | NM_001394 | 1846 | 1482523 | 33 | Inhibition (1/3 | Non-Toxic | ? |
| DUSP8 | dual specificity phosphatase 8 | NM_004420 | 1850 | 2312228 | 51 | Inhibition (1/3 | Non-Toxic | ? |
| DUSP9 | dual specificity phosphatase 9 | NM_001395 | 1852 | 2688979 | 60 | Inhibition (1/3 | Non-Toxic | ? |
| DUT | deoxyuridine triphosphatase | NM_001025248 | 1854 | 2383043 | 53 | Inhibition (1/3 | Non-Toxic | ? |
| DYRK1A | dual-specificity tyrosine-(Y)-phosphorylation regulated kinase 1A | NM_001396 | 1859 | 2601902 | 58 | Inhibition (1/3 | Non-Toxic | ? |
| DYRK4 | dual-specificity tyrosine-(Y)-phosphorylation regulated kinase 4 | NM_003845 | 8798 | 2635662 | 59 | Inhibition (1/3 | Non-Toxic | ? |
| EIF2AK1 | eukaryotic translation initiation factor 2-alpha kinase 1 | NM_014413 | 27102 | 1384121 | 31 | Inhibition (1/3 | Non-Toxic | ? |
| EIF2AK3 | eukaryotic translation initiation factor 2-alpha kinase 3 | NM_004836 | 9451 | 2575562 | 57 | Inhibition (1/3 | Non-Toxic | ? |
| ENOPH1 | enolase-phosphatase 1 | NM_021204 | 58478 | 1387476 | 31 | Inhibition (1/3 | Non-Toxic | ? |
| ENTPD1 | ectonucleoside triphosphate diphosphohydrolase 1 | NM_001776 | 953 | 2619399 | 58 | Inhibition (1/3 | Non-Toxic | ? |
| ENTPD5 | ectonucleoside triphosphate diphosphohydrolase 5 | NM_001249 | 957 | 3021854 | 67 | Inhibition (1/3 | Non-Toxic | ? |
| EPHB4 | EPH receptor B4 | NM_004444 | 2050 | 2420568 | 54 | Inhibition (1/3 | Non-Toxic | ? |
| EPHB6 | EPH receptor B6 | NM_004445 | 2051 | 2357661 | 52 | Inhibition (1/3 | Non-Toxic | ? |
| ERBB2 | v-erb-b2 erythroblastic leukemia viral oncogene homolog 2, neuro/glioblastoma derived oncogene homolog (avian) | NM_001005862 | 2064 | 1799556 | 40 | Inhibition (1/3 | Non-Toxic | ? |
| FASTK | Fas-activated serine/threonine kinase | NM_006712 | 10922 | 3061904 | 68 | Inhibition (1/3 | Non-Toxic | ? |
| FES | feline sarcoma oncogene | NM_002005 | 2242 | 1664862 | 37 | Inhibition (1/3 | Non-Toxic | ? |
| FGGY | FGGY carbohydrate kinase domain containing | NM_018291 | 55277 | 2983682 | 66 | Inhibition (1/3 | Non-Toxic | ? |
| FRK | fyn-related kinase | NM_002031 | 2444 | 2787384 | 62 | Inhibition (1/3 | Non-Toxic | ? |
| G6PC3 | glucose 6 phosphatase, catalytic, 3 | NM_138387 | 92579 | 3107289 | 69 | Inhibition (1/3 | Non-Toxic | ? |
| HINT2 | histidine triad nucleotide binding protein 2 | NM_032593 | 84681 | 1471144 | 33 | Inhibition (1/3 | Non-Toxic | ? |
| HIPK4 | homeodomain interacting protein kinase 4 | NM_144685 | 147746 | 707864 | 16 | Inhibition (1/3 | Non-Toxic | ? |
| HK2 | hexokinase 2 | NM_000189 | 3099 | 3042495 | 68 | Inhibition (1/3 | Non-Toxic | ? |
| IGF1R | insulin-like growth factor 1 receptor | NM_000875 | 3480 | 2440933 | 54 | Inhibition (1/3 | Non-Toxic | ? |
| IGSF22 | immunoglobulin superfamily, member 22 | NM_173588 | 283284 | 2637430 | 59 | Inhibition (1/3 | Non-Toxic | ? |
| IKBKE | inhibitor of kappa light polypeptide gene enhancer in B-cells, kinase epsilon | NM_014002 | 9641 | 2774537 | 62 | Inhibition (1/3 | Non-Toxic | ? |
| INPP5J | inositol polyphosphate-5-phosphatase J | NM_001002837 | 27124 | 2592023 | 58 | Inhibition (1/3 | Non-Toxic | ? |
| INPP5K | inositol polyphosphate-5-phosphatase K | NM_130766 | 51763 | 1009804 | 22 | Inhibition (1/3 | Non-Toxic | ? |
| INPPL1 | inositol polyphosphate phosphatase-like 1 | NM_001567 | 3636 | 3090825 | 69 | Inhibition (1/3 | Non-Toxic | ? |
| IP6K3 | inositol hexaphosphate kinase 3 | NM_054111 | 117283 | 1123054 | 25 | Inhibition (1/3 | Non-Toxic | ? |
| IPPK | inositol 1,3,4,5,6-pentakisphosphate 2-kinase | NM_022755 | 64768 | 2922603 | 65 | Inhibition (1/3 | Non-Toxic | ? |
| ITGB1BP3 | integrin beta 1 binding protein 3 | NM_014446 | 27231 | 1475601 | 33 | Inhibition (1/3 | Non-Toxic | ? |
| ITK | IL2-inducible T-cell kinase | NM_005546 | 3702 | 975905 | 22 | Inhibition (1/3 | Non-Toxic | ? |
| JAK1 | Janus kinase 1 | NM_002227 | 3716 | 1150433 | 26 | Inhibition (1/3 | Non-Toxic | ? |
| KDR | kinase insert domain receptor (a type III receptor tyrosine kinase) | NM_002253 | 3791 | 2332319 | 52 | Inhibition (1/3 | Non-Toxic | ? |
| KSR1 | kinase suppressor of ras 1 | NM_014238 | 8844 | 891516 | 20 | Inhibition (1/3 | Non-Toxic | ? |
| KSR2 | kinase suppressor of ras 2 | NM_173598 | 283455 | 2656006 | 59 | Inhibition (1/3 | Non-Toxic | ? |
| LIMK2 | LIM domain kinase 2 | NM_001031801 | 3985 | 2981685 | 66 | Inhibition (1/3 | Non-Toxic | ? |
| LMTK2 | lemur tyrosine kinase 2 | NM_014916 | 22853 | 1513446 | 34 | Inhibition (1/3 | Non-Toxic | ? |
| LPPR1 | lipid phosphate phosphatase-related protein type 1 | NM_017753 | 54886 | 950131 | 21 | Inhibition (1/3 | Non-Toxic | ? |
| LRGUK | leucine-rich repeats and guanylate kinase domain containing | NM_144648 | 136332 | 1910220 | 42 | Inhibition (1/3 | Non-Toxic | ? |
| MAP2K1 | mitogen-activated protein kinase kinase 1 | NM_002755 | 5604 | 1618036 | 36 | Inhibition (1/3 | Non-Toxic | ? |
| MAP2K4 | mitogen-activated protein kinase kinase 4 | NM_003010 | 6416 | 2032936 | 45 | Inhibition (1/3 | Non-Toxic | ? |
| MAP2K5 | mitogen-activated protein kinase kinase 5 | NM_002757 | 5607 | 2095399 | 47 | Inhibition (1/3 | Non-Toxic | ? |
| MAP3K10 | mitogen-activated protein kinase kinase kinase 10 | NM_002446 | 4294 | 859850 | 19 | Inhibition (1/3 | Non-Toxic | ? |
| MAP3K2 | mitogen-activated protein kinase kinase kinase 2 | NM_006609 | 10746 | 2323829 | 52 | Inhibition (1/3 | Non-Toxic | ? |
| MAP3K6 | mitogen-activated protein kinase kinase kinase 6 | NM_004672 | 9064 | 2301908 | 51 | Inhibition (1/3 | Non-Toxic | ? |
| MAP3K7 | mitogen-activated protein kinase kinase kinase 7 | NM_003188 | 6885 | 2995195 | 67 | Inhibition (1/3 | Non-Toxic | ? |
| MAP4K2 | mitogen-activated protein kinase kinase kinase kinase 2 | NM_004579 | 5871 | 1801430 | 40 | Inhibition (1/3 | Non-Toxic | ? |
| MAP4K3 | mitogen-activated protein kinase kinase kinase kinase 3 | NM_003618 | 8491 | 2080913 | 46 | Inhibition (1/3 | Non-Toxic | ? |
| MAPK11 | mitogen-activated protein kinase 11 | NM_002751 | 5600 | 2861640 | 64 | Inhibition (1/3 | Non-Toxic | ? |
| MAPK12 | mitogen-activated protein kinase 12 | NM_002969 | 6300 | 2692946 | 60 | Inhibition (1/3 | Non-Toxic | ? |
| MAPK3 | mitogen-activated protein kinase 3 | NM_001040056 | 5595 | 1113639 | 25 | Inhibition (1/3 | Non-Toxic | ? |
| MAPK7 | mitogen-activated protein kinase 7 | NM_139034 | 5598 | 2681870 | 60 | Inhibition (1/3 | Non-Toxic | ? |
| MAPK8 | mitogen-activated protein kinase 8 | NM_002750 | 5599 | 3040405 | 68 | Inhibition (1/3 | Non-Toxic | ? |
| MAPKAPK5 | mitogen-activated protein kinase-activated protein kinase 5 | NM_003668 | 8550 | 1601349 | 36 | Inhibition (1/3 | Non-Toxic | ? |
| MARK2 | MAP/microtubule affinity-regulating kinase 2 | NM_001039468 | 2011 | 1728795 | 38 | Inhibition (1/3 | Non-Toxic | ? |
| MARK3 | MAP/microtubule affinity-regulating kinase 3 | NM_002376 | 4140 | 2641707 | 59 | Inhibition (1/3 | Non-Toxic | ? |
| MFN1 | similar to mitofusin 1 | XM_937130 | 441511 | 1874311 | 42 | Inhibition (1/3 | Non-Toxic | ? |
| MFN1 | mitofusin 1 | NM_033540 | 55669 | 2830357 | 63 | Inhibition (1/3 | Non-Toxic | ? |
| MFN1 | similar to mitofusin 1 | XM_937130 | 441511 | 3149212 | 70 | Inhibition (1/3 | Non-Toxic | ? |
| MFN2 | mitofusin 2 | NM_014874 | 9927 | 2242356 | 50 | Inhibition (1/3 | Non-Toxic | ? |
| MKNK1 | MAP kinase interacting serine/threonine kinase 1 | NM_003684 | 8569 | 1993039 | 44 | Inhibition (1/3 | Non-Toxic | ? |
| MORN1 | MORN repeat containing 1 | NM_024848 | 79906 | 764154 | 17 | Inhibition (1/3 | Non-Toxic | ? |
| N/A | similar to template acyivating factor-I alpha | XM_371701 | 389217 | 2383245 | 53 | Inhibition (1/3 | Non-Toxic | ? |
| NAP1L5 | nucleosome assembly protein 1-like 5 | NM_153757 | 266812 | 2854337 | 63 | Inhibition (1/3 | Non-Toxic | ? |
| NME4 | non-metastatic cells 4, protein expressed in | NM_005009 | 4833 | 157959 | 4 | Inhibition (1/3 | Non-Toxic | ? |
| NRBP1 | nuclear receptor binding protein 1 | NM_013392 | 29959 | 3064549 | 68 | Inhibition (1/3 | Non-Toxic | ? |
| NT5C2 | 5'-nucleotidase, cytosolic II | NM_012229 | 22978 | 2868100 | 64 | Inhibition (1/3 | Non-Toxic | ? |
| NTRK1 | neurotrophic tyrosine kinase, receptor, type 1 | NM_001007792 | 4914 | 1572146 | 35 | Inhibition (1/3 | Non-Toxic | ? |
| NUAK2 | NUAK family, SNF1-like kinase, 2 | NM_030952 | 81788 | 2227595 | 50 | Inhibition (1/3 | Non-Toxic | ? |
| NUDT11 | nudix (nucleoside diphosphate linked moiety X)-type motif 11 | NM_018159 | 55190 | 2717345 | 60 | Inhibition (1/3 | Non-Toxic | ? |
| NUDT5 | nudix (nucleoside diphosphate linked moiety X)-type motif 5 | NM_014142 | 11164 | 1560153 | 35 | Inhibition (1/3 | Non-Toxic | ? |
| NUDT8 | nudix (nucleoside diphosphate linked moiety X)-type motif 8 | NM_181843 | 254552 | 3047922 | 68 | Inhibition (1/3 | Non-Toxic | ? |
| PANK1 | pantothenate kinase 1 | NM_138316 | 53354 | 936712 | 21 | Inhibition (1/3 | Non-Toxic | ? |
| PBK | PDZ binding kinase | NM_018492 | 55872 | 1774729 | 39 | Inhibition (1/3 | Non-Toxic | ? |
| PCTK2 | PCTAIRE protein kinase 2 | NM_002595 | 5128 | 1886405 | 42 | Inhibition (1/3 | Non-Toxic | ? |
| PDXK | pyridoxal (pyridoxine, vitamin B6) kinase | NM_003681 | 8566 | 2696048 | 60 | Inhibition (1/3 | Non-Toxic | ? |
| PDXP | pyridoxal (pyridoxine, vitamin B6) phosphatase | NM_020315 | 57026 | 2541951 | 56 | Inhibition (1/3 | Non-Toxic | ? |
| PFKM | phosphofructokinase, muscle | NM_000289 | 5213 | 573736 | 13 | Inhibition (1/3 | Non-Toxic | ? |
| PGK1 | phosphoglycerate kinase 1 | NM_000291 | 5230 | 1292808 | 29 | Inhibition (1/3 | Non-Toxic | ? |
| PHACTR1 | phosphatase and actin regulator 1 | NM_030948 | 221692 | 1905275 | 42 | Inhibition (1/3 | Non-Toxic | ? |
| PHKG2 | phosphorylase kinase, gamma 2 (testis) | NM_000294 | 5261 | 1693878 | 38 | Inhibition (1/3 | Non-Toxic | ? |
| PI4K2A | phosphatidylinositol 4-kinase type 2 alpha | NM_018425 | 55361 | 1233262 | 27 | Inhibition (1/3 | Non-Toxic | ? |
| PI4K2B | phosphatidylinositol 4-kinase type 2 beta | NM_018323 | 55300 | 2063717 | 46 | Inhibition (1/3 | Non-Toxic | ? |
| PICK1 | protein interacting with PRKCA 1 | NM_001039583 | 9463 | 1910365 | 42 | Inhibition (1/3 | Non-Toxic | ? |
| PIK3AP1 | phosphoinositide-3-kinase adaptor protein 1 | NM_152309 | 118788 | 2720277 | 60 | Inhibition (1/3 | Non-Toxic | ? |
| PIK3C2B | phosphoinositide-3-kinase, class 2, beta polypeptide | NM_002646 | 5287 | 2596368 | 58 | Inhibition (1/3 | Non-Toxic | ? |
| PIK3R3 | phosphoinositide-3-kinase, regulatory subunit 3 (gamma) | NM_003629 | 8503 | 3151826 | 70 | Inhibition (1/3 | Non-Toxic | ? |
| PIM1 | pim-1 oncogene | NM_002648 | 5292 | 1459992 | 32 | Inhibition (1/3 | Non-Toxic | ? |
| PIP4K2B | phosphatidylinositol-5-phosphate 4-kinase, type II, beta | NM_003559 | 8396 | 1257998 | 28 | Inhibition (1/3 | Non-Toxic | ? |
| PIP5K1A | phosphatidylinositol-4-phosphate 5-kinase, type I, alpha | NM_003557 | 8394 | 3141660 | 70 | Inhibition (1/3 | Non-Toxic | ? |
| PKM2 | pyruvate kinase, muscle | NM_002654 | 5315 | 862765 | 19 | Inhibition (1/3 | Non-Toxic | ? |
| PKMYT1 | protein kinase, membrane associated tyrosine/threonine 1 | NM_004203 | 9088 | 1363533 | 30 | Inhibition (1/3 | Non-Toxic | ? |
| PKN1 | protein kinase N1 | NM_002741 | 5585 | 152971 | 3 | Inhibition (1/3 | Non-Toxic | ? |
| PLXNA2 | plexin A2 | NM_025179 | 5362 | 886321 | 20 | Inhibition (1/3 | Non-Toxic | ? |
| PLXNA4 | plexin A4 | NM_181775 | 91584 | 2534404 | 56 | Inhibition (1/3 | Non-Toxic | ? |
| PLXND1 | plexin D1 | NM_015103 | 23129 | 2876318 | 64 | Inhibition (1/3 | Non-Toxic | ? |
| PMVK | phosphomevalonate kinase | NM_006556 | 10654 | 479232 | 11 | Inhibition (1/3 | Non-Toxic | ? |
| PNCK | pregnancy up-regulated non-ubiquitously expressed CaM kinase | NM_001039582 | 139728 | 1901557 | 42 | Inhibition (1/3 | Non-Toxic | ? |
| PNKP | polynucleotide kinase 3'-phosphatase | NM_007254 | 11284 | 3052998 | 68 | Inhibition (1/3 | Non-Toxic | ? |
| PPAP2C | phosphatidic acid phosphatase type 2C | NM_003712 | 8612 | 2948209 | 66 | Inhibition (1/3 | Non-Toxic | ? |
| PPAPDC1A | phosphatidic acid phosphatase type 2 domain containing 1A | NM_001030059 | 196051 | 1832780 | 41 | Inhibition (1/3 | Non-Toxic | ? |
| PPM1G | protein phosphatase, Mg2+/Mn2+ dependent, 1G | NM_177983 | 5496 | 1578133 | 35 | Inhibition (1/3 | Non-Toxic | ? |
| PPM1J | protein phosphatase, Mg2+/Mn2+ dependent, 1J | NM_005167 | 333926 | 2010069 | 45 | Inhibition (1/3 | Non-Toxic | ? |
| PPM1K | protein phosphatase 1K (PP2C domain containing) | NM_152542 | 152926 | 2387124 | 53 | Inhibition (1/3 | Non-Toxic | ? |
| PPM1L | protein phosphatase, Mg2+/Mn2+ dependent, 1L | NM_139245 | 151742 | 2823095 | 63 | Inhibition (1/3 | Non-Toxic | ? |
| PPM1M | protein phosphatase 1M (PP2C domain containing) | NM_144641 | 132160 | 2392053 | 53 | Inhibition (1/3 | Non-Toxic | ? |
| PPP1R11 | protein phosphatase 1, regulatory (inhibitor) subunit 11 | NM_021959 | 6992 | 2732203 | 61 | Inhibition (1/3 | Non-Toxic | ? |
| PPP1R12B | protein phosphatase 1, regulatory (inhibitor) subunit 12B | NM_032104 | 4660 | 2193790 | 49 | Inhibition (1/3 | Non-Toxic | ? |
| PPP1R16B | protein phosphatase 1, regulatory subunit 16B | NM_015568 | 26051 | 2414823 | 54 | Inhibition (1/3 | Non-Toxic | ? |
| PPP1R1A | protein phosphatase 1, regulatory (inhibitor) subunit 1A | NM_006741 | 5502 | 1767493 | 39 | Inhibition (1/3 | Non-Toxic | ? |
| PPP1R1C | protein phosphatase 1, regulatory (inhibitor) subunit 1C | NM_001080545 | 151242 | 1761543 | 39 | Inhibition (1/3 | Non-Toxic | ? |
| PPP1R3C | protein phosphatase 1, regulatory (inhibitor) subunit 3C | NM_005398 | 5507 | 2648140 | 59 | Inhibition (1/3 | Non-Toxic | ? |
| PPP1R8 | protein phosphatase 1, regulatory (inhibitor) subunit 8 | NM_002713 | 5511 | 1167953 | 26 | Inhibition (1/3 | Non-Toxic | ? |
| PPP2R3B | protein phosphatase 2 (formerly 2A), regulatory subunit B'', beta | NM_013239 | 28227 | 2269264 | 50 | Inhibition (1/3 | Non-Toxic | ? |
| PPP2R4 | protein phosphatase 2A activator, regulatory subunit 4 | NM_021131 | 5524 | 1263502 | 28 | Inhibition (1/3 | Non-Toxic | ? |
| PPP2R5A | protein phosphatase 2, regulatory subunit B', alpha isoform | NM_006243 | 5525 | 1810741 | 40 | Inhibition (1/3 | Non-Toxic | ? |
| PRKAA2 | protein kinase, AMP-activated, alpha 2 catalytic subunit | NM_006252 | 5563 | 1244565 | 28 | Inhibition (1/3 | Non-Toxic | ? |
| PRKACG | protein kinase, cAMP-dependent, catalytic, gamma | NM_002732 | 5568 | 435442 | 10 | Inhibition (1/3 | Non-Toxic | ? |
| PRKCA | protein kinase C, alpha | NM_002737 | 5578 | 931990 | 21 | Inhibition (1/3 | Non-Toxic | ? |
| PRKCDBP | protein kinase C, delta binding protein | NM_145040 | 112464 | 372630 | 8 | Inhibition (1/3 | Non-Toxic | ? |
| PRKCG | protein kinase C, gamma | NM_002739 | 5582 | 2311094 | 51 | Inhibition (1/3 | Non-Toxic | ? |
| PTK2B | PTK2B protein tyrosine kinase 2 beta | NM_004103 | 2185 | 2844956 | 63 | Inhibition (1/3 | Non-Toxic | ? |
| PTK6 | PTK6 protein tyrosine kinase 6 | NM_005975 | 5753 | 1440840 | 32 | Inhibition (1/3 | Non-Toxic | ? |
| PTK7 | PTK7 protein tyrosine kinase 7 | NM_002821 | 5754 | 1559768 | 35 | Inhibition (1/3 | Non-Toxic | ? |
| PTP4A1 | protein tyrosine phosphatase type IVA, member 1 | NM_003463 | 7803 | 2846425 | 63 | Inhibition (1/3 | Non-Toxic | ? |
| PTP4A2 | protein tyrosine phosphatase type IVA, member 2 | NM_080391 | 8073 | 1030878 | 23 | Inhibition (1/3 | Non-Toxic | ? |
| PTPDC1 | protein tyrosine phosphatase domain containing 1 | NM_152422 | 138639 | 2707839 | 60 | Inhibition (1/3 | Non-Toxic | ? |
| PTPN11 | protein tyrosine phosphatase, non-receptor type 11 | NM_002834 | 5781 | 2130581 | 47 | Inhibition (1/3 | Non-Toxic | ? |
| PTPN20A | protein tyrosine phosphatase, non-receptor type 20A | NM_001042387 | 653129 | 2733691 | 61 | Inhibition (1/3 | Non-Toxic | ? |
| PTPN21 | protein tyrosine phosphatase, non-receptor type 21 | NM_007039 | 11099 | 2251562 | 50 | Inhibition (1/3 | Non-Toxic | ? |
| PTPN5 | protein tyrosine phosphatase, non-receptor type 5 (striatum-enriched) | NM_001039970 | 84867 | 541573 | 12 | Inhibition (1/3 | Non-Toxic | ? |
| PTPN6 | protein tyrosine phosphatase, non-receptor type 6 | NM_002831 | 5777 | 2587167 | 57 | Inhibition (1/3 | Non-Toxic | ? |
| PTPRD | protein tyrosine phosphatase, receptor type, D | NM_001040712 | 5789 | 1839124 | 41 | Inhibition (1/3 | Non-Toxic | ? |
| PTPRE | protein tyrosine phosphatase, receptor type, E | NM_130435 | 5791 | 2988741 | 66 | Inhibition (1/3 | Non-Toxic | ? |
| PTPRJ | protein tyrosine phosphatase, receptor type, J | NM_002843 | 5795 | 2648845 | 59 | Inhibition (1/3 | Non-Toxic | ? |
| PTPRR | protein tyrosine phosphatase, receptor type, R | NM_002849 | 5801 | 1546720 | 34 | Inhibition (1/3 | Non-Toxic | ? |
| PTPRS | protein tyrosine phosphatase, receptor type, S | NM_002850 | 5802 | 1496469 | 33 | Inhibition (1/3 | Non-Toxic | ? |
| PTPRT | protein tyrosine phosphatase, receptor type, T | NM_007050 | 11122 | 2669504 | 59 | Inhibition (1/3 | Non-Toxic | ? |
| PTPRZ1 | protein tyrosine phosphatase, receptor-type, Z polypeptide 1 | NM_002851 | 5803 | 2884660 | 64 | Inhibition (1/3 | Non-Toxic | ? |
| RAF1 | v-raf-1 murine leukemia viral oncogene homolog 1 | NM_002880 | 5894 | 3038340 | 68 | Inhibition (1/3 | Non-Toxic | ? |
| RIOK1 | RIO kinase 1 (yeast) | NM_031480 | 83732 | 1526452 | 34 | Inhibition (1/3 | Non-Toxic | ? |
| RIOK3 | RIO kinase 3 (yeast) | NM_003831 | 8780 | 1754043 | 39 | Inhibition (1/3 | Non-Toxic | ? |
| RIPK2 | receptor-interacting serine-threonine kinase 2 | NM_003821 | 8767 | 1340153 | 30 | Inhibition (1/3 | Non-Toxic | ? |
| ROCK1 | Rho-associated, coiled-coil containing protein kinase 1 | NM_005406 | 6093 | 2061804 | 46 | Inhibition (1/3 | Non-Toxic | ? |
| ROR1 | receptor tyrosine kinase-like orphan receptor 1 | NM_001083592 | 4919 | 2007535 | 45 | Inhibition (1/3 | Non-Toxic | ? |
| RPS6KA1 | ribosomal protein S6 kinase, 90kDa, polypeptide 1 | NM_001006665 | 6195 | 2680711 | 60 | Inhibition (1/3 | Non-Toxic | ? |
| RPS6KA4 | ribosomal protein S6 kinase, 90kDa, polypeptide 4 | NM_001006944 | 8986 | 2433160 | 54 | Inhibition (1/3 | Non-Toxic | ? |
| RPS6KL1 | ribosomal protein S6 kinase-like 1 | NM_031464 | 83694 | 2841754 | 63 | Inhibition (1/3 | Non-Toxic | ? |
| SAPS1 | SAPS domain family, member 1 | NM_014931 | 22870 | 2434987 | 54 | Inhibition (1/3 | Non-Toxic | ? |
| SBF1 | SET binding factor 1 | NM_002972 | 6305 | 581012 | 13 | Inhibition (1/3 | Non-Toxic | ? |
| SBF2 | SET binding factor 2 | NM_030962 | 81846 | 783186 | 17 | Inhibition (1/3 | Non-Toxic | ? |
| SCYL3 | SCY1-like 3 (S. cerevisiae) | NM_181093 | 57147 | 2473472 | 55 | Inhibition (1/3 | Non-Toxic | ? |
| SH3BP5 | SH3-domain binding protein 5 (BTK-associated) | NM_001018009 | 9467 | 1656997 | 37 | Inhibition (1/3 | Non-Toxic | ? |
| SH3BP5L | SH3-binding domain protein 5-like | NM_030645 | 80851 | 749134 | 17 | Inhibition (1/3 | Non-Toxic | ? |
| SHPK | sedoheptulokinase | NM_013276 | 23729 | 1999562 | 44 | Inhibition (1/3 | Non-Toxic | ? |
| SKAP1 | src kinase associated phosphoprotein 1 | NM_001075099 | 8631 | 2694793 | 60 | Inhibition (1/3 | Non-Toxic | ? |
| SNRK | SNF related kinase | NM_017719 | 54861 | 2116375 | 47 | Inhibition (1/3 | Non-Toxic | ? |
| SPHK1 | sphingosine kinase 1 | NM_182965 | 8877 | 1436115 | 32 | Inhibition (1/3 | Non-Toxic | ? |
| SSH2 | slingshot homolog 2 (Drosophila) | NM_033389 | 85464 | 2417334 | 54 | Inhibition (1/3 | Non-Toxic | ? |
| STK16 | serine/threonine kinase 16 | NM_001008910 | 8576 | 3088304 | 69 | Inhibition (1/3 | Non-Toxic | ? |
| STK3 | serine/threonine kinase 3 | NM_006281 | 6788 | 2718890 | 60 | Inhibition (1/3 | Non-Toxic | ? |
| STK32A | serine/threonine kinase 32A | NM_145001 | 202374 | 2990435 | 66 | Inhibition (1/3 | Non-Toxic | ? |
| STK33 | serine/threonine kinase 33 | NM_030906 | 65975 | 723625 | 16 | Inhibition (1/3 | Non-Toxic | ? |
| STK38 | serine/threonine kinase 38 | NM_007271 | 11329 | 2214447 | 49 | Inhibition (1/3 | Non-Toxic | ? |
| STK4 | serine/threonine kinase 4 | NM_006282 | 6789 | 643086 | 14 | Inhibition (1/3 | Non-Toxic | ? |
| STYK1 | serine/threonine/tyrosine kinase 1 | NM_018423 | 55359 | 2378805 | 53 | Inhibition (1/3 | Non-Toxic | ? |
| SYNJ2 | synaptojanin 2 | NM_003898 | 8871 | 1067598 | 24 | Inhibition (1/3 | Non-Toxic | ? |
| TAF1 | TAF1 RNA polymerase II, TATA box binding protein (TBP)-associated factor, 250kDa | NM_138923 | 6872 | 974878 | 22 | Inhibition (1/3 | Non-Toxic | ? |
| TBK1 | TANK-binding kinase 1 | NM_013254 | 29110 | 3074509 | 68 | Inhibition (1/3 | Non-Toxic | ? |
| TEK | TEK tyrosine kinase, endothelial | NM_000459 | 7010 | 1151988 | 26 | Inhibition (1/3 | Non-Toxic | ? |
| TEX14 | testis expressed 14 | NM_031272 | 56155 | 2975476 | 66 | Inhibition (1/3 | Non-Toxic | ? |
| TIE1 | tyrosine kinase with immunoglobulin-like and EGF-like domains 1 | NM_005424 | 7075 | 1329539 | 30 | Inhibition (1/3 | Non-Toxic | ? |
| TNK1 | tyrosine kinase, non-receptor, 1 | NM_003985 | 8711 | 2119196 | 47 | Inhibition (1/3 | Non-Toxic | ? |
| TNNI3K | TNNI3 interacting kinase | NM_015978 | 51086 | 2127596 | 47 | Inhibition (1/3 | Non-Toxic | ? |
| TPTE | transmembrane phosphatase with tensin homology | NM_199259 | 7179 | 2451201 | 54 | Inhibition (1/3 | Non-Toxic | ? |
| TRAF3IP3 | TRAF3 interacting protein 3 | NM_025228 | 80342 | 2615373 | 58 | Inhibition (1/3 | Non-Toxic | ? |
| TSSK6 | testis-specific serine kinase 6 | NM_032037 | 83983 | 3137095 | 70 | Inhibition (1/3 | Non-Toxic | ? |
| TTBK1 | tau tubulin kinase 1 | NM_032538 | 84630 | 2842494 | 63 | Inhibition (1/3 | Non-Toxic | ? |
| TWF1 | twinfilin, actin-binding protein, homolog 1 (Drosophila) | NM_002822 | 5756 | 2380524 | 53 | Inhibition (1/3 | Non-Toxic | ? |
| UHMK1 | U2AF homology motif (UHM) kinase 1 | NM_175866 | 127933 | 2888217 | 64 | Inhibition (1/3 | Non-Toxic | ? |
| WNK1 | WNK lysine deficient protein kinase 1 | NM_018979 | 65125 | 3031780 | 67 | Inhibition (1/3 | Non-Toxic | ? |
| ZAK | sterile alpha motif and leucine zipper containing kinase AZK | NM_016653 | 51776 | 2203479 | 49 | Inhibition (1/3 | Non-Toxic | ? |
| ACPP | acid phosphatase, prostate | NM_001099 | 55 | 2488784 | 55 | Inhibition (1/3 | Toxic | ? |
| ACVR2A | activin A receptor, type IIA | NM_001616 | 92 | 464780 | 10 | Inhibition (1/3 | Toxic | ? |
| ACYP1 | acylphosphatase 1, erythrocyte (common) type | NM_001107 | 97 | 1142413 | 25 | Inhibition (1/3 | Toxic | ? |
| AK5 | adenylate kinase 5 | NM_012093 | 26289 | 796467 | 18 | Inhibition (1/3 | Toxic | ? |
| ALPI | alkaline phosphatase, intestinal | NM_001631 | 248 | 1394917 | 31 | Inhibition (1/3 | Toxic | ? |
| AURKA | aurora kinase A | NM_003600 | 6790 | 681621 | 15 | Inhibition (1/3 | Toxic | ? |
| BMPR1A | bone morphogenetic protein receptor, type IA | NM_004329 | 657 | 1038232 | 23 | Inhibition (1/3 | Toxic | ? |
| BMX | BMX non-receptor tyrosine kinase | NM_001721 | 660 | 975131 | 22 | Inhibition (1/3 | Toxic | ? |
| BRSK2 | BR serine/threonine kinase 2 | NM_003957 | 9024 | 190748 | 4 | Inhibition (1/3 | Toxic | ? |
| CALM1 | calmodulin 1 (phosphorylase kinase, delta) | NM_006888 | 801 | 963736 | 21 | Inhibition (1/3 | Toxic | ? |
| CALM2 | calmodulin 2 (phosphorylase kinase, delta) | NM_001743 | 805 | 1533600 | 34 | Inhibition (1/3 | Toxic | ? |
| CAMK1D | calcium/calmodulin-dependent protein kinase ID | NM_020397 | 57118 | 307222 | 7 | Inhibition (1/3 | Toxic | ? |
| CAMK1G | calcium/calmodulin-dependent protein kinase IG | NM_020439 | 57172 | 518571 | 12 | Inhibition (1/3 | Toxic | ? |
| CDC2 | cell division cycle 2, G1 to S and G2 to M | NM_001786 | 983 | 2726377 | 61 | Inhibition (1/3 | Toxic | ? |
| CDC42BPB | CDC42 binding protein kinase beta (DMPK-like) | NM_006035 | 9578 | 299953 | 7 | Inhibition (1/3 | Toxic | ? |
| CDK19 | cyclin-dependent kinase 19 | NM_015076 | 23097 | 441184 | 10 | Inhibition (1/3 | Toxic | ? |
| CHKA | choline kinase alpha | NM_001277 | 1119 | 2936770 | 65 | Inhibition (1/3 | Toxic | ? |
| CLK1 | CDC-like kinase 1 | NM_004071 | 1195 | 2372247 | 53 | Inhibition (1/3 | Toxic | ? |
| CSNK1A1 | casein kinase 1, alpha 1 | NM_001025105 | 1452 | 252277 | 6 | Inhibition (1/3 | Toxic | ? |
| CSNK1G2 | casein kinase 1, gamma 2 | NM_001319 | 1455 | 1182975 | 26 | Inhibition (1/3 | Toxic | ? |
| CTDSP1 | CTD (carboxy-terminal domain, RNA polymerase II, polypeptide A) small phosphatase 1 | NM_021198 | 58190 | 417615 | 9 | Inhibition (1/3 | Toxic | ? |
| DDR1 | discoidin domain receptor tyrosine kinase 1 | NM_001954 | 780 | 1375105 | 31 | Inhibition (1/3 | Toxic | ? |
| DGKK | diacylglycerol kinase, kappa | NM_001013742 | 139189 | 81530 | 2 | Inhibition (1/3 | Toxic | ? |
| DUSP1 | dual specificity phosphatase 1 | NM_004417 | 1843 | 987834 | 22 | Inhibition (1/3 | Toxic | ? |
| DUSP7 | dual specificity phosphatase 7 | NM_001947 | 1849 | 882912 | 20 | Inhibition (1/3 | Toxic | ? |
| ENTPD2 | ectonucleoside triphosphate diphosphohydrolase 2 | NM_001246 | 954 | 1889937 | 42 | Inhibition (1/3 | Toxic | ? |
| EPHA7 | EPH receptor A7 | NM_004440 | 2045 | 122756 | 3 | Inhibition (1/3 | Toxic | ? |
| FGFR3 | fibroblast growth factor receptor 3 | NM_000142 | 2261 | 374061 | 8 | Inhibition (1/3 | Toxic | ? |
| FGFR4 | fibroblast growth factor receptor 4 | NM_002011 | 2264 | 735659 | 16 | Inhibition (1/3 | Toxic | ? |
| FIG4 | FIG4 homolog (S. cerevisiae) | NM_014845 | 9896 | 833308 | 19 | Inhibition (1/3 | Toxic | ? |
| GALK2 | galactokinase 2 | NM_001001556 | 2585 | 814485 | 18 | Inhibition (1/3 | Toxic | ? |
| HDDC3 | HD domain containing 3 | NM_198527 | 374659 | 405236 | 9 | Inhibition (1/3 | Toxic | ? |
| HDHD2 | haloacid dehalogenase-like hydrolase domain containing 2 | NM_032124 | 84064 | 348802 | 8 | Inhibition (1/3 | Toxic | ? |
| ICK | intestinal cell (MAK-like) kinase | NM_014920 | 22858 | 59748 | 1 | Inhibition (1/3 | Toxic | ? |
| INSRR | insulin receptor-related receptor | NM_014215 | 3645 | 1143390 | 25 | Inhibition (1/3 | Toxic | ? |
| LY6G5B | lymphocyte antigen 6 complex, locus G5B | NM_021221 | 58496 | 831439 | 18 | Inhibition (1/3 | Toxic | ? |
| LYN | v-yes-1 Yamaguchi sarcoma viral related oncogene homolog | NM_002350 | 4067 | 996142 | 22 | Inhibition (1/3 | Toxic | ? |
| MAGI1 | membrane associated guanylate kinase, WW and PDZ domain containing 1 | NM_001033057 | 9223 | 678108 | 15 | Inhibition (1/3 | Toxic | ? |
| MAP3K11 | mitogen-activated protein kinase kinase kinase 11 | NM_002419 | 4296 | 169862 | 4 | Inhibition (1/3 | Toxic | ? |
| MAP3K5 | mitogen-activated protein kinase kinase kinase 5 | NM_005923 | 4217 | 597484 | 13 | Inhibition (1/3 | Toxic | ? |
| MAPK1 | mitogen-activated protein kinase 1 | NM_002745 | 5594 | 247425 | 5 | Inhibition (1/3 | Toxic | ? |
| MARK4 | MAP/microtubule affinity-regulating kinase 4 | NM_031417 | 57787 | 1289273 | 29 | Inhibition (1/3 | Toxic | ? |
| MPP1 | membrane protein, palmitoylated 1, 55kDa | NM_002436 | 4354 | 185037 | 4 | Inhibition (1/3 | Toxic | ? |
| MPP5 | membrane protein, palmitoylated 5 (MAGUK p55 subfamily member 5) | NM_022474 | 64398 | 560450 | 12 | Inhibition (1/3 | Toxic | ? |
| MRC2 | mannose receptor, C type 2 | NM_006039 | 9902 | 329031 | 7 | Inhibition (1/3 | Toxic | ? |
| MTMR4 | myotubularin related protein 4 | NM_004687 | 9110 | 583354 | 13 | Inhibition (1/3 | Toxic | ? |
| MTMR6 | myotubularin related protein 6 | NM_004685 | 9107 | 717740 | 16 | Inhibition (1/3 | Toxic | ? |
| MTMR8 | myotubularin related protein 8 | NM_017677 | 55613 | 594735 | 13 | Inhibition (1/3 | Toxic | ? |
| MVK | mevalonate kinase | NM_000431 | 4598 | 469579 | 10 | Inhibition (1/3 | Toxic | ? |
| MYLK3 | myosin light chain kinase 3 | NM_182493 | 91807 | 298810 | 7 | Inhibition (1/3 | Toxic | ? |
| NEK7 | NIMA (never in mitosis gene a)-related kinase 7 | NM_133494 | 140609 | 1455476 | 32 | Inhibition (1/3 | Toxic | ? |
| NUDT4 | nudix (nucleoside diphosphate linked moiety X)-type motif 4 | NM_019094 | 11163 | 2747847 | 61 | Inhibition (1/3 | Toxic | ? |
| PAK4 | p21 protein (Cdc42/Rac)-activated kinase 4 | NM_001014831 | 10298 | 1333299 | 30 | Inhibition (1/3 | Toxic | ? |
| PDGFRB | platelet-derived growth factor receptor, beta polypeptide | NM_002609 | 5159 | 787743 | 18 | Inhibition (1/3 | Toxic | ? |
| PINK1 | PTEN induced putative kinase 1 | NM_032409 | 65018 | 1086300 | 24 | Inhibition (1/3 | Toxic | ? |
| PIP5KL1 | phosphatidylinositol-4-phosphate 5-kinase-like 1 | NM_173492 | 138429 | 1041730 | 23 | Inhibition (1/3 | Toxic | ? |
| PLK3 | polo-like kinase 3 | NM_004073 | 1263 | 637503 | 14 | Inhibition (1/3 | Toxic | ? |
| PLXNB1 | plexin B1 | NM_002673 | 5364 | 359380 | 8 | Inhibition (1/3 | Toxic | ? |
| PNKP | polynucleotide kinase 3'-phosphatase | NM_007254 | 11284 | 579754 | 13 | Inhibition (1/3 | Toxic | ? |
| PNKP | polynucleotide kinase 3'-phosphatase | NM_007254 | 11284 | 610338 | 14 | Inhibition (1/3 | Toxic | ? |
| PPAP2B | phosphatidic acid phosphatase type 2B | NM_003713 | 8613 | 2545740 | 57 | Inhibition (1/3 | Toxic | ? |
| PPM2C | protein phosphatase 2C, magnesium-dependent, catalytic subunit | NM_018444 | 54704 | 853505 | 19 | Inhibition (1/3 | Toxic | ? |
| PPP1CB | protein phosphatase 1, catalytic subunit, beta isozyme | NM_002709 | 5500 | 347468 | 8 | Inhibition (1/3 | Toxic | ? |
| PPP1R12A | protein phosphatase 1, regulatory (inhibitor) subunit 12A | NM_002480 | 4659 | 352286 | 8 | Inhibition (1/3 | Toxic | ? |
| PPP1R14A | protein phosphatase 1, regulatory (inhibitor) subunit 14A | NM_033256 | 94274 | 1483344 | 33 | Inhibition (1/3 | Toxic | ? |
| PPP1R3A | protein phosphatase 1, regulatory (inhibitor) subunit 3A | NM_002711 | 5506 | 1488760 | 33 | Inhibition (1/3 | Toxic | ? |
| PPP1R3B | protein phosphatase 1, regulatory (inhibitor) subunit 3B | NM_024607 | 79660 | 463643 | 10 | Inhibition (1/3 | Toxic | ? |
| PPP2R1A | protein phosphatase 2 (formerly 2A), regulatory subunit A, alpha isoform | NM_014225 | 5518 | 1132651 | 25 | Inhibition (1/3 | Toxic | ? |
| PPP2R2B | protein phosphatase 2, regulatory subunit B, beta | NM_004576 | 5521 | 299417 | 7 | Inhibition (1/3 | Toxic | ? |
| PPP2R3A | protein phosphatase 2 (formerly 2A), regulatory subunit B'', alpha | NM_002718 | 5523 | 341215 | 8 | Inhibition (1/3 | Toxic | ? |
| PPP2R3C | protein phosphatase 2, regulatory subunit B'', gamma | NM_017917 | 55012 | 763395 | 17 | Inhibition (1/3 | Toxic | ? |
| PPP2R5E | protein phosphatase 2, regulatory subunit B', epsilon isoform | NM_006246 | 5529 | 615991 | 14 | Inhibition (1/3 | Toxic | ? |
| PPP3CC | protein phosphatase 3, catalytic subunit, gamma isozyme | NM_005605 | 5533 | 481639 | 11 | Inhibition (1/3 | Toxic | ? |
| PRKAA1 | protein kinase, AMP-activated, alpha 1 catalytic subunit | NM_006251 | 5562 | 250545 | 6 | Inhibition (1/3 | Toxic | ? |
| PRKCD | protein kinase C, delta | NM_006254 | 5580 | 515429 | 11 | Inhibition (1/3 | Toxic | ? |
| PRKDC | protein kinase, DNA-activated, catalytic polypeptide | NM_001081640 | 5591 | 327567 | 7 | Inhibition (1/3 | Toxic | ? |
| PRPS1L1 | phosphoribosyl pyrophosphate synthetase 1-like 1 | NM_175886 | 221823 | 608358 | 14 | Inhibition (1/3 | Toxic | ? |
| PTP4A3 | protein tyrosine phosphatase type IVA, member 3 | NM_007079 | 11156 | 1604728 | 36 | Inhibition (1/3 | Toxic | ? |
| PTPRO | protein tyrosine phosphatase, receptor type, O | NM_002848 | 5800 | 1328644 | 30 | Inhibition (1/3 | Toxic | ? |
| RIPK1 | receptor (TNFRSF)-interacting serine-threonine kinase 1 | NM_003804 | 8737 | 98467 | 2 | Inhibition (1/3 | Toxic | ? |
| RIPK4 | receptor-interacting serine-threonine kinase 4 | NM_020639 | 54101 | 1551446 | 34 | Inhibition (1/3 | Toxic | ? |
| SGPP2 | sphingosine-1-phosphate phosphatase 2 | NM_152386 | 130367 | 1357905 | 30 | Inhibition (1/3 | Toxic | ? |
| SSH1 | slingshot homolog 1 (Drosophila) | NM_018984 | 54434 | 175691 | 4 | Inhibition (1/3 | Toxic | ? |
| STK17B | serine/threonine kinase 17b | NM_004226 | 9262 | 665026 | 15 | Inhibition (1/3 | Toxic | ? |
| STK39 | serine threonine kinase 39 | NM_013233 | 27347 | 600244 | 13 | Inhibition (1/3 | Toxic | ? |
| TNK2 | tyrosine kinase, non-receptor, 2 | NM_001010938 | 10188 | 121971 | 3 | Inhibition (1/3 | Toxic | ? |
| TRIB3 | tribbles homolog 3 | NM_021158 | 57761 | 474027 | 11 | Inhibition (1/3 | Toxic | ? |
| TSSK2 | testis-specific serine kinase 2 | NM_053006 | 23617 | 383753 | 9 | Inhibition (1/3 | Toxic | ? |
