# Supplementary material for: Kinome-Wide RNA Interference Screening Identifies Mitogen-Activated Protein Kinases and Phosphatidylinositol Metabolism as Key Factors for Rabies Virus Infection
Source: mSphere. 2019 May 22;4(3):e00047-19. doi: 10.1128/mSphere.00047-19 (PMC6531879; doi:10.1128/mSphere.00047-19)
Supplement: TABLE S3 [file mSphere.00047-19-st003.docx]

| Gene Symbol | Original siRNA | C911 Target |
| --- | --- | --- |
| MAP2K7 | GACAGUUUCCCUACAAGAAUU | GACAGUUUCCGAUCAAGAAUU |
| DUSP5 | GAGUGUUGCGUGGAUGUAAUU | GAGUGUUGCGACCAUGUAAUU |
| RPS6KA5 | GCUGAGAUAUCUAGGAGAAUU | GCUGAGAUAUGAUGGAGAAUU |
| PIP5K1C | GCGUCGUGGUCAUGAACAAUU | GCGUCGUGGUGUAGAACAAUU |
| MTM1 | GAGUGGGAAACGAAAUAAAUU | GAGUGGGAAAGCUAAUAAAUU |
| MINPP1 | CAUAGAUGAUGCAAAGGUAUU | CAUAGAUGAUCGUAAGGUAUU |
| PIK3C2G | CCAUCUACCAGCUAAUCAAUU | CCAUCUACCACGAAAUCAAUU |
| PRPF4B | CAGUUGAUUUAAGAGGUAAUU | CAGUUGAUUUUUCAGGUAAUU |
| RNGTT | GGAACUUUUUCGUCGGUAUUU | GGAACUUUUUGCACGGUAUUU |
| NRBP2 | GGCUCAGUCUUAGAGAUUUUU | GGCUCAGUCUAUCAGAUUUUU |
| NEK4 | CCAACAUUGUCACCUACAAUU | CCAACAUUGUGUGCUACAAUU |
| ERN1 | CCUGCGCUAUCUGACCUUCUU | CCUGCGCUAUGACACCUUCUU |
| FYN | GGGAUGAUAUGAAAGGAGAUU | GGGAUGAUAUCUUAGGAGAUU |
