# Supplementary material for: Kinome-Wide RNA Interference Screening Identifies Mitogen-Activated Protein Kinases and Phosphatidylinositol Metabolism as Key Factors for Rabies Virus Infection
Source: mSphere. 2019 May 22;4(3):e00047-19. doi: 10.1128/mSphere.00047-19 (PMC6531879; doi:10.1128/mSphere.00047-19)
Supplement: TABLE S4 [file mSphere.00047-19-st004.docx]

| **Compound Name** | **Reported targets** | **Manu-facture** | **Library** | **Cat. No.** |
| --- | --- | --- | --- | --- |
| Lithium Chloride | GSK3, Inositol / monophosphatase | Sigma | LOPAC | L 4408 |
| D-609 potassium | PIPLC | Sigma | LOPAC | T 8543 |
| **Wortmannin** | **PI3K (cross-react with mTOR, DNA-PKCs, ATM, ATR and PI4K)** | **Sigma** | **LOPAC** | **W 1628** |
| Idelalisib, Zydelig | PI3K | SelleckChem | Selleck_AC | S2226 |
| Me-3,4-dephostatin | Inhibitor of protein tyrosine phosphatase 1B and SHPTP-1 | Sigma-Aldrich | LOPAC | M 9440 |
| U0126 | Inhibitor of MEK1/2 | Sigma-Aldrich | LOPAC | U-120 |
| BIRB 796 (Doramapimod) | Inhibitor of p38α/β/γ/δ MAPK (cross-react with JNK2, c-RAF, Fyn, Lck, ERK-1, SYK and IKK2) | Selleck | Selleck_AC | S1574 |
| NSC 663284 | Inhibitor of Cdc25 dual specificity phosphatases Cdc25A, Cdc25B2 and Cdc25C ( cross-react with VHR and PTP1B phosphatases) | Tocris Bioscience | Tocris 2007 | 1867 |
| **Ro 31-8220 mesylate** | **Inhibitor of PKC (cross-react with MAPKAP-K1b, MSK1, GSK3b and S6K1)** | **Tocris Bioscience** | **Tocris 2007** | **2002** |
| SD 169 | Inhibitor of p38α | Tocris Bioscience | Tocriscreen | 2938 |
| U0126, nd | Inhibitor of MEK1/2 | Tocris Bioscience | Tocriscreen | 1144 |
| PD 98059 | Inhibitor of MAPKK | Tocris Bioscience | Tocriscreen | 1213 |
| SL 327 | Inhibitor of MEK1/2 | Tocris Bioscience | Tocriscreen | 1969 |
| **PD 198306** | **Inhibitor of MEK (cross-react with ERK, c-Src, cdks and PI3K)** | **Tocris Bioscience** | **Tocriscreen** | **2605** |
