# Supplementary figures and images for: Kinome-Wide RNA Interference Screening Identifies Mitogen-Activated Protein Kinases and Phosphatidylinositol Metabolism as Key Factors for Rabies Virus Infection
Source: mSphere. 2019 May 22;4(3):e00047-19. doi: 10.1128/mSphere.00047-19 (PMC6531879; doi:10.1128/mSphere.00047-19)

A

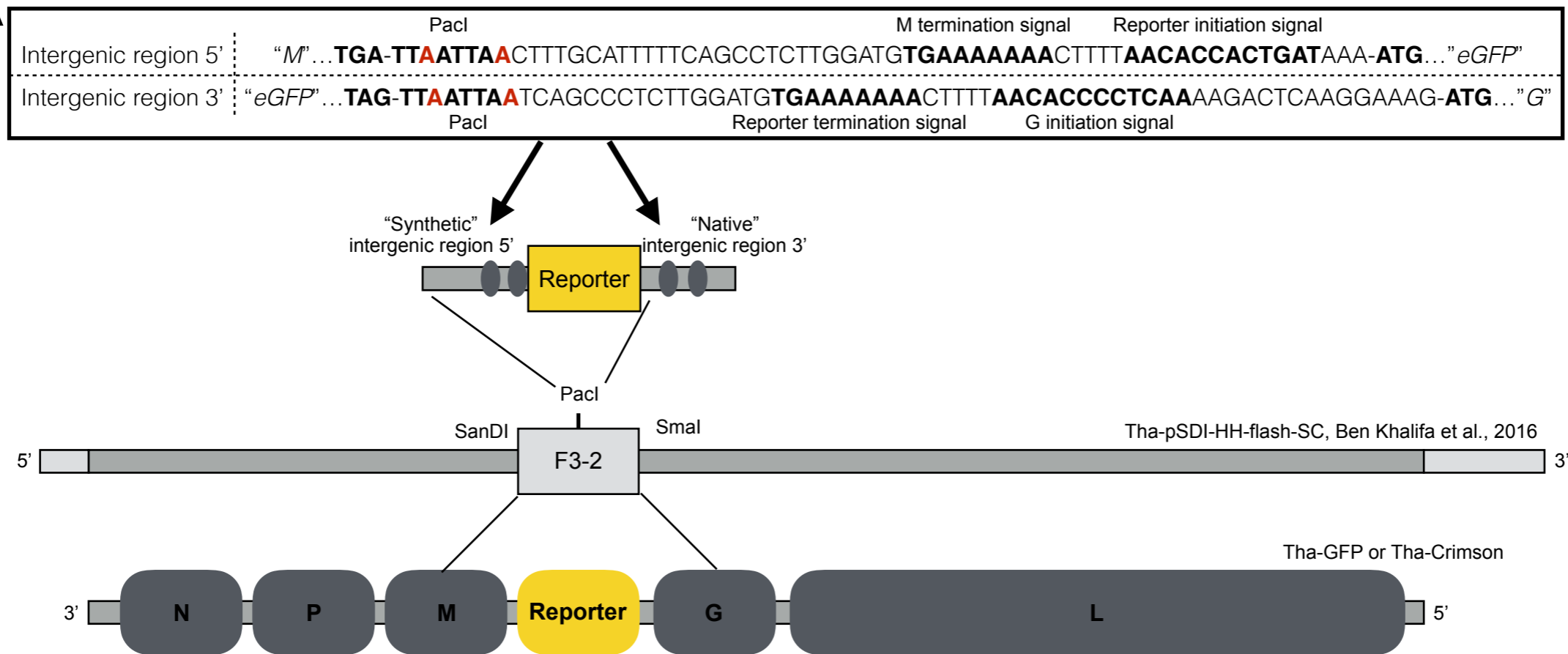

B

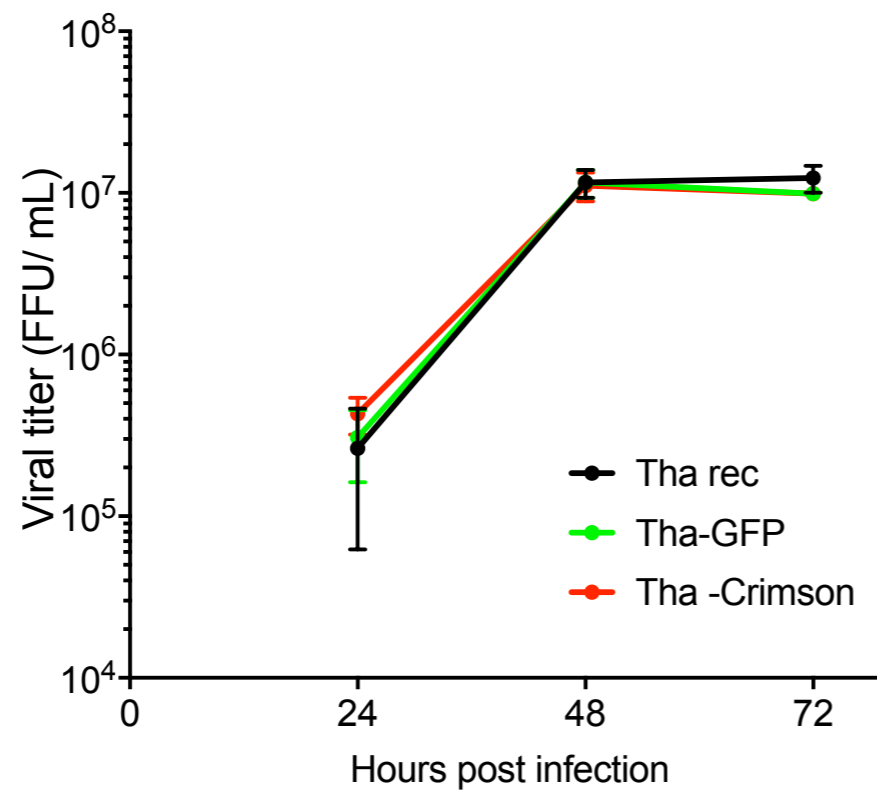

Supplement: FIG S1 [file mSphere.00047-19-sf001.pdf]

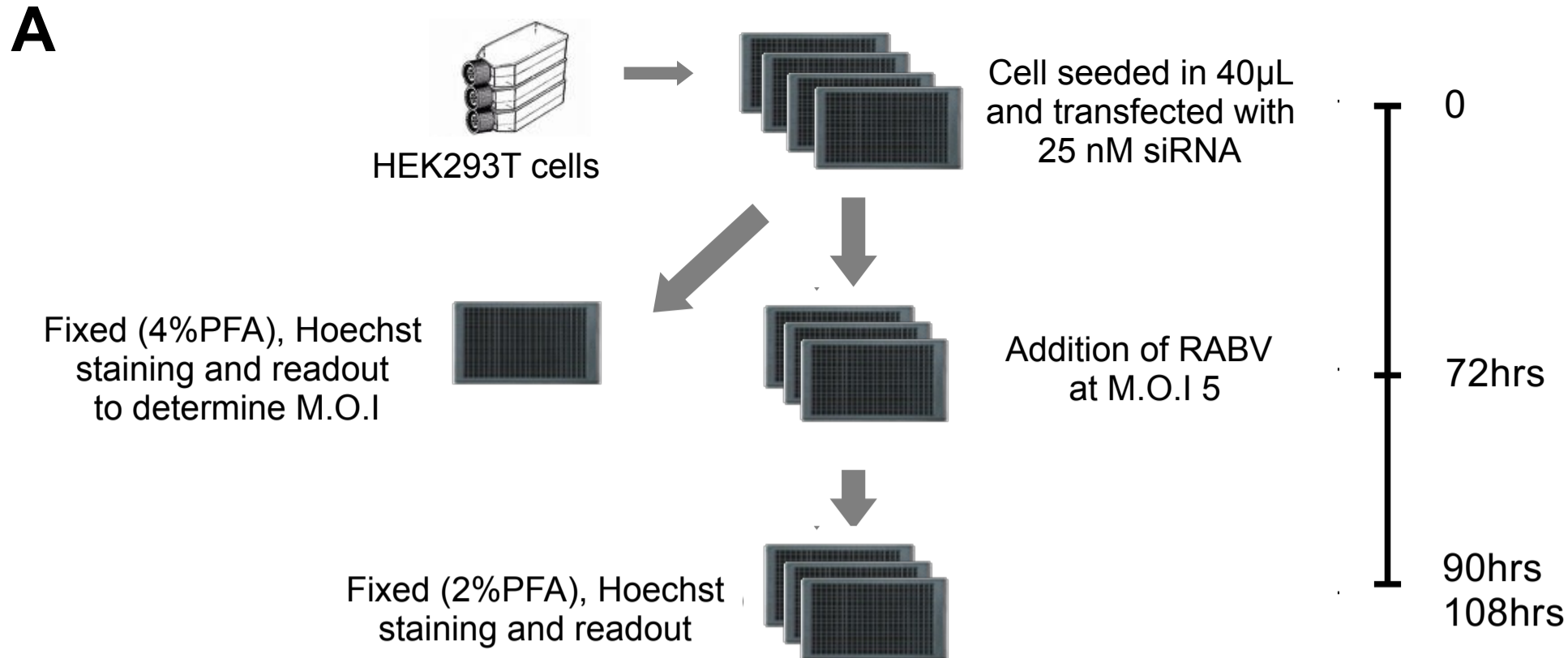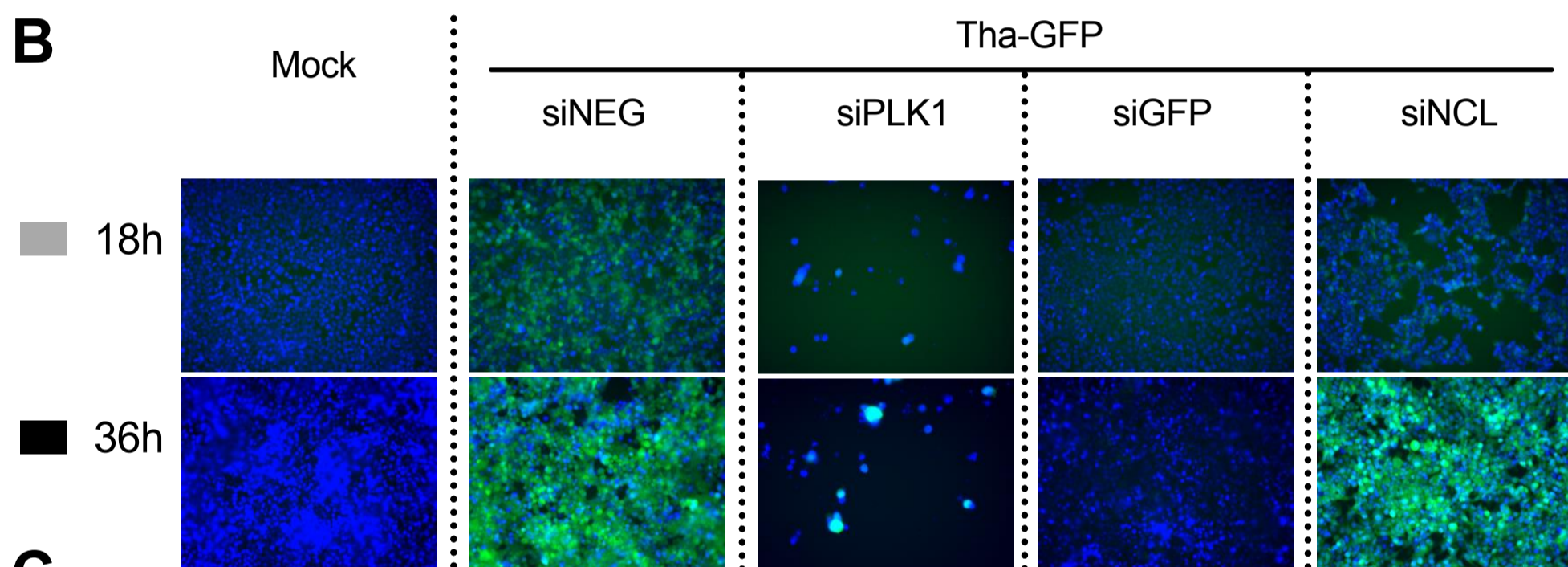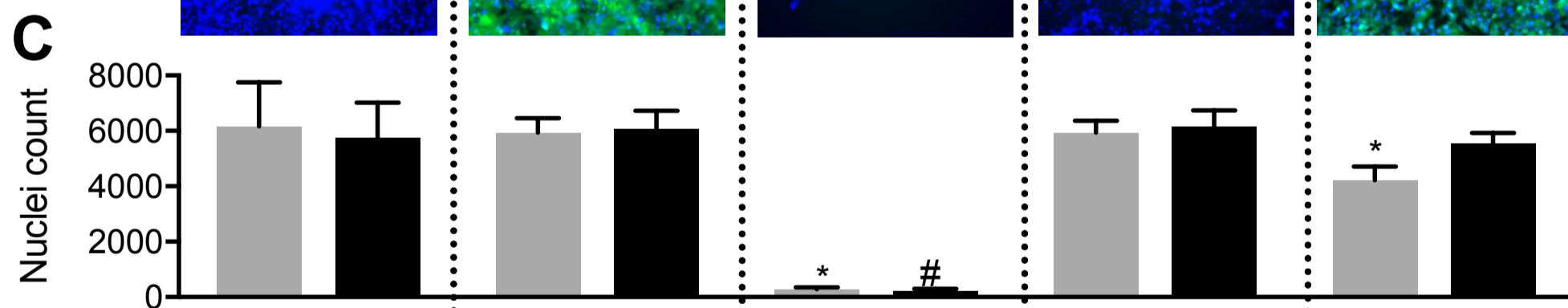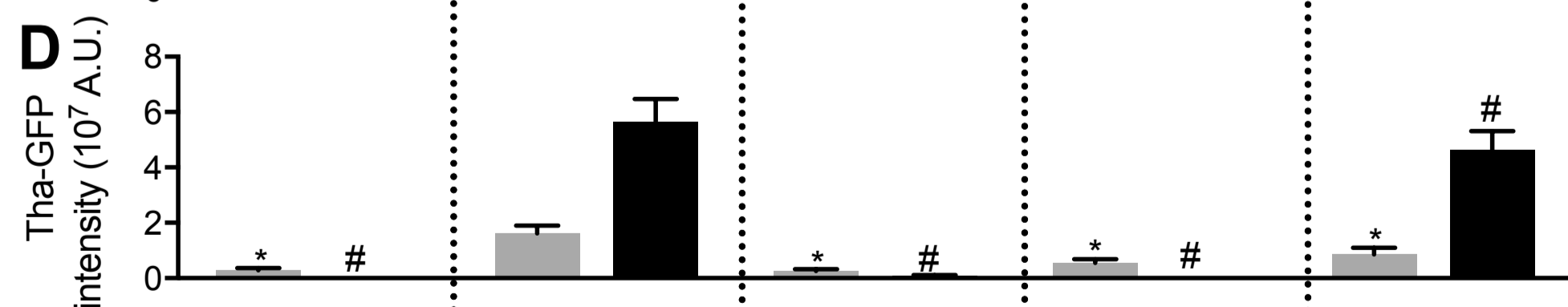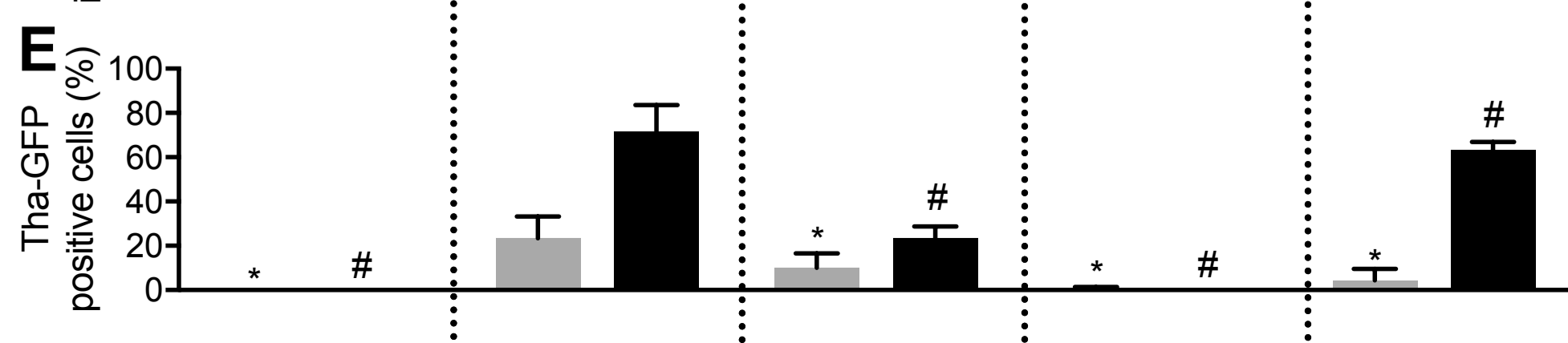

Supplement: FIG S2 [file mSphere.00047-19-sf002.pdf]

# PHOSPHATIDYLINOSITOL SIGNALING SYSTEM

18h only

18h and 36h

36h only

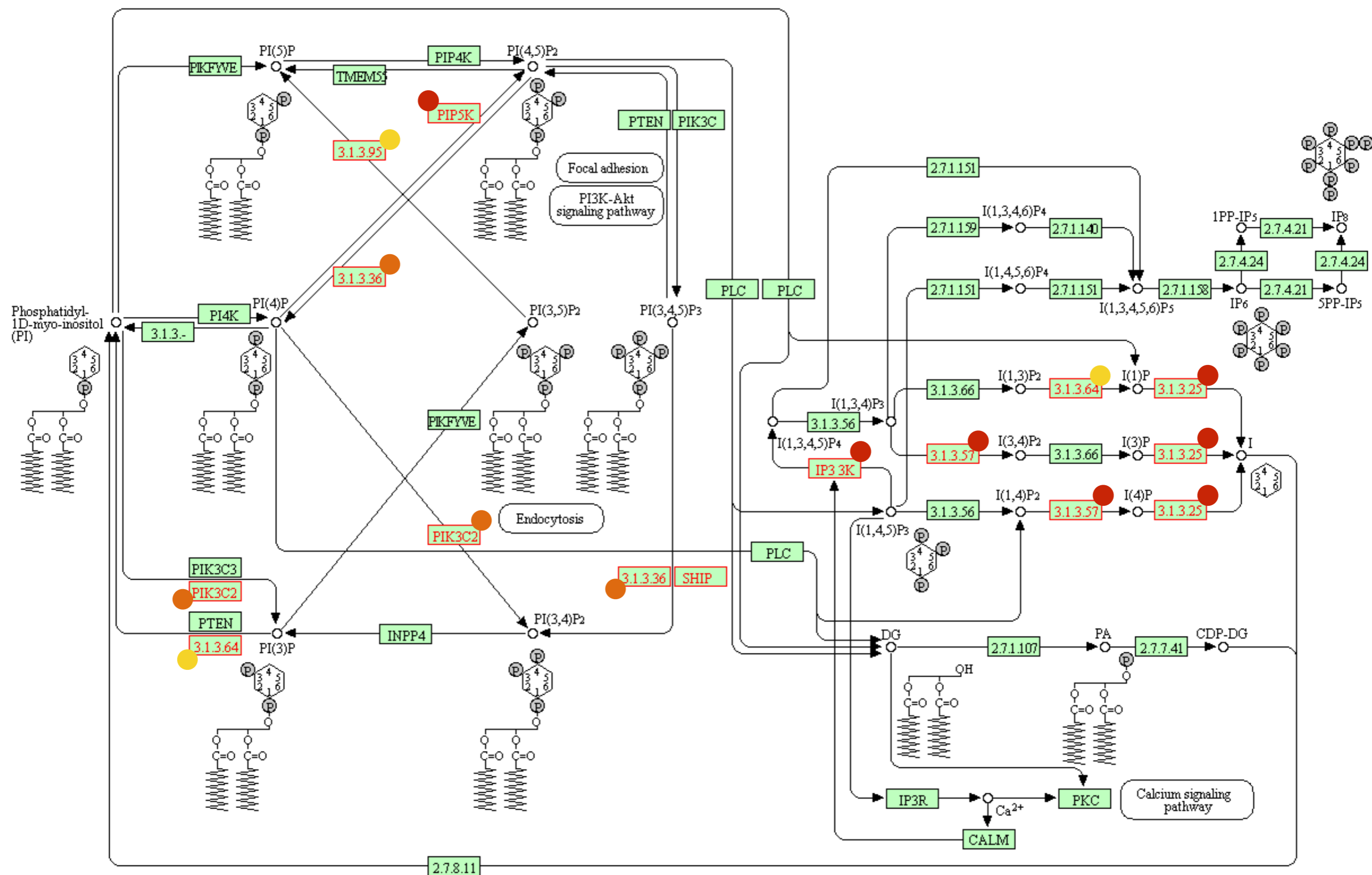

Supplement: FIG S3 [file mSphere.00047-19-sf003.pdf]

# FRUCTOSE AND MANNOSE METABOLISM

● 18h only

● 18h and 36h

● 36h only

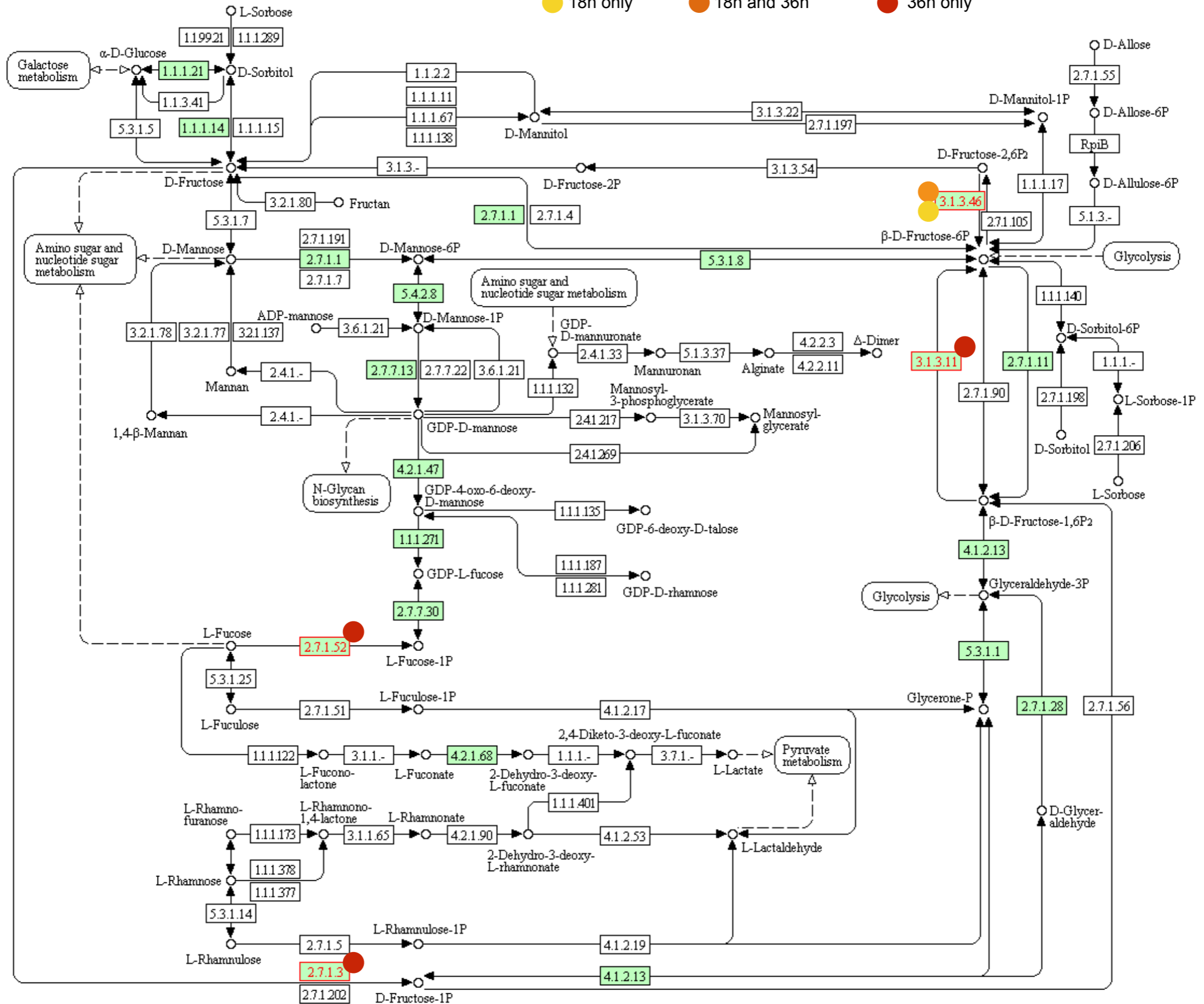

Supplement: FIG S4 [file mSphere.00047-19-sf004.pdf]

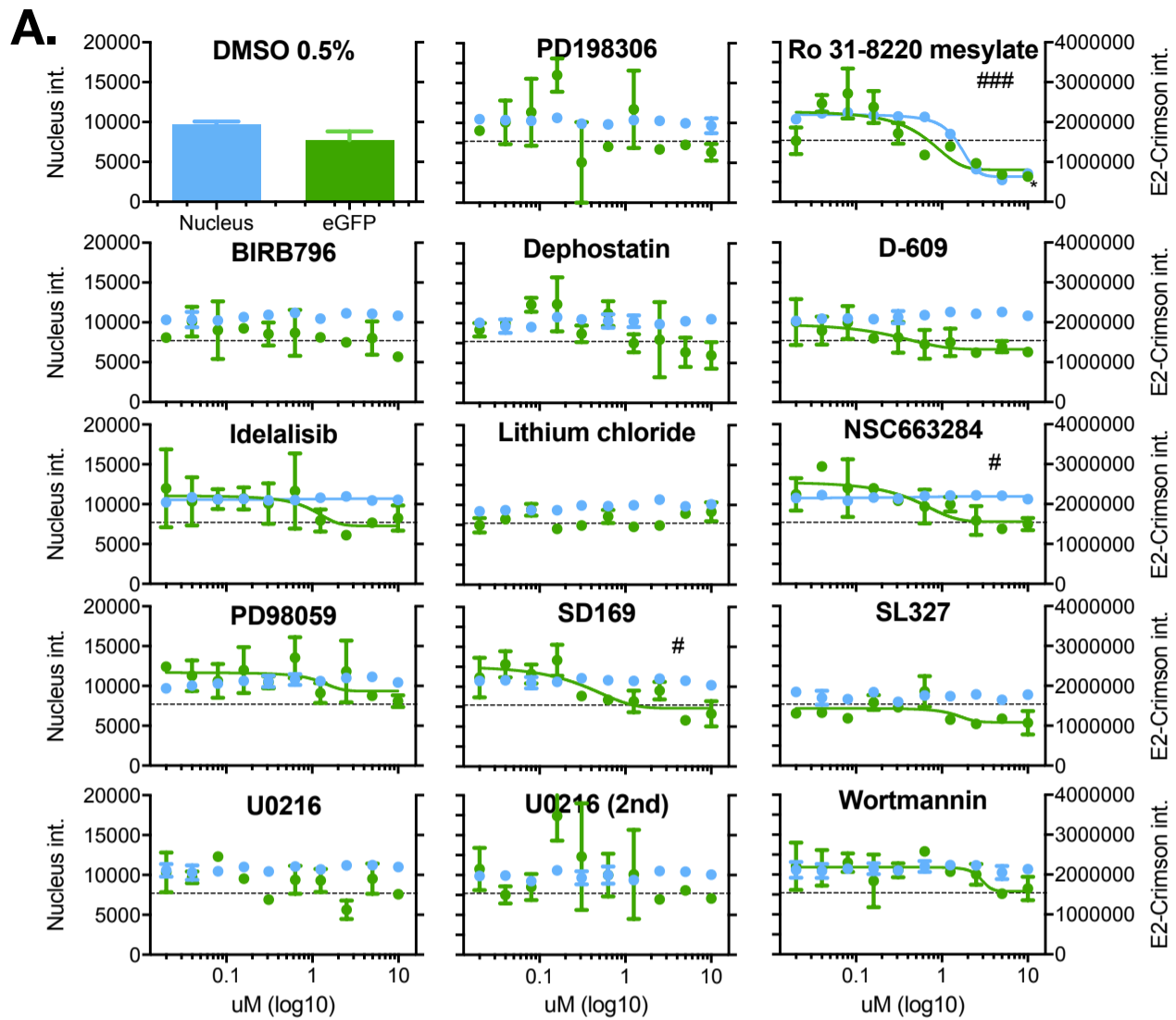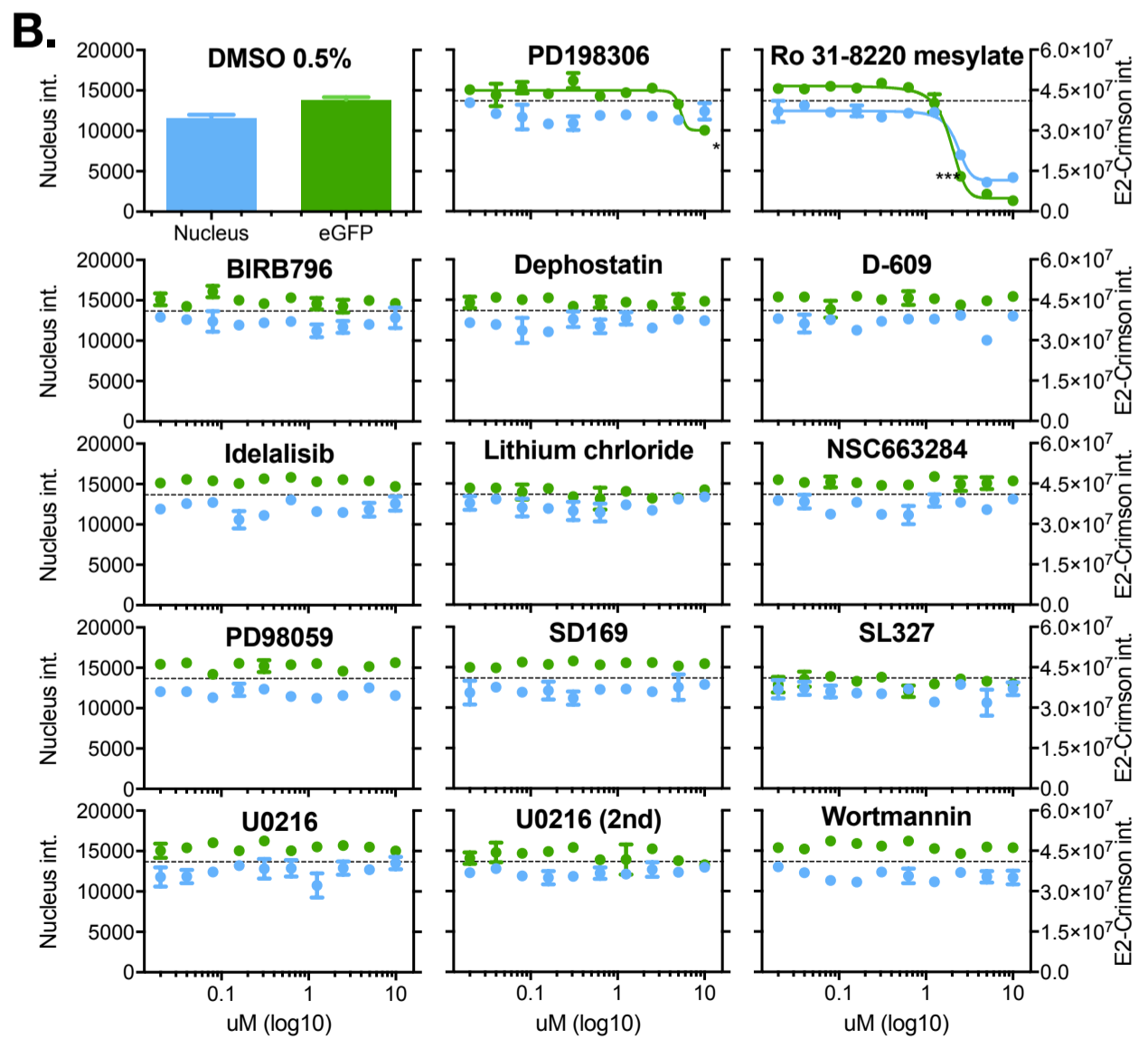

Supplement: FIG S5 [file mSphere.00047-19-sf005.pdf]

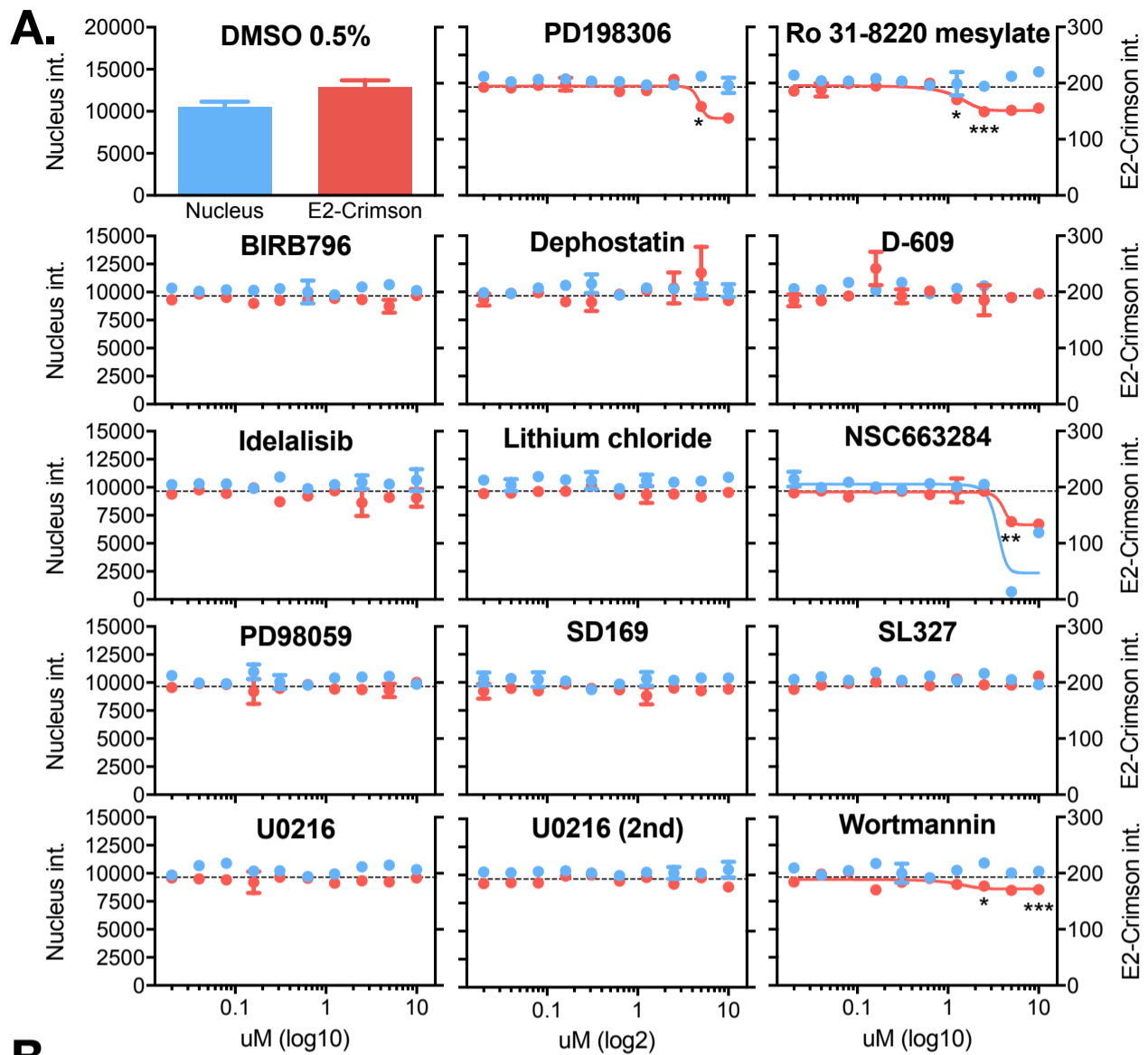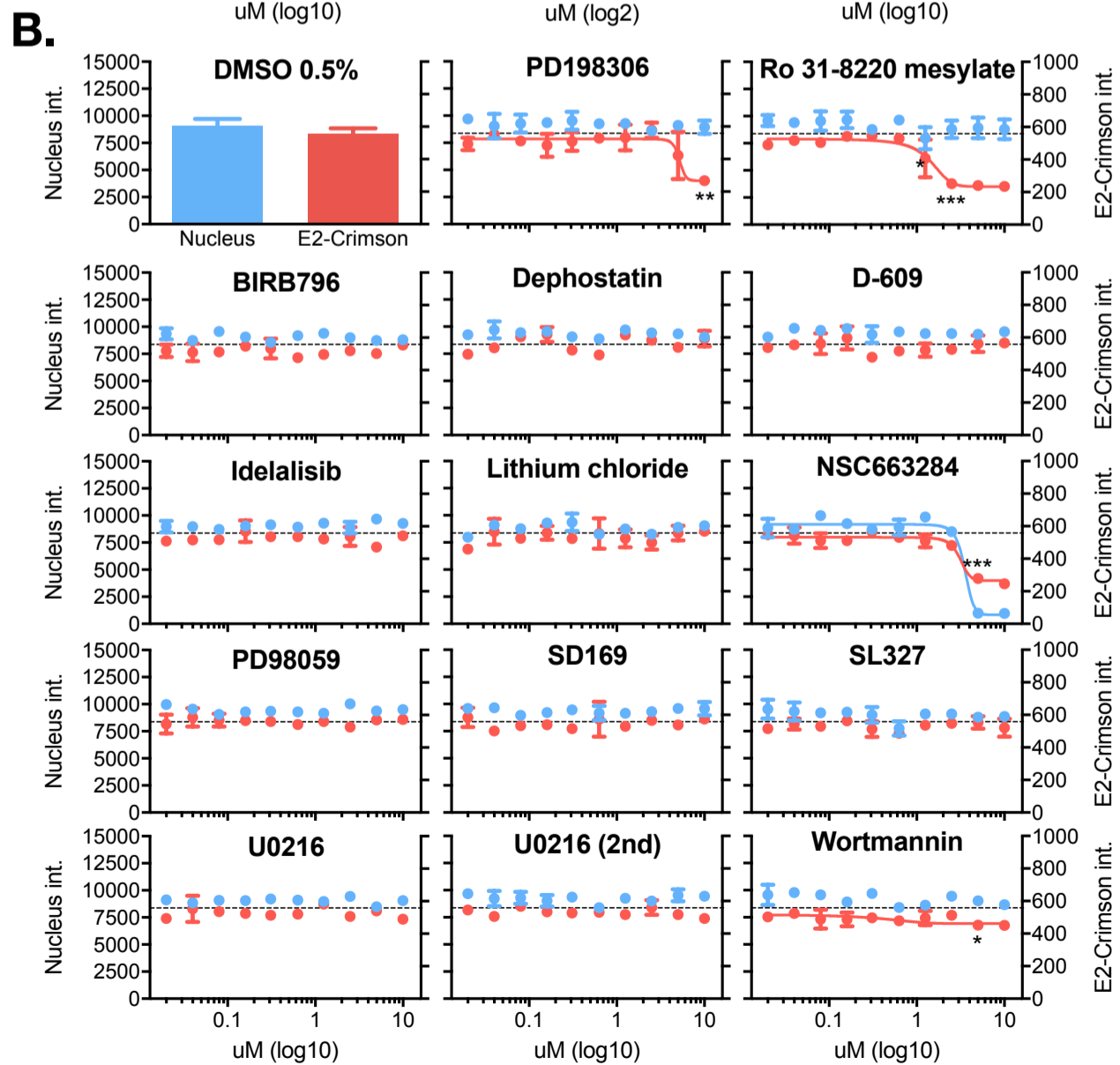

Supplement: FIG S6 [file mSphere.00047-19-sf006.pdf]
